# Study on the total synthesis of velbanamine: Chemoselective dioxygenation of alkenes with PIFA via a stop-and-flow strategy

**DOI:** 10.3762/bjoc.9.113

**Published:** 2013-05-23

**Authors:** Huili Liu, Kuan Zheng, Xiang Lu, Xiaoxia Wang, Ran Hong

**Affiliations:** 1 CAS Key Laboratory of Synthetic Chemistry of Natural Substances, Shanghai Institute of Organic Chemistry, Chinese Academy of Sciences, Shanghai 200032, China; 2 College of Chemistry and Life Sciences, Zhejiang Normal University, Jinhua, Zhengjiang 321004, China

**Keywords:** alkene, desymmetrization, dioxygenation, lactone, PIFA, velbanamine

## Abstract

A “stop-and-flow” strategy was developed for the chemoselective dioxygenation of alkenes with a PIFA-initiated cyclization. This method is conceived for the desymmetrization of seco-diene, and a series of substituted 5-hydroxymethyl-γ-lactones were constructed after hydrolysis. This strategy also differentiates terminally substituted alkenes and constitutes a potentially novel synthetic approach for the efficient synthesis toward velbanamine.

## Introduction

**Scheme 1:**
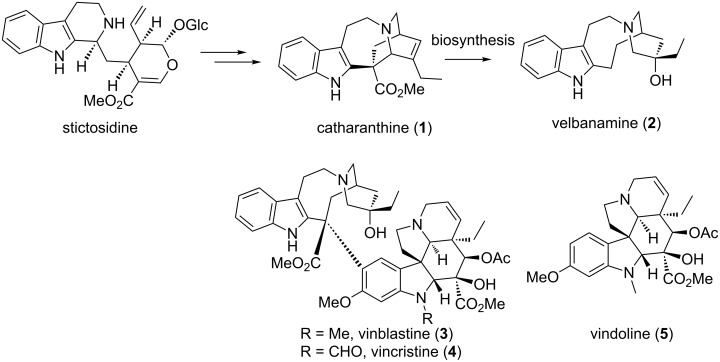
Biogenetic origin of *Vinca* alkaloids.

Stictosidine-derived indole alkaloids comprise a large category of alkaloids with diverse biological activities [1]. Their complex chemical structures and applications in pharmaceuticals have sparked numerous synthetic efforts in the past few decades. Dimeric indole alkaloids such as vinblastine (**3**) (Scheme 1) still inspire synthetic chemists to develop novel strategies for their efficient synthesis [2–11]. Isolated from the pantropical plant *Catharanthus roseus*, vinblastine (**3**) and vincristine (**4**) have potent antitumor activity and are clinically utilized in the treatment of Hodgkin’s disease and leukemia [12–14]. Fragmentation of **3** under acidic conditions delivers two structural units, desacetylvindoline and velbanamine (**2**) [15]. The reassembling of catharanthine (**1**) and vindoline (**5**) into the parent alkaloids **3** and **4** by using FeCl_3_-promoted oxidative coupling supports the biogenesis of heterodimeric indole alkaloids [11]. Interestingly, velbanamine (**2**) was later identified in leaves and twigs of *Tabernaemontana eglandulosa* in 1984 [16]. Therefore, the syntheses of velbanamine (**2**) and structurally closely related alkaloids may be important for the syntheses of their dimeric alkaloids. The modification of these alkaloids in the context of clinical drug development still poses a challenge to synthetic and medicinal chemists.

**Scheme 2:**
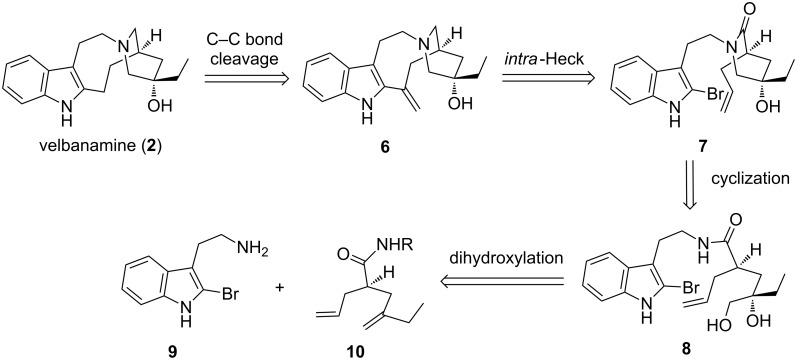
Synthetic strategy for velbanamine based on chemoselective dioxygenation.

Since the first racemic synthesis of velbanamine (**2**) was disclosed by Büchi and co-workers in 1968, four racemic syntheses and two enantioselective syntheses have been reported in spite of several synthetic efforts toward the core structure [17–24]. The practical synthesis of velbanamine (**2**) and a general approach toward structurally related alkaloids remain an intriguing task in the synthetic community. Here, we would like to disclose our recent efforts on method development toward the efficient construction of velbanamine-type indole alkaloids. As shown in Scheme 2, an intramolecular Heck reaction (*via* 9-*exo* manner) would finalize the 9-membered ring, which was biogenetically derived from a retro-Mannich reaction from catharanthine (Scheme 1) [25]. The terminal alkene **6** can be disconnected to give amide **7**, which may be derived from 2-bromotryptamine **9** and seco-diene **10**. Clearly, the chemoselective dioxygenation of **10** is the main theme in our synthetic endeavor. From here, we expect that current synthetic methods may provide a general basis for similar important structures, such as isovelbanamine and cleavamine.

Dioxygenation has been adopted as a powerful method to transform an alkene into vicinal functional groups in the synthesis of natural products and pharmaceuticals. Numerous synthetic methods have been developed for this purpose, mediated by metals such as Os, Mn, Pd, Ru, Fe, and Ag [26–37]. On the other hand, the metal-free hypervalent iodine(III)-mediated reactions have recently enjoyed a renaissance attracting extensive investigations [38–39]. It is particularly interesting in the case where iodolactones, obtained from the iodolactonization of alkenes in the presence of iodine, can be converted into hydroxylactone upon saponification [40–41]. This appealing metal-free protocol evolved into enantioselective iodolactonization by using chiral amides or esters. The catalytic asymmetric halogen lactonization, as an emerging area, also attracted a lot of attention in recent years [42–44].

The two terminal alkenes in compound **10** pose a challenge for chemoselective dioxygenation or iodolactonization. To address this problem, we turn our attention to differentiating the two types of terminal alkenes. Although iodolactonization as exemplified in Kita’s elegant synthesis of rubrenolide resulted in a practical approach on desymmetrization [45], the subsequent hydrolysis still required an extra step to reveal the proper hydroxy group.

## Results and Discussion

### The pathway of cyclization

**Scheme 3:**
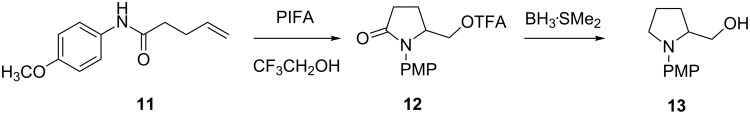
Intramolecular oxyamidation of alkene **11** with phenyliodine(III)-bis(trifluoroacetate) (PIFA) by Tellitu, Dominguez and co-workers [46].

**Scheme 4:**
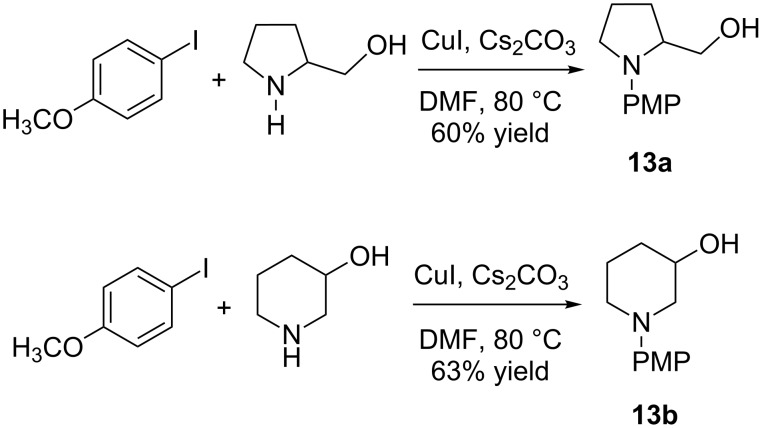
Copper-catalyzed amination of aryliodide.

Recently, Tellitu, Dominguez, and co-workers reported an intramolecular oxyamidation of alkene **11** with phenyliodine(III)-bis(trifluoroacetate) (PIFA) (Scheme 3) [46]. The lactam **12** was originally assigned as an unstable intermediate, which should be subsequently reduced to pyrrolidine **13**. It was particularly striking to us that the arene unprecedentedly stabilizes the primary carbon cation through a neighboring participation in the mechanism proposed by Tellitu et al. To resolve the confusion, we synthesized the corresponding pyrrolidine through an alternative approach (Scheme 4). The amination of *para*-methoxyaryliodide with 2-hydroxymethylpyrrolidine in the presence of Cs_2_CO_3_ and a catalytic amount of copper iodide in DMF afforded **13a** in 60% isolated yield [47]. To our surprise, the proton NMR of **13a** was distinct from that of the originally proposed **13** by Tellitu et al. [46]. Based on the Baldwin rule, both 5-*exo-trig* and 6-*endo-trig* are favorable in the cyclization. Meanwhile, the ring expansion may occur to constitute the functionalized piperidine ring, resembling Cossy’s endeavor to synthesize velbanamine [48]. In this regard, the same coupling condition was applied for the reaction of 3-hydroxypiperidine with *para*-methoxyaryliodide. Unexpectedly, the spectrum of the resulting amine **13b** was not consistent with that of **13**.

**Scheme 5:**
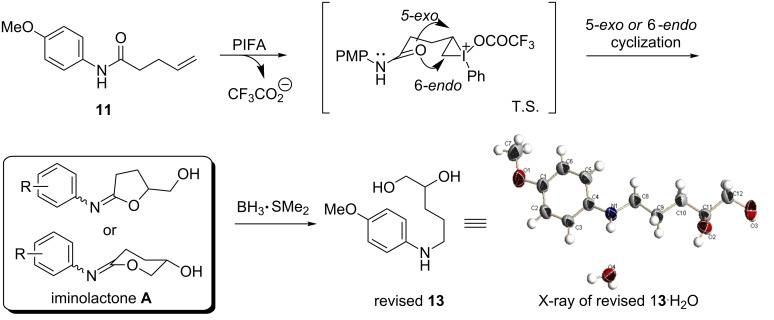
Revised PIFA-promoted cyclization of amide **11**.

Based on the general iodolactonization principle, the C–O dipole in an amide is aligned for a favorable nucleophilic addition due to the “double-bond character” in a planar amide [41]. In the PIFA-promoted cyclization, lactonization was more likely to be a prominent process than lactamization. Following Tellitu’s procedure [46], we re-synthesized **13** and subjected it to the proton NMR in the presence of D_2_O. Three proton signals were exchanged with deuterium. X-ray analysis further contributed to the elucidation of the linear structure of **13** as shown in Scheme 5 [49]. The revised structure **13** implies that the amide **11** did not undergo the oxyamidation under the previously defined reaction conditions. Alternatively, it appears that iodolactonization of the amide dominated when compound **11** was subjected to the oxidation conditions of PIFA in CF_3_CH_2_OH. Mechanistically, the terminal double bond is activated by hyperiodine or via a halogenium-like intermediate [50–52]. The subsequent intramolecular ring-closing reaction in a 5-*exo* or 6-e*ndo* manner delivers iminolactones **A**. The reduction with borane gives hydroxyamine **13**. In this new proposal, the iminolactone **A** could be unstable and difficult to purify according to Tellitu’s findings [46]. Similar stability of iminolactone is documented in the literature [53–55].

**Scheme 6:**
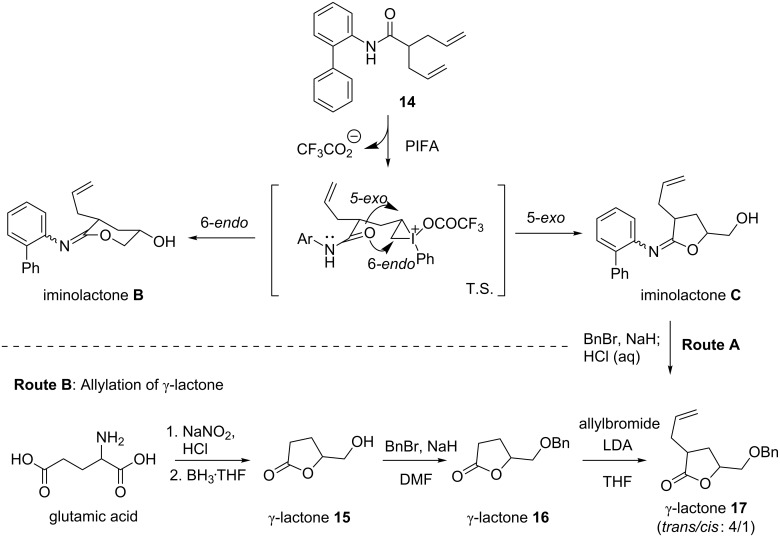
PIFA-promoted cyclization to synthesize lactone.

Although we have established the amide-assisted dioxygenation of an alkene, the structure of the iminolatone **A** is still a mystery. In principle, both 5-*exo-trig* and 6-e*ndo-trig* are favorable during the cyclization (Scheme 6). However, the instability of the iminolactone **A** impedes further characterization, and consequently, the possible cyclization mode is difficult to clarify. We envisioned that the steric hindrance of the amide would prevent the hydrolysis of the iminolactone during the work-up stage. After several experiments, *ortho*-biphenylamide **14** was chosen as a starting point. To our delight, two isomers of iminolactone were separated on a silica-gel column. After the treatment of benzyl bromide in the presence of NaH, the acidic work-up delivered two diastereoisomers of lactone **17** (Route A). This was further confirmed by an alternative synthesis based on a known procedure [56], in which 3-substituted-γ-lactone **17** was derived from the stereoselective alkylation of Bn-protected 5-hydroxymethyl-γ-lactone **16** (Route B). Two diastereoisomers of the *trans*-isomer of **17** (ratio 4/1) were identical with compounds from the PIFA-promoted cyclization (Scheme 6, Route A). Based on these conclusive experiments, we believe that the 5-e*xo-trig* cyclization is the favorable pathway during the PIFA-mediated amide-cyclization of alkene. Two isomers after cyclization were then attributed to the stereoisomers of the C=N double bond in the iminolactone **C**. This notion is also consistent with numerous reports on the iodocyclization with amide [41].

### Chemoselective dioxygenation

**Figure 1:**
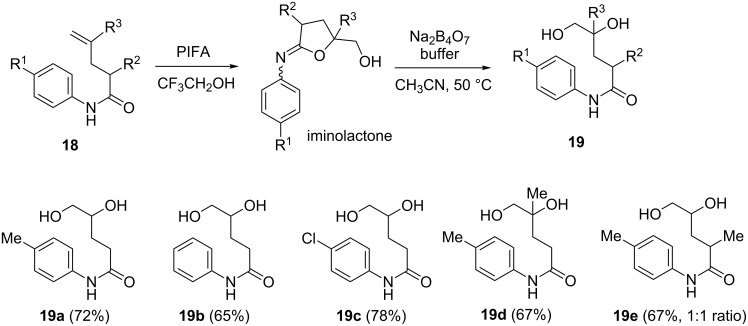
Hydrolysis of iminolactone **18** under basic conditions.

The structural assignment of **13** and the verification of the reaction pathway encouraged us to explore the synthetic potential of the dioxygenation of alkene with the assistance of an amide. The dioxygenation can be “*stopped*” after the first PIFA-promoted cyclization to form an iminolactone (such as the intermediate **C** in Scheme 6). Thus, if the original amide group could be recovered, it may “*flow*” into the second round of alkene dioxygenation. This “stop-and-flow” approach easily differentiates two alkene groups during the synthetic endeavor. In our experiment, after cyclization of amide **18a**, the corresponding iminolactone was hydrolyzed under Mukaiyama’s conditions (sat. Na_2_B_4_O_7_ buffer in CH_3_CN) [53] to give amide **19a** in 72% yield [57]. The different substituents on aniline (Figure 1) slightly deteriorated the isolated yield. The geminal bis-substituted alkene gave a moderate yield of the corresponding dihydroxyamide **19d**. The α-methylated substrate was also dioxygenated albeit in a low diastereoselectivity (dr 1:1).

**Scheme 7:**
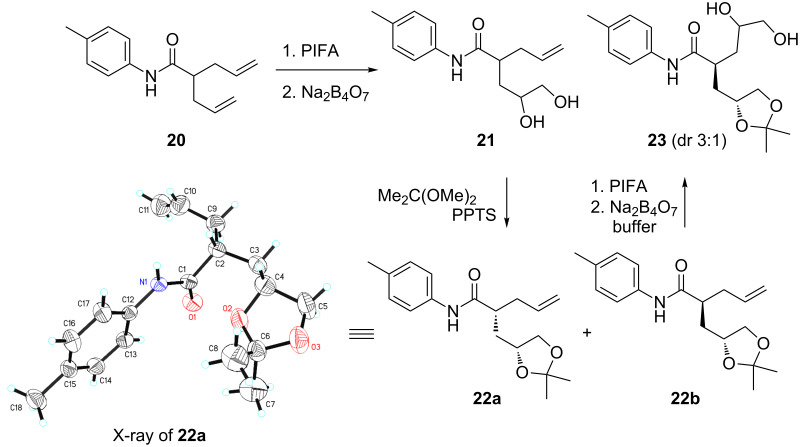
“Stop-and-flow” strategy for the stepwise dioxygenation of alkenes.

When two alkene groups co-existed as in compound **20**, desymmetrization delivered the mono-dioxygenated product **21** in a dr ratio of 1:1 (Scheme 7). The less polar **22a** was confirmed by X-ray diffraction [58]. The second alkene was further dioxygenated by repeating the previous protocol to deliver **23** with a 3/1 diastereoselectivity from **22b**. The encountered poor chemoselectivity under conventional metal-mediated conditions for the dioxygenation of seco-dienes such as **20** implies that this iterative method may find usage in organic synthesis. In this mode, we can install four hydroxy groups on a seco-diene with different protecting groups. Although several research groups have investigated the desymmetrization of seco-diene by using iodolactonization [59–64], our strategy here proves the concept of “stop-and-flow” to functionalize alkenes step-by-step with a simple hydrolysis in between.

### Synthetic applications

**Scheme 8:**
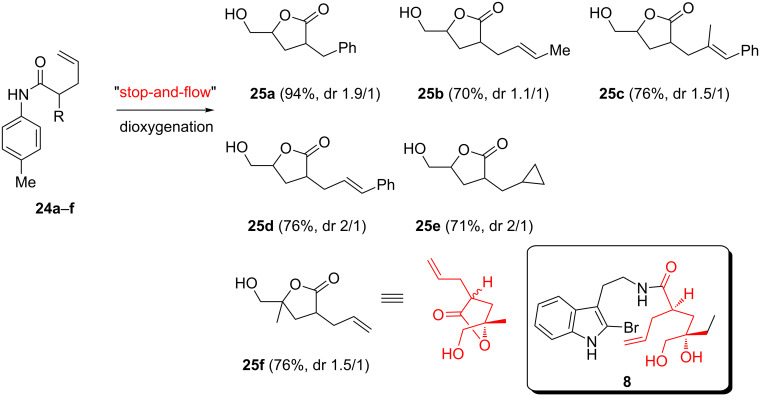
“Stop-and-flow” strategy for the construction of γ-lactone derivatives.

After a simple acidic work-up, the established desymmetrization process can also be applied to prepare 3-alkyl-5-hydroxymethyl-γ-lactone, which has been widely found in natural products and compounds of pharmaceutical interest [65–68]. The usual methods including the alkylation at C-3 or the iodolactonization of amides or esters comprises multiple production steps, such as the hydrolysis of the halogen compounds and the protecting groups. However, the direct dioxygenation can easily construct γ-lactones by a simple acidic work-up as shown in Scheme 8. For example, when **24a** (R = Bn) was subjected to the PIFA-mediated cyclization, the desired γ-lactone **25a** was isolated after hydrolysis in 94% yield with a ratio of trans/cis of 1.9:1. Compound **25a** can be easily converted into the key intermediate in the synthesis of indinavir, a protease inhibitor used as a component of a highly active antiretroviral therapy to treat HIV infection and AIDS [69]. When two alkene groups exist in **24b–d**, the terminal alkene preferentially underwent cyclization to deliver a variety of 3-susbtituted-γ-lactones (**25b–d**). For comparison, the *m*CPBA-epoxidation approach in literature always demonstrated that electron-rich alkenes are more reactive leading to the reversal of chemoselectivity [70–71]. A cyclopropane group was also tolerated during this operation and lactone **25e** was obtained in 71% yield with a dr ratio of 2:1.

Product **25f** was particularly interesting since the “stop-and-flow” strategy can differentiate two terminal alkenes. The more reactive geminally substituted alkene was dioxygenized to deliver all functional groups required in **8**, which was designed as a key intermediate in our synthetic plan toward velbanamine (Scheme 2). The stereoselectivity still awaits further improvement. Nevertheless, as a proof of concept, the current approach allows us to develop an efficient synthetic route to access challenging synthetic targets.

## Conclusion

In summary, with a comprehensive validation of PIFA-promoted cyclization of alkenes, a synthetically useful desymmetrization approach via the dioxygenation of alkenes was developed. The “stop-and-flow” strategy allows us to easily functionalize seco-dienes step-by-step. Moreover, this approach also chemoselectively functionalizes terminal alkenes instead of internal ones. Substituted 5-hydroxymethyl-γ-lactones have been constructed in a protecting-group-free manner. The synthetic application in the efficient synthesis of velbanamine-type indole alkaloids as well as the enantioselective desymmetrization are currently pursued in our laboratory and will be reported in due course.

## Supporting Information

File 1 Experimental descriptions, analytical and X-ray data.
