# Supplementary material for: Study on the total synthesis of velbanamine: Chemoselective dioxygenation of alkenes with PIFA via a stop-and-flow strategy
Source: Beilstein J Org Chem. 2013 May 23;9:983–90. doi: 10.3762/bjoc.9.113 (PMC3678578; doi:10.3762/bjoc.9.113)
Supplement: File 1 — Experimental descriptions, analytical and X-ray data. [file Beilstein_J_Org_Chem-09-983-s001.pdf]

Supporting information

for

Study on the total synthesis of velbanamine: Chemoselective dioxygenation of alkenes with PIFA via a stop-and-flow strategy

Huili Liu[1,2], Kuan Zheng[1], Xiang Lu[1], Xiaoxia Wang[*,2] and Ran Hong[*,1]

Address: [1]CAS Key Laboratory of Synthetic Chemistry of Natural Substances, Shanghai Institute of Organic Chemistry, Chinese Academy of Sciences, Shanghai 200032, China and [2]College of Chemistry and Life Sciences, Zhejiang Normal University, Jinhua, Zhengjiang 321004, China

Email: Ran Hong[*] - rhong@sioc.ac.cn; Xiaoxia Wang[*] - wangxiaoxia@zjnu.cn

∗Corresponding author

Experimental descriptions, analytical and X-ray data

Contents

I. General procedure……………………………………………….…………..……S2

II. Experimental……….…………………………………………………….………S2

III. X-ray data for the revised compound **13**. …………………..………………… S19

IV. X-ray data for compound **22a** ……………………………………………….... S20

V. ^{1}H NMR and ^{13}C NMR spectra…………………………………………………S21

VI. References…………………………………………….................................S56

I. General procedure:

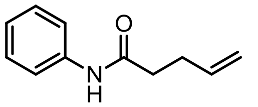

All the reactions were carried out under N_2 atmosphere unless otherwise stated. All the solvents were dried by using standard procedures and distilled before use. All reagents were used as received unless otherwise stated.

^{1}H NMR and ^{13}C NMR were recorded on Varian mercury–400 MHz spectrometers, Varian mercury–300 MHz spectrometers or Bruker AM–400 (400 MHz) spectrometers in $CDCl_3$. The chemical shift (δ) of ^{1}H NMR is given in ppm relative to TMS (δ = 0.00 ppm). The chemical shift (δ) of ^{13}C NMR is given in ppm relative to $CDCl_3$ (δ = 77.16 ppm). ^{1}H data were recorded as follows: multiplicity (s = singlet, d = doublet, t = triplet, m = multiplet or unresolved, coupling constant(s) in Hz, integration). IR spectra were recorded on a Perkin–Elmer 983, Digital FT–IR spectrometer or Bruker–Tensor 27; frequencies are given in reciprocal centimeters (cm^{-1}) and only selected absorbance is reported. Mass spectra were determined on an Agilent 5973N MSD (EI) and Shimadzu LC–MS–2010EV (ESI) mass spectrometer or Agilent G6100 LC/MSD (ESI) single Quand mass spectrometer. High resolution mass spectra were recorded on Waters Micromass GCT Premier (EI) and Bruker Daltonics, Inc. APEXIII 7.0 TESLA FTMS (ESI) mass spectrometers.

II. Experimental:

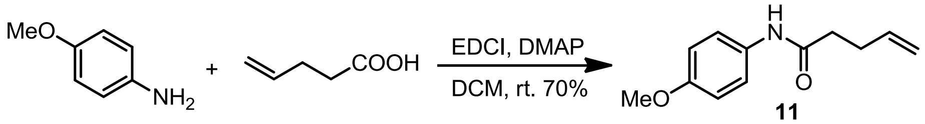

To a solution of 1-(3-dimethylaminopropyl)-3-ethylcarbodiimide hydrochloride (EDCI, 1.48 g, 7.7 mmol) and 4-dimethylaminopyridine (DMAP, 1.02 g, 8.4 mmol) in CH_2Cl_2 (14 mL) was added 4-pentenoic acid (0.80 g, 7.0 mmol) and 4-anisidine (0.86 g, 7.0 mmol) at room temperature. The resulting reaction mixture was stirred at room temperature for 5 h. The reaction mixture was diluted with CH_2Cl_2 (15 mL) prior to washing with HCl (aq, 1 N, 5 mL). The organic layer was separated, and the aqueous layer was extracted with CH_2Cl_2 (3 × 15 mL). The combined organic layers were washed with brine, dried over anhydrous Na_2SO_4, filtered, and concentrated under reduced pressure. The residue was subjected to purification on silica gel flash chromatography to give a yellow solid, which was further recrystallized in hexanes−ether to afford *N*-(4-methoxyphenyl)pent-4-enamide (**11**) as a white solid (1.0 g, 70% yield).
^{1}H NMR (400 MHz, $CDCl_3$): δ ppm 7.39 (d, *J* = 9.2 Hz, 2H), 7.21 (s, 1H), 6.84 (d, *J* = 8.8 Hz, 2H), 5.85–5.91 (m, 1H), 5.04–5.14 (m, 2H), 3.78 (s, 3H), 2.40–2.49 (m, 4H);
EI–MS: 205 (47), 123 (100), 122 (16), 108 (71), 95 (13), 55 (20).

N-(4-methylphenyl)pent-4-enamide (**18a**) [1].

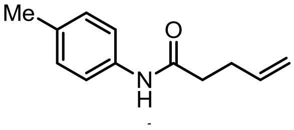

^{1}H NMR (400 MHz, $CDCl_3$): δ ppm 7.38 (d, *J* = 8.0 Hz, 2H), 7.12 (d, *J* = 8.0 Hz; 3H), 5.86–5.92 (m, 1H), 5.04–5.15 (m, 2H), 2.43–2.50 (m, 4H), 2.31 (s, 3H); EI–MS: 189 (26), 108 (11), 107 (100), 106 (37), 77 (10), 55 (14).

N-phenylpent-4-enamide (**18b**) [1]

^{1}H NMR (400 MHz, $CDCl_3$): δ ppm 7.63 (s, 1H), 7.51 (d, J = 8.0 Hz, 2H), 7.26–7.31 (m, 2H), 7.07–7.11 (m, 2H), 5.82–5.90 (m, 1H), 5.03–5.13 (m, 2H), 2.43–2.45 (m, 4H); ESI–MS: $[2M + Na]^+$ 373.0.

N-(4-chlorophenyl)pent-4-enamide (**18c**) [2]

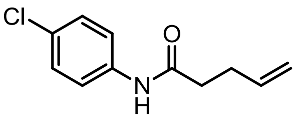

^{1}H NMR (400 MHz, $CDCl_3$): δ ppm 7.45 (d, J = 8.8 Hz, 2H), 7.27 (d, J = 7.2 Hz, 3H); 5.82–5.91 (m, 1H), 5.05–5.14 (m, 2H), 2.43–2.50 (m, 4H); EI–MS: 209 (18), 129 (33), 128 (9), 127 (100), 123 (9), 55 (30).

Synthesis of 4-methyl-*N*-*p*-tolylpent-4-enamide (**18d**) [3,4]

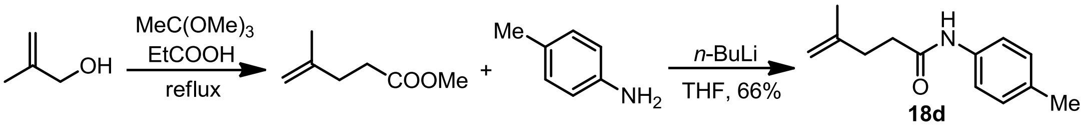

A solution of 2-methylprop-2-en-1-ol (4.0 g, 55 mmol) and propanoic acid (0.3 g, 4 mmol) in triethyl orthoacetate (25 g, 153 mmol) was heated with stirring to 125 °C until ethanol was no longer distilled from the reaction mixture. The solution was cooled to room temperature and Et_2O was added, then washed successively with 1 M aqueous HCl (2 × 10 mL), saturated aqueous NaHCO3 solution (2 × 10 mL), and brine (10 mL). The organic layers were dried over anhydrous Na_2SO_4, filtered, and concentrated under reduced pressure. The residue was subjected to purification on silica gel chromatography to give the ester as a colorless oil in 75% yield.

n-BuLi (1.6 M in hexane, 25 mL) was added dropwise to a solution of *p*-toluidine (2.14 g, 20 mmol) in THF (100 mL) at 0 °C under Ar. The reaction was warmed to room temperature and afterward stirred for 1 h, then the solution was cooled to −78 °C, and methyl 4-methylpent-4-enoate was slowly added and stirred for 1 h. Saturated NH_4Cl solution (10 mL) was added and the aqueous layer was extracted with Et_2O. The combined organic layers were washed with 0.5 M HCl (10 mL) and brine (10 mL), dried over anhydrous Na_2SO_4, filtered and concentrated under reduced pressure. The residue was subjected to purification on silica gel chromatography to give **18d** as white solid in 66% yield.

^{1}H NMR (400 MHz, $CDCl_3$): δ ppm 7.38 (d, J = 8.4 Hz, 2H), 7.20 (s, 1H), 7.11 (d, J = 8.0 Hz, 2H), 4.79 (d, J = 15.2 Hz, 2H), 2.42–2.52 (m, 4H), 2.31 (s, 3H), 1.78 (s, 3H); ^{13}C NMR (100 MHz, $CDCl_3$): δ ppm 170.98, 144.54, 135.52, 133.95, 129.53, 120.19, 110.84, 35.80, 33.30, 22.60, 20.93; FT–IR (film): ν (cm^{-1}) 3295, 2922, 1660, 1605, 1536, 1515, 1404, 1318, 891, 821; ESI-MS: $[M + Na]^+$ 226.1; HRMS (ESI) *m/z*: calculated for $C_{13}H_{17}NONa$: $[M + Na]^+$ 226.1200, found 226.1205.

2-methyl-*N*-*p*-tolylpent-4-enamide (**18e**) was prepared according to a procedure similar to the preparation of **11**, in 80% yield.

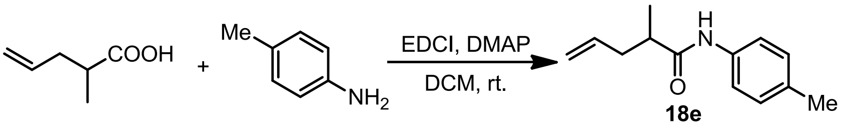

^{1}H NMR (400 MHz, $CDCl_3$): δ ppm 7.39 (d, *J* = 8.0 Hz, 3H), 7.09 (*J* = 8.0 Hz, 2H), 5.75–5.83 (m, 1H), 5.03–5.12 (m, 2H), 2.36–2.51 (m, 2H), 2.30 (s, 3H), 2.18–2.25 (m, 1H), 1.23 (d, *J* = 6.8 Hz, 3H); ^{13}C NMR (100 MHz, $CDCl_3$): δ ppm 174.24, 135.82, 135.50, 133.94, 129.51, 120.23, 117.21, 42.17, 38.52, 20.93, 17.54; FT–IR (film): ν(cm^{-1}) 3296, 2973, 1659, 1603, 1535, 1514, 1310, 1249, 915, 816; ESI-MS: $[M + Na]^+$ 226.0; HRMS (ESI) *m/z* calculated for $C_{13}H_{17}NONa$: $[M + Na]^+$ 226.1202, found 226.1198.

5-(4-Methoxyphenylamino)pentane-1,2-diol (**revised 13**) was synthesized according to Tellitu's procedure [5]

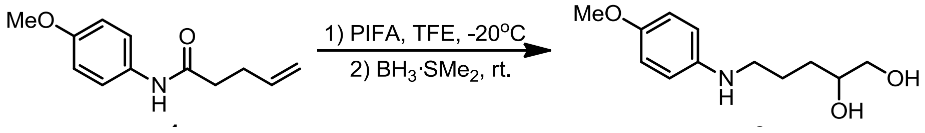

To a solution of *N*-(4-methoxyphenyl)pent-4-enamide (**11**) (139 mg, 0.68 mmol) in 2,2,2-trifluoroethanol (TFE) was added a solution of [bis(trifluoroacetoxy)iodo]benzene (PIFA) (438 mg, 1.02 mmol) in 2,2,2-trifluoroethanol (TFE) (10 mL) at –20 °C, and the mixture was stirred for 80 min. The reaction was quenched with aqueous Na_2CO_3 solution (10%, 10 mL). The aqueous phase was extracted with CH_2Cl_2 (3 × 15 mL), and the combined organic layers were washed with brine. The solution was dried over anhydrous Na_2SO_4 and concentrated under reduced pressure. The resulting residue was dissolved in THF (4 mL) and cooled to 0 °C, and $BH_3·SMe_2$ (3.4 mL, 2 M in THF, 6.8 mmol) was added dropwise. The reaction mixture was stirred at room temperature for 12 h, before CH_3OH (5 mL) was added and stirred for 15 min, then concentrated. The residue was purified by column chromatography to afford 5-(4-methoxyphenylamino)pentane-1,2-diol (**13**) as a brown oil (110 mg, 79% yield). ^{1}H NMR (400 MHz, $CDCl_3$): δ ppm 6.76 (d, *J* = 8.8 Hz, 2H), 6.58 (d, *J* = 8.8 Hz, 2H), 3.72 (s, 3H), 3.66 (m, 4H), 3.55–3.59 (m, 1H), 3.37–3.42 (m, 1H), 3.01–3.07 (m, 2H), 1.61–1.71 (m, 2H), 1.44–1.51 (m, 2H); ^{13}C NMR (100 MHz, $CDCl_3$): δ ppm 152.60, 142.45, 115.02, 114.91, 72.11, 66.85, 55.91, 45.45, 31.04, 26.03; FT–IR (film): ν(cm^{-1}) 3371, 2935, 2865, 2830, 1514, 1235, 1179, 1108, 1036, 821. ESI-MS: $[M + Na]^+$ 248.0; HRMS (ESI) *m/z* calculated for $C_{12}H_{20}NO_3$ $[M + H]^+$ 226.1438, found 226.1433;

4,5-Dihydroxy-*N*-(4-methoxyphenyl)pentanamide (**revised 12**) was synthesized according to a procedure similar to the preparation of 5-(4-methoxyphenylamino)pentane-1,2-diol

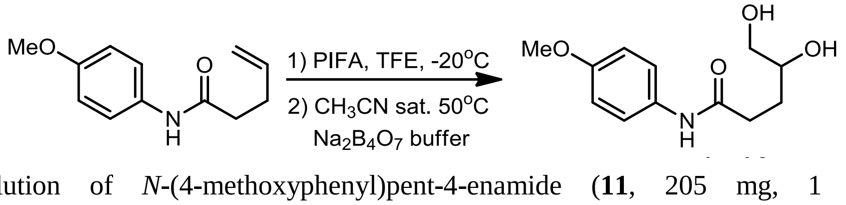

To a solution of *N*-(4-methoxyphenyl)pent-4-enamide (**11**, 205 mg, 1 mmol) in 2,2,2-trifluoroethanol (TFE, 10 mL) was added a solution of PIFA (470 mg, 1.1 mmol) in TFE (11 mL) at –20 °C, the mixture was stirred for 1.5 h, and the reaction was quenched with aqueous sodium carbonate solution (10%, 5 mL). The organic phase was extracted with CH_2Cl_2 (3 × 30 mL). The combined organic layers were washed with brine and the solution was dried over anhydrous Na_2SO_4, filtered, and concentrated under reduced pressure. The residue was added to a mixture of acetonitrile (9 mL) and aqueous saturated $Na_2B_4O_7$ solution (12 mL) and stirred for 3

days at 50 °C. The reaction mixture was then neutralized by adding a solution of hydrochloride acid (1 M) and extracted with ethyl acetate (3 × 30 mL), and the combined organic layers were washed with brine. The solution was dried over anhydrous Na_2SO_4 and then concentrated under reduced pressure. The residue was subjected to purification on silica gel chromatography to provide revised **12** as a white solid (1.86 g, 82% yield).
^{1}H NMR (400 MHz, $(CD_3)_2CO$): δ ppm 9.04 (s, 1H), 7.57 (d, *J* = 8.8 Hz, 2H), 6.85 (d, *J* = 9.2 Hz, 2H), 3.89–3.90 (m, 1H), 3.76 (s, 3H), 3.65–3.67 (m, 2H), 3.43–3.50 (m, 2H), 2.44–2.54 (m, 2H), 1.87–1.91 (m, 1H), 1.67–1.72 (m, 1H); ^{13}C NMR (100 MHz, $(CD_3)_2SO$): δ ppm 170.98, 154.92, 132.56, 120.53, 113.70, 70.63, 65.76, 55.09, 32.61, 29.30; FT–IR (film): ν (cm^{-1}) 3335, 2910, 1656, 1548, 1508, 1408, 1245, 1171, 1031, 836. EI–MS: *m/z* 239 (15), 221 (34), 124 (15), 123 (100), 122 (15), 108 (63); HRMS (EI) *m/z* calculated for $C_{12}H_{17}NO_4$ (M^+) 239.1158, found 239.1160;

2-allylpent-4-enoic acid was prepared according to a known procedure [6]

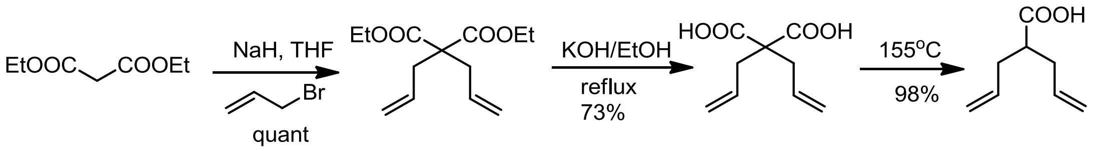

To the suspension of NaH (60% wt., 10 g, 250 mmol) in THF (300 mL) was added diethylmalonate (16 g, 15.2 mL, 100 mmol) dropwise in THF (100 mL) under Ar at 0 °C. The reaction mixture was warmed to rt and stirred for 15 min and then treated with allyl bromide (24.2 g, 17.3 mL, 200 mmol) and heated under reflux overnight. The reaction was quenched with NH_4Cl and extracted with Et_2O. The combined organic layers were washed with brine, dried over anhydrous Na_2SO_4, and concentrated *in vacuo* to give diethyl 2,2-diallylmalonate 24 g (nearly quant) as a yellow orange liquid. The crude yellow solid was used in the next step without further purification. The crude diethyl 2,2-diallylmalonate (24 g, 100 mmol) was dissolved in EtOH (45 mL), H_2O (21 mL), and KOH (14 g, 82.4 mmol) and heated under reflux for 3 h. The solvent was removed under reduced pressure, the resultant residue was taken up in H_2O, acidified to pH = 1 with 6 N HCl, and extracted with Et_2O, the combined organic layers were washed with brine, dried over anhydrous Na_2SO_4, and concentrated *in vacuo* to give the 2,2-diallylmalonic acid, and after recrystallization in EtOAc/petroleum ether a white solid (13.48 g, 73%) could be obtained. The crude 2,2-diallylmalonic acid (3.68 g, 20mmol) was heated at 155 °C for about 5 h to afford 2-allylpent-4-enoic acid (2.74 g, 98%) as a dark amber liquid.

N-([1,1'-biphenyl]-2-yl)-2-allylpent-4-enamide (**14**) was prepared according to a procedure similar to the preparation of **11**.

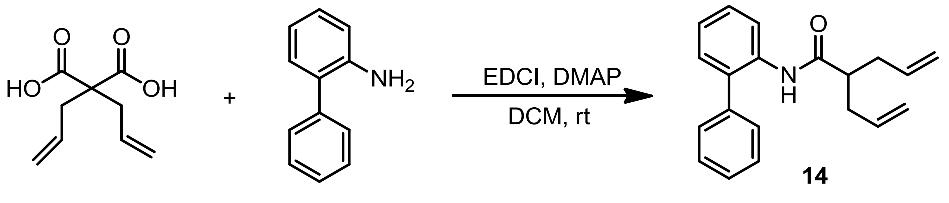

14

^{1}H NMR (400 MHz, CDCl3): δ ppm 8.28–8.26 (d, J = 8.4 Hz, 1H), 7.50–7.42 (m, 3H), 7.38–7.34(m, 3H), 7.25–7.23 (m, 1H), 7.19–7.12 (m, 2H), 5.72–5.66 (m, 2H), 5.04–4.97 (m, 4H), 2.38–2.32 (m, 2H), 2.22–2.13 (m, 3H); ^{13}C NMR (100 MHz, $CDCl_3$): δ ppm 172.53, 138.16,

135.28, 134.59, 132.32, 129.99, 129.46 129.02, 128.42, 128.03, 124.33, 121.76, 117.29, 48.40, 36.46; FT–IR (film): ν(cm–1): 3249, 2902, 1655, 1522, 1434, 1276, 998, 915, 755,700. ESI-MS: $[M + Na]^+$ 314.1; HRMS (ESI) m/z calculated for $C_{20}H_{22}NO$: $[M + H]^+$ 292.1696, found 292.1691.

(5-([1,1'-Biphenyl]-2-ylimino)-4-allyltetrahydrofuran-2-yl)methanol (**C**) was synthesized according to a procedure similar to the preparation of **revised 13**.

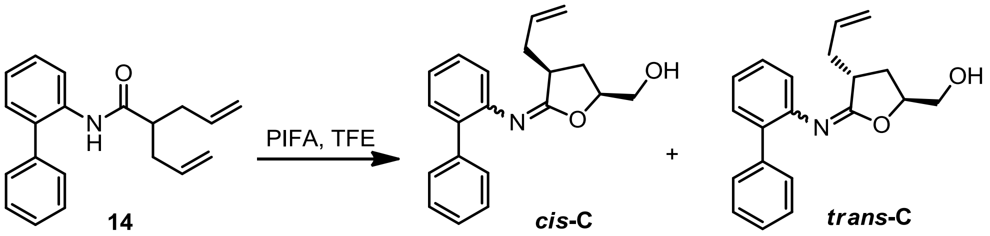

To a solution of compound **14** (583 mg, 2.0 mmol) in TFE (20 mL) was added a solution of PIFA (946 mg, 2.2 mmol) in TFE (22 mL) at –20 °C and stirred for 2.5 h. The reaction was quenched with Na_2CO_3 (aq, 10%, 10 mL), the aqueous phase was extracted with CH_2Cl_2 (3 × 15 mL), the combined organic layers were washed with brine. The solution was dried over anhydrous Na_2SO_4 and concentrated under reduced pressure. From the crude ^{1}H NMR, the ratio of *anti syn* was about 1.1:1. The residue was subjected to purification on silica gel chromatography to give each isomer.

***cis*-iminolactone C** :^{1}H NMR (400 MHz, $CDCl_3$): 7.48–7.45(m, 2H), 7.40–7.36 (t, J = 7.2 Hz, 2H), 7.31–7.25 (m, 3H), 7.14–7.11 (t, J = 7.2, 1H), 6.98–6.96 (d, J = 8.0 Hz, 1H), 5.78–5.72 (m, 1H), 5.10–5.02 (m, 2H), 4.17–4.11 (m, 1H), 3.53–3.50 (d, J = 12.8 Hz, 1H), 3.11–3.08 (d, J = 12.4 Hz, 1H), 2.81–2.74 (m, 2H), 2.13–2.00 (m, 2H), 1.47–1.39 (m, 1H); ^{13}C NMR (100 MHz, $CDCl_3$): δ ppm 163.03, 145.55, 141.06, 135.68, 133.30, 130.14, 128.84, 128.05, 126.88, 123.78, 122.15, 116.81, 81.09, 63.24, 40.90, 36.01, 29.59; FT–IR (film): ν(cm^{-1}): 3367, 2925, 1698, 1475, 1434, 1189, 1009, 920, 743, 702. ESI-MS: $[M + Na]^+$ 330.2; HRMS (ESI) *m/z* calculated for $C_{20}H_{21}NO_2Na[M + Na]^+$ 330.1465 , found 330.1458.

***trans*-iminolactone C** :^{1}H NMR (400 MHz, $CDCl_3$): 7.52–7.50 (m, 2H), 7.41–7.37 (t, J = 7.2, 2H), 7.33–7.26 (m, 3H), 7.16–7.11 (t, J = 7.2Hz, 1H), 6.99–6.97 (d, J = 7.6 Hz, 1H), 5.79–5.73 (m, 1H), 5.12–5.05 (m, 2H), 4.23–4.20 (m, 1H), 3.41–3.38(d, J = 12.0 Hz, 1H), 3.13–3.10 (d, J = 12.8 Hz, 1H), 2.80–2.77 (m, 1H), 2.62–2.57 (m, 1H), 2.25–2.19 (m, 1H), 2.00 –1.93 (m, 1H), 1.80–1.73 (m, 1H); ^{13}C NMR (100 MHz, $CDCl_3$): δ ppm 164.00, 145.58, 141.32, 135.23, 133.27, 130.09, 128.87, 128.04, 126.72, 123.77, 122.20, 117.27, 80.98, 64.53, 39.98, 36.82, 29.34; FT–IR (film): ν(cm^{-1}): 3354, 2926, 1694, 1474, 1432, 1191, 1048, 918, 742, 701. ESI-MS: $[M + Na]^+$ 330.2; HRMS (ESI) *m/z* calculated for $C_{20}H_{21}NO_2Na$ $[M + Na]^+$ 330.1465 , found 330.1462.

N-(3-allyl-5-((benzyloxy)methyl)dihydrofuran-2(3*H*)-ylidene)-[1,1'-biphenyl]-2-amine (**benzylated iminolactone C**) was synthesized according to a known procedure [7]

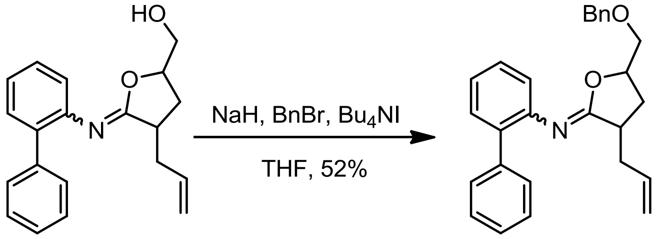

NaH (80% wt, 0.407 mmol, 12.2 mg) was added to the solution of **C** (a mixture with a ratio of about *syn*:*anti* = 4:1, 0.407 mmol, 126 mg) in dry THF (3 mL) at room temperature under N_2, and stirred for 30 min, then *n*-Bu_4NI (0.02 mmol, 8.0 mg) and BnBr (0.407 mmol, 48 μL) were added, and the reaction was stirred overnight. The reaction was quenched with NH_4Cl and extracted with DCM, and the combined organic layers were washed with brine, dried over anhydrous Na_2SO_4, filtered, and concentrated under reduced pressure. The residue was subjected to purification by silica gel chromatography to give a yellow oil (*anti*:*syn* = 4:1, 83.7 mg, 52% yield). ^{1}H NMR (400 MHz, $CDCl_3$): 7.44–7.39 (m, 2H), 7.34–7.21 (m, 10H), 7.12–7.08 (t, *J* = 7.6 Hz, 1H), 6.99–6.97 (d, *J* = 7.6Hz, 1H), 5.75–5.62 (m, 1H), 5.06–4.99 (m, 2H), 4.44 (s, 2H), 4.35–4.30 (m, 0.2H), 4.27–4.21 (m, 0,8H), 3.26–3.25 (d, *J* = 4.8 Hz, 2H), 2.87–2.81 (m, 0.2 H), 2.77–2.63 (m, 1.6H), 2.57–2.50 (m, 0.2H), 2.20–2.11 (m, 1H), 2.09–2.00 (m, 1H), 1.81–1.74 (m, 0.2H), 1.40–1.32 (m, 0.8H).

3-(5-((Benzyloxy)methyl)-2-oxotetrahydrofuran-3-yl)prop-1-en (**17**) was prepared from hydrolysis of the benzylated iminolactone C according to a known procedure [8]

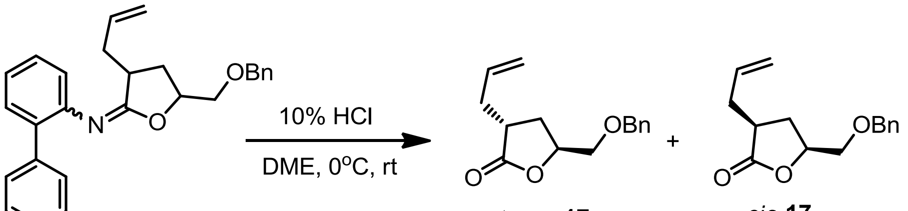

trans-**17** *cis*-**17**

10% HCl (0.71 mL) was slowly added to the solution of ***O*-benzylated C** (83.7mg, 0.21mmol) in dimethoxy ethane (DME)(3mL) at room temperature. The reaction was continuously stirred overnight. Then the reaction was diluted with H_2O (10 mL) and extracted with Et_2O (3 × 7 mL), and the combined organic layers were washed with saturated NH_4Cl solution and brine, dried over anhydrous Na_2SO_4, and filtered 4:1. The residue was subjected to purification on silica gel chromatography to give each isomer (34% yield).

***cis*-17:** ^{1}H NMR (400 MHz, $CDCl_3$): δ ppm 7.37–7.28 (m, 5H), 5.80–5.73 (m, 1H), 5.12–5.07 (m, 2H), 4.59 (s, 2H), 4.57–4.54 (m, 1H), 3.69–3.65 (dd, *J* = 3.6 Hz, 11.2Hz, 1H), 3.61–3.57 (dd, *J* = 5.2Hz, 11.2Hz, 1H), 2.75–2.71 (m, 1H), 2.66–2.61 (m, 1H), 2.39–2.32 (m, 1H), 2.30–2.24 (m, 1H), 1.84–1.75 (m, 1H); ^{13}C NMR (100 MHz, $CDCl_3$): δ ppm 177.91, 137.72, 134.61, 128.48, 127.85, 127.74, 117.56, 77.43, 73.62, 71.21, 39.96, 34.42, 30.06; FT–IR (film): ν (cm^{-1}): 2924, 2863, 1771, 1454, 1360, 1171, 1121, 923, 740, 699. ESI-MS: $[M + Na]^+$ 269.1; HRMS (ESI) *m/z* calculated for $C_{15}H_{18}O_3Na$ $[M + Na]^+$ 269.1148, found 269.1148;

***trans*-17:**1H NMR (400 MHz, CDCl3): δ ppm 7.37–7.29 (m, 5H), 5.80–5.73 (m, 1H), 5.14–5.09 (m, 2H), 4,63–4.55 (m, 3H), 3.68–3.64 (dd, *J* = 3.6Hz, 10.8 Hz, 1H), 3.58–3.54 (dd, *J* = 4.0 Hz, 10.8 Hz, 1H), 2.88–2.85 (m, 1H), 2.59–2.55 (m, 1H), 2.31–2.24 (m, 2H), 2.10–2.02 (m, 1H); 13C NMR (100 MHz, CDCl3): δ ppm 178.89, 137.68, 134.49, 128.52, 127.87, 127.63, 117.75, 76.94, 73.66, 71.75, 38.92, 35.11, 29.68; FT–IR (film): ν (cm–1): 2921, 2850, 1766, 1454, 1360, 1261, 1097, 1024, 800, 698. ESI-MS: [M + Na]+ 269.1; HRMS (ESI) *m/z* calculated for C15H18O3Na [M + Na]+ 269.1148, found 269.1151;

(*S*)-5-(Hydroxymethyl)dihydrofuran-2(3*H*)-one (**15**) was prepared according to a known procedure [9]

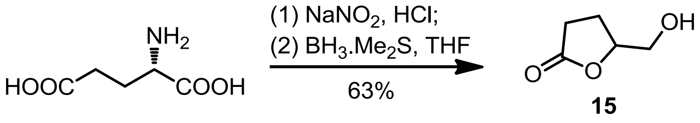

To a solution of L-glutamic acid (10.0 g, 68.0 mmol) in water (68 mL) and 2 N HCl (40 mL) at 0 °C was added dropwise a solution of sodium nitrate (5.6 g, 81.5 mmol) in water (40 mL) over 2 h. After the addition was complete, the reaction mixture was allowed to warm to room temperature and stirred for 16 h. The aqueous phase was saturated with solid NaCl and was extracted with ethyl acetate (3 × 100 mL). The combined organic layers were dried with anhydrous Na2SO4, filtered, and concentrated under reduced pressure. The crude yellow solid was used in the next step without further purification. To the solution of (*S*)-5-oxotetrahydrofuran-2-carboxylic acid (1.404 g, 10.8mmol) in tetrahydrofuran (10 mL) was added BH3 .Me2S (2 M in THF, 6.5 mL, 13mmol) over 1 h. The resulting mixture was stirred at room temperature for 4 h. The reaction was quenched with methanol (7 mL), and the resulting mixture was concentrated in vacuo. The residue was purified by flash chromatography to afford (S)-5-(hydroxymethyl)dihydrofuran-2(3*H*)-one (**15**) as a colorless oil (0.783 g, 63%). 1H NMR (300 MHz, CDCl3) δ ppm 4.68–4.61 (m, 1H), 3.94–3.89 (m, 1H), 3.70–3.62 (m, 1H), 2.85 (br, 1H), 2.70–2.49 (m, 2H), 2.34–2.06 (m, 2H).

(*S*)-5-((Benzyloxy)methyl)dihydrofuran-2(3*H*)-one (**16**)

A round bottom flask, equipped with a magnetic stirrer bar, was charged with sodium hydride (60%, 0.0816 g, 2.04 mmol) and DMF (3 mL) and cooled with an ice-bath under argon. (S)-5-(Hydroxymethyl)dihydrofuran-2(3*H*)-one (0.232g, 2 mmol) was dissolved in DMF (1 mL) added dropwise to the suspension above. After stirring for 1.5 h between 0 °C and rt, the mixture was recooled to 0 °C and rapidly treated with BnBr (0.26 mL, 2.2 mmol), and the resulting white suspension was stirred overnight at rt. The mixture was partitioned between water and 1:1 Et2O:hexane. The combined organic extracts were washed with brine (3 × 5 mL) and dried over anhydrous Na2SO4, filtered, and concentrated under reduced pressure. The residue was subjected to purification on silica gel chromatography to give (*S*)-5-((benzyloxy)methyl)dihydrofuran-2(3*H*) -one (**16**) as a yellow oil (0.120 g, 29% yield). 1H NMR (400 MHz, CDCl3) δ ppm 7.37–7.28 (m, 5 H), 4.70–4.64 (m, 1 H), 4.57–4.56 (d, J = 3.2 Hz, 2 H), 3.70–3.67 (dd, J = 3.2, 10.8, 1 H), 3.60–3.57 (dd, J = 4.0, 10.8 Hz, 1 H), 2.66–2.58 (m, 1 H), 2.52–2.44 (m, 1H), 2.33–2.24 (m, 1 H), 2.17–2.08 (m, 1 H).

Compound **17** from the allylation of lactone.

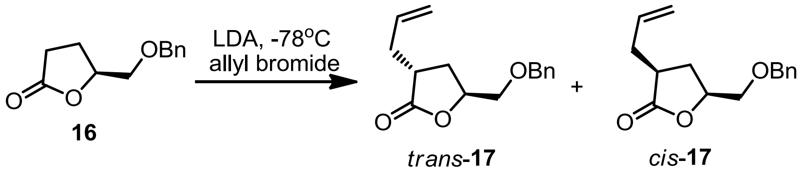

To a stirred solution of LDA (1.8 M in THF) in dry THF was added (*S*)-5-((benzyloxy)methyl)dihydrofuran-2(3*H*)-one (60 mg, 0.291 mmol) dropwise over 2 min at –78 ºC, and the resulting solution was stirred for 1 h. Allylbromide (25.2 μL, 0.291 mmol) was added dropwise slowly, and the resulting mixture was stirred for 1 h at –78 °C. Then the reaction was warmed to –50 °C and quenched with a solution of $NaHCO_3$ (sat. aq, 1 mL). The reaction was then warmed to rt, and extracted with Et_2O (3 × 5 mL). The combined organic layers were dried over Na_2SO_4, the resulting mixture was concentrated *in vacuo* and purified by flash chromatography to give a mixture of the two diastereoisomers, from the crude ^{1}H NMR, *anti:syn* = 5:1.

***cis*-17:** ^{1}H NMR (400 MHz, $CDCl_3$): δ ppm 7.37–7.28 (m, 5 H), 5.80–5.73 (m, 1 H), 5.12–5.07 (m, 2 H), 4.59 (s, 2 H), 4.57–4.54 (m, 1 H), 3.69–3.65 (dd, *J* = 3.6 Hz, 11.2 Hz, 1 H), 3.61–3.57 (dd, *J* = 5.2 Hz, 11.2 Hz, 1H), 2.75–2.71 (m, 1 H), 2.66–2.61 (m, 1 H), 2.39–2.32 (m, 1 H), 2.30–2.20 (m, 1 H), 1.84–1.75 (m, 1H); ^{13}C NMR (100 MHz, $CDCl_3$): δ ppm 177.91, 137.72, 134.61, 128.48, 127.85, 127.74, 117.56, 77.43, 73.62, 71.21, 39.96, 34.42, 30.06; FT–IR (film): ν cm^{-1}: 2924, 2863, 1771, 1454, 1360, 1171, 1121, 923, 740, 699; ESI-MS: [M + Na]+ 269.1; HRMS (ESI) *m*/*z* calculated for $C_{15}H_{18}O_3Na$: $[M + Na]^+$ 269.1148, found 269.1148.

***trans*-17:** ^{1}H NMR (400 MHz, $CDCl_3$): δ ppm 7.37–7.29 (m, 5H), 5.80–5.73 (m, 1H), 5.14–5.09 (m, 2H), 4,63–4.55 (m, 3H), 3.68–3.64 (dd, *J* = 3.6Hz, 10.8 Hz, 1H), 3.58–3.54 (dd, *J* = 4.0 Hz, 10.8 Hz, 1H), 2.88–2.85 (m, 1H), 2.59–2.55 (m, 1H), 2.31–2.24 (m, 2H), 2.10–2.02 (m, 1H);
^{13}C NMR (100 MHz, $CDCl_3$): δ ppm 178.89, 137.68, 134.49, 128.52, 127.87, 127.63, 117.75, 76.94, 73.66, 71.75, 38.92, 35.11, 29.68; FT–IR (film): ν cm^{-1}: 2921, 2850, 1766, 1454, 1360, 1261, 1097, 1024, 800, 698. ESI-MS: $[M + Na]^+$ 269.1; HRMS (ESI) *m*/*z* calculated for $C_{15}H_{18}O_3Na$: $[M + Na]^+$ 269.1148, found 269.1151.

4,5-Dihydroxy-*N*-*p*-tolylpentanamide (**19a**)
^{1}H NMR (400 MHz, $(CD_3)_2CO$): δ ppm 9.18 (s, 1H), 7.54 (d, *J* = 8.0Hz, 2H), 7.08 (d, *J* = 8.0Hz, 2H), 4.06 (m, 1H), 3.85 (m, 1H), 3.66–3.64 (m, 1H), 3.53–3.43 (m, 2H), 2.56–2.46 (m, 2H), 2.26 (s, 3H), 1.94–1.86 (m, 1H), 1.75–1.68 (m, 1H); ^{13}C NMR (100 MHz, $(CD_3)_2SO$): δ ppm 171.33, 136.91, 131.69, 128.97, 119.05, 70.67, 65.82, 32.76, 29.31, 20.40; FT–IR (film): ν(cm^{-1}) 3286, 1655, 1601, 1541, 1418, 1406, 1111, 1066, 869, 815. ESI-MS: $[M + Na]^+$ 246.0; HRMS (ESI) *m/z* calculated for $C_{12}H_{17}NO_3Na$: $[M + Na]^+$ 246.1101, found 246.1096;

4,5-Dihydroxy-*N*-phenylpentanamide (**19b**)
^{1}H NMR (400 MHz, $(CD_3)_2CO$): δ ppm 9.23 (s, 1H), 7.70 (d, *J* = 8.0 Hz, 2H), 7.32 (t, *J* = 7.8 Hz, 2H), 7.07 (t, *J* = 7.2 Hz, 1H), 3.97 (d, *J* = 4.4 Hz, 1H), 3.78 (t, *J* = 5.8 Hz, 1H), 3.68–3.73 (m, 1H),

3.47–3.57 (m, 2H), 2.52–2.62 (m, 2H), 1.93–1.97 (m, 1H), 1.73–1.78 (m, 1H); ^{13}C NMR (100 MHz, $(CD_3)_2CO$): δ ppm 171.57, 139.40, 128.60, 122.85, 119.03, 70.65, 65.82, 32.80, 29.26.

N-(4-chlorophenyl)-4,5-dihydroxypentanamide (**19c**)
^{1}H NMR (400 MHz, $(CD_3)_2CO$): δ ppm 9.31 (s, 1H), 7.72 (d, *J* = 8.8 Hz, 2H), 7.33 (d, *J* = 8.8 Hz, 2H), 3.81 (d, *J* = 4.8 Hz, 1H), 3.63–3.68 (m, 2H), 3.44–3.53 (m, 2H), 2.51–2.58 (m, 2H), 1.90–1.93 (m, 1H), 1.67–1.75 (m, 1H); ^{13}C NMR (100 MHz, $(CD_3)_2CO$): δ ppm 172.67, 139.52, 129.51, 128.33, 121.62, 121.53, 72.26, 67.28, 34.07, one peak is covered by the peak of $(CD_3)_2CO$; FT–IR (film): ν(cm^{-1}) 3263, 1668, 1609, 1596, 1551, 1492, 1312, 1102, 1034, 832. ESI-MS: [M + Na]$^+$ 266.0; HRMS (ESI) *m/z* calculated for $C_{11}H_{14}ClNO_3Na$: [M + Na]$^+$ 266.0554, found 266.0560.

4,5-Dihydroxy-4-methyl-*N*-*p*-tolylpentanamide (**19d**)
^{1}H NMR (400 MHz, $(CD_3)_2CO$): δ ppm 9.16 (s, 1H), 7.57 (d, *J* = 8.0 Hz, 2H), 7.12 (d, *J* = 8.0 Hz, 2H), 3.86 (t, *J* = 6.0 Hz, 1H), 3.65 (s, 1H), 3.40 (d, *J* = 5.6 Hz, 2H), 2.51–2.58 (m, 2H), 2.30 (s, 3H), 1.92–1.99 (m, 1H), 1.81–1.86 (m, 1H), 1.17 (s, 3H); ^{13}C NMR (100 MHz, $(CD_3)_2CO$): δ ppm 172.98, 138.12, 133.36, 130.01, 120.30, 72.40, 70.35, 34.54, 32.33, 24.58, 20.90; FT–IR (film): ν(cm^{-1}) 3232, 2925, 1654, 1610, 1557, 1514, 1313, 1260, 1062, 822. ESI-MS: [M + Na]$^+$ 260.0; HRMS (ESI) *m/z* calculated for $C_{13}H_{19}NO_3Na$: [M + Na]$^+$ 260.1257; found: 260.1256;

4,5-Dihydroxy-2-methyl-*N*-*p*-tolylpentanamide (**19e**)
^{1}H NMR (400 MHz, $(CD_3)_2CO$): δ ppm 9.21–9.23 (m, 2 × 1H), 7.55–7.57 (m, 2 × 2H), 7.07–7.08 (m, 2 × 2H), 4.08–4.19 (m, 2 × 2H), 3.66–3.74 (m, 1H), 3.44–3.52 (m, 2H), 2.83–2.85 (m, 1H), 2.72–2.76 (m, 1H), 2.25 (s, 2 × 3H), 1.81–1.92 (m, 2 × 1H), 1.56–1.62 (m, 1H), 1.40–1.46 (m, 1H), 1.17–1.20 (m, 2 × 3H); ^{13}C NMR (100 MHz, $(CD_3)_2CO$): δ ppm 176.16, 175.81, 137.78, 137.64, 133.36, 133.26, 129.79, 120.45, 120.43, 70.88, 70.61, 67.51, 67.36, 38.81, 38.63, 38.40, 38.22, 20.78, 19.19, 17.92; FT–IR (film): ν(cm^{-1}) 3305, 2929, 1662, 1603, 1538, 1515, 1310, 1101, 1055, 816. ESI-MS: [M + Na]$^+$ 260.0; HRMS (ESI) *m/z* calculated for $C_{13}H_{19}NO_3Na$: [M + Na]$^+$ 260.1257; found: 260.1256.

2-Allyl-*N*-*p*-tolylpent-4-enamide (**20**) was synthesized according to a procedure similar to the preparation of *N*-(4-methoxyphenyl)pent-4-enamide (**11**) by using 2-allylpent-4-enoic acid and *p*-toluidine as starting materials, with 74% yield.

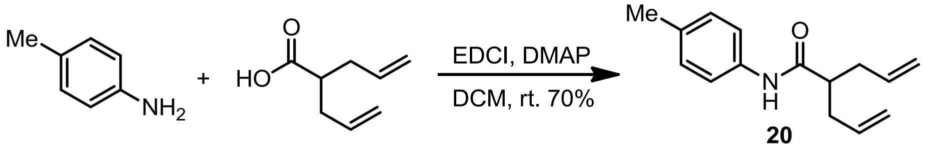

^{1}H NMR (400 MHz, $CDCl_3$): δ ppm 7.37 (d, *J* = 8.4 Hz, 2H), 7.16 (s, 1H), 7.11 (d, *J* = 8.0 Hz, 2H), 5.78–5.85 (m, 2H), 5.05–5.14 (m, 4H), 2.45–2.48 (m, 2H), 2.26–2.33 (m, 6H); ^{13}C NMR (100 MHz, $CDCl_3$): δ ppm 172.74, 135.65, 135.27, 134.13, 129.58, 120.28, 117.46, 48.25, 36.68, 20.97;
FT–IR (film): ν(cm^{-1}) 3297, 2931, 1656, 1597, 1525, 1406, 1308, 1254, 913, 815. EI–MS: *m/z* 108 (10), 107 (100), 106 (28), 77 (9), 67 (11), 41 (12); HRMS (EI) *m/z* calculated for *m/z* calculated for $C_{15}H_{19}NO[M]^+$229.1467; found: 229.1466.

2-(2,3-Dihydroxypropyl)-*N*-(*p*-tolyl)pent-4-enamide (**21**) was synthesized according to a procedure similar to the preparation of revised **12** with 80% yield.

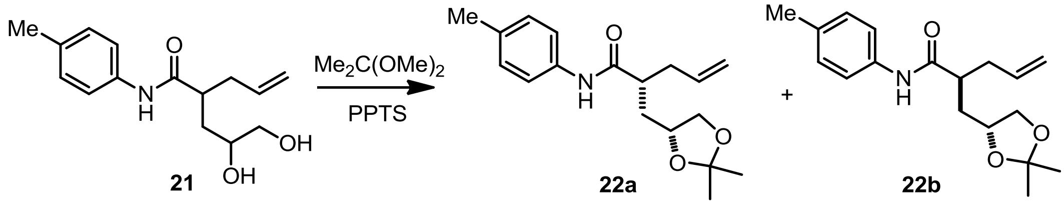

To a solution of 2-(2,3-dihydroxypropyl)-*N*-(*p*-tolyl)pent-4-enamide (two isomers, 85 mg, 0.32 mmol) in DMF (4 mL) was added 2,2-dimethoxypropane (50 mg, 0.48 mmol) and PPTS (8 mg, 0.032 mmol) at room temperature, and the mixture was stirred for 8 h. The mixture was extracted with CH_2Cl_2 (3 × 5 mL), the combined organic layers were washed with brine, dried over anhydrous Na_2SO_4, and concentrated under reduced pressure. The residue was subjected to purification on silica gel chromatography to give **22b** (33 mg) and **22a** (32 mg) with an overall yield of 67%.

22a: ^{1}H NMR (400 MHz, $CDCl_3$): δ ppm 7.62 (s, 1H), 7.37 (d, *J* = 8.0 Hz, 2H), 7.11 (d, *J* = 8.4 Hz, 2H), 5.78–5.85 (m, 1H), 5.03–5.13 (m, 2H), 4.07–4.14 (m, 1H), 4.01 (t, *J* = 7.4 Hz, 1H), 3.51 (t, *J* = 7.4 Hz, 1H), 2.48–2.62 (m, 2H), 2.30 (s, 3H), 2.20–2.26 (m, 1H), 1.96–2.04 (m, 1H), 1.73–1.80 (m, 1H), 1.41 (s, 3H), 1.35 (s, 3H); FT–IR (film): ν(cm^{-1}) 3297, 2984, 2933, 1660, 1603, 1532, 1514, 1246, 1060, 818. ESI-MS: $[M + Na]^+$ 326.1; HRMS (ESI) calculated for $C_{18}H_{25}NO_3Na$: $[M + Na]^+$ 326.1727; found: 326.1738;

22b: ^{1}H NMR (400 MHz, $CDCl_3$): δ ppm 7.54 (s, 1H), 7.37 (d, *J* = 8.0 Hz, 2H), 7.10 (d, *J* = 8.0 Hz, 2H), 5.78–5.85 (m, 1H), 5.05–5.15 (m, 2H), 4.19–4.20 (m, 1H), 4.07 (t, *J* = 7.0 Hz, 1H), 3.53 (t, *J* = 7.6 Hz, 1H), 2.45–2.52 (m, 2H), 2.31–2.36 (m, 4H), 1.96–2.04 (m, 1H), 1.73–1.80 (m, 1H), 1.41 (s, 3H), 1.35 (s, 3H); ^{13}C NMR (100 MHz, $CDCl_3$): δ ppm 173.00, 135.53, 133.97, 129.57, 120.12, 117.67, 109.28, 74.72, 69.56, 44.86, 36.91, 35.94, 27.09, 25.82, 20.96; FT–IR (film): ν(cm^{-1}) 3309, 2985, 2940, 2864, 1666, 1663, 1609, 1541, 1515, 827. EI–MS: m/z(%) 162 (21), 107 (100), 106 (25), 67 (15), 43 (63), 41 (19); HRMS (EI) *m/z* calculated for $C_{18}H_{25}NO_3$ $[M]^+$ 303.1834; found: 303.1833;

Compound **23** was synthesized according to a procedure similar to the preparation of **21**, with 87% yield.

^{1}H NMR (400 MHz, $CDCl_3$): δ ppm 8.35–8.40 (m, 2 × 1H), 7.37–7.42 (m, 2 × 2H), 7.03 (d, *J* = 8.0 Hz, 2 × 2H), 4.09–4.15 (m, 2 × 1H), 4.00–4.03 (m, 2 × 1H), 3.68–3.77 (m, 2 × 2H), 3.61–3.63 (m, 2 × 1H), 3.49–3.53 (m, 2 × 1H), 3.39–3.44 (m, 2 × 1H), 2.91–2.97 (m, 2 × 1H), 2.77–2.78 (m, 2 × 1H), 2.30 (s, 2 × 3H), 1.89–2.01 (m, 2 × 1H), 1.80–1.86 (m, 2 × 1H), 1.52–1.69 (m, 2 × 2H), 1.41–1.43 (m, 2 × 3H), 1.32–1.36 (m, 2 × 3H); ^{13}C NMR (100 MHz, $CDCl_3$): δ ppm 173.69, 135.38, 134.24, 129.64, 120.12, 120.05, 109.34, 74.05, 69.91, 69.95, 67.03, 41.18, 37.31, 36.16, 27.18, 25.84, 20.79; ESI-MS: $[M + Na]^+$ 360.0; HRMS (ESI) *m/z* calculated for $C_{18}H_{27}NO_5Na$: $[M + Na]^+$ 360.1781; found: 360.1788.

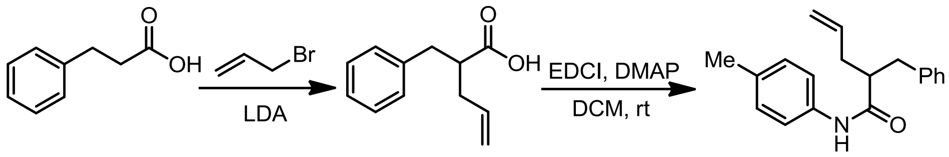

n-BuLi (13.1 mL, 1.6 M in hexane, 21 mmol) was added dropwise to the solution of iPr_2NH (2.95 mL, 21 mmol) in THF (15 mL) at 0 °C, the mixture was stirred for 30 min. Then, a solution of hydrocinnamic acid (1.5 g, 10 mmol) in THF (10 mL) was added dropwise over 20 min, and stirring was continued for another 30 min at the same temperature. Allyl bromide (0.45 mL, 10.45 mmol) was added, and the stirring was continued overnight. The solvent was removed under reduced pressure. The resulting residue was diluted with water (100 mL) and extracted with ether (50 mL). The pH of the separated aqueous layer was adjusted to 2 by HCl (3 M) and then extracted with ether (3 × 50 mL). The combined organic layers were washed with brine, dried over anhydrous Na_2SO_4, filtered, and concentrated under reduced pressure. The residue was subjected to purification on silica gel chromatography (eluent: hexanes:ethyl acetate = 2:1)to give 2-benzylpent-4-enoic acid (900 mg, 47% yield). ^{1}H NMR (400 MHz, $CDCl_3$): δ ppm 7.29–7.25 (m, 2H), 7.22–7.16 (m, 3H), 5.82–5.72 (m, 1H), 5.11–5.05 (m, 2H), 3.01–2.94 (m, 1H), 2.81–2,72 (m, 2H), 2.41–2.34 (m, 1H), 2.32–2.51(m, 1H).

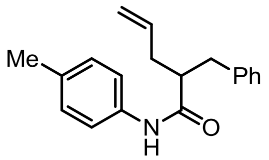

24a

^{1}H NMR (400 MHz, $CDCl_3$): δ ppm 7.28–7.24 (m, 2H), 7.21–7.16 (m, 5H), 7.06–7.04 (d, *J* = 8.4 Hz, 2H), 6.80 (br, 1H), 5.87–5.78 (m, 1H), 5.16–5.06 (m, 2H), 3.02–2.96 (dd, *J* = 9.6, 13.6 Hz, 1H), 2.86–2.81 (dd, *J* = 5.2, 13.6 Hz, 1H), 2.57–2.45 (m, 2H), 2.35–2.30 (m, 1H), 2.28 (s, 3H); ^{13}C NMR (100 MHz, $CDCl_3$): δ ppm 172.46, 139.66, 135.55, 134.93, 134.02, 129.36, 128.99, 128.60, 126.50, 120.38, 117.37, 50.85, 38.80, 36.81, 20.84; FT–IR (film): ν (cm^{-1}) : 3280, 2925, 1651, 1513, 1310, 1256, 991, 920, 820, 699. ESI-MS: $[M + Na]^+$ 302.2; HRMS (ESI) *m/z* calculated for $C_{19}H_{21}N_1Na_1O_1$ $[M + Na]^+$ 302.1515, found 302.1521.

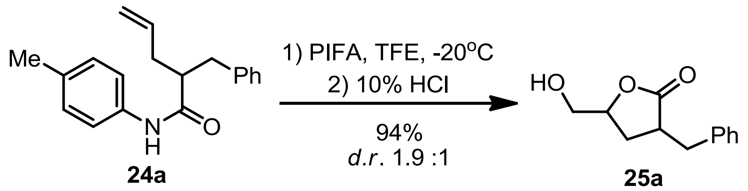

24a **25a**

To a solution of 2-benzyl-*N*-(*p*-tolyl)pent-4-enamide (**24a**) (139.5 mg, 0.5 mmol) in TFE (5 mL) was added a solution of PIFA (236.5 mg, 0.55 mmol) in TFE (5.5 mL) at –20 °C, and the mixture stirred for 2.5 h. The reaction was quenched with aqueous Na_2CO_3 solution (10%, 10 mL) and extracted with Et_2O, then 10% HCl was added to the organic layers, and the mixture was stirred overnight. The mixture was diluted with Et_2O, washed with saturated NH_4Cl and brine, dried over anhydrous Na_2SO_4, filtered, and concentrated under reduced pressure. The residue was subjected to purification by silica gel chromatography to give 20a (*anti*:*syn* = 1.9:1, 94% yield). ^{1}H NMR (400 MHz, $CDCl_3$): δ ppm 7.32–7.29 (t, *J* = 6.8 Hz, 2H), 7.26–7.22 (m, 1H), 7.20–7.19 (d, *J* = 7.2 Hz, 2H), 4.48–4.44 (m, 0.35 H), 4.43–4.38 (m, 0.64 H), 3.87–3.79 (m, 1H), 3.60–3.52 (m, H), 3.31–3.26 (dd, *J* = 4.0, 13.6 Hz, 0.35 H), 3.21–3.16 (dd, *J* = 4.8, 14.0 Hz, 0.65 H), 3.12–3.04 (m, 0.65 H), 2.99–2.95 (m, 0.35 H), 2.81–2.71 (m, 1H), 2.38 (br, 0.65H), 2.26 (br, 0.35H), 2.22–2.15 (m, 1H), 2.09–2.02 (m, 0.67H), 1.91–1.82 (m, 0.35H).

(E)-2-Allyl-N-(p-tolyl)hex-4-enamide (**24b**) was prepared according to the known procedure [10].

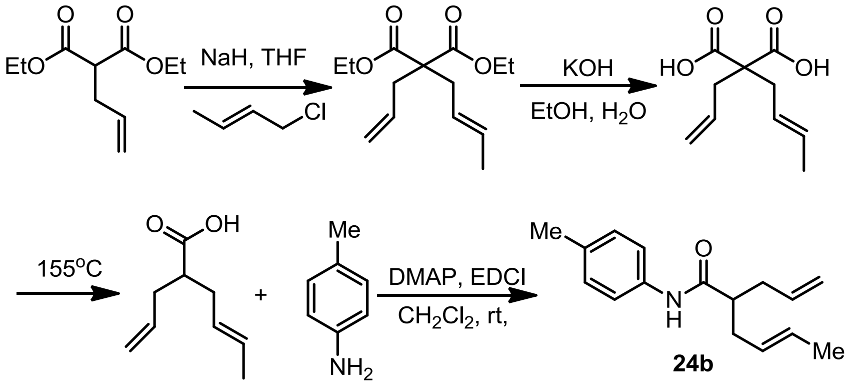

To a suspension of NaH (0.063 g, 60% in oil, 2.63 mmol) in THF (5 mL) was added dropwise Diethyl 2-allylmalonate (0.438 g, 2.19 mmol) dissolved in THF (5 mL) at 0 °C. The reaction mixture was warmed to room temperature and stirred for 1 h. (E)-1-chlorobut-2-ene (0.238 g, 2.63 mmol) was added, and the resulting solution was heated under reflux for 6 h. After the addition of H_2O (10 mL) and extraction with diethyl ether, the organic layer was washed with brine, dried with Na_2SO_4, and concentrated under reduced pressure. The crude product was purified by column chromatography to afford (E)-diethyl 2-allyl-2-(but-2-en-1-yl)malonate (0.363g) in 65% yield. (E)-diethyl 2-allyl-2-(but-2-en-1-yl)malonate (0.363 g, 1.43 mmol) dissolved in EtOH (2 mL) and H_2O (1 mL), KOH (0.4 g, 7.15 mmol) was added then heated under reflux for 4 h, the pH of the solvent was adjusted to 2 by HCl (3 M), extraction with Et_2O, the organic layer was washed with brine, dried over anhydrous Na_2SO_4, and concentrated under reduced pressure to give (E)-2-allyl-2-(but-2-en-1-yl)malonic acid. This crude product can be used without further purification. (E)-2-allyl-2-(but-2-en-1-yl)malonic acid was heated at 155 °C for 5 h to give (E)-2-allylhex-4-enoic acid.

(E)-2-Allyl-N-(p-tolyl)hex-4-enamide (**24b**): ^{1}H NMR (400 MHz, $CDCl_3$): δ ppm 7.37 (d, J = 8.0 Hz, 3H), 7.10 (d, J = 8.0 Hz, 2H), 5.86–5.77 (m, 1H), 5.57–5.49 (m, 1H), 5.48–5.38 (m, 1H), 5.12–5.02 (m, 2H), 2.48–2.20 (m, 8H), 1.63 (d, J = 5.6 Hz, 3H). ^{13}C NMR (100 MHz, $CDCl_3$): δ ppm 173.10, 135.85, 135.42, 133.97, 129.51, 128.08, 128.05, 120.35, 117.15, 48.52, 36.58, 35.55, 20.93, 18.04. FT–IR (film): ν (cm–1) 3295, 2918, 1657, 1602, 1537, 1515, 1407, 1310, 1250, 816. ESI-MS: $[M + Na]^+$ 266.0. HRMS (ESI) m/z calculated for $C_{16}H_{21}NONa$: $[M + Na]^+$ 266.1515; found 266.1516.

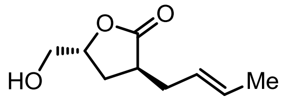

25b *anti*

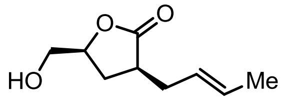

25b *syn*

^{1}H NMR (400 MHz, $CDCl_3$): δ ppm 5.59–5.52 (m, 1H), 5.42–5.35 (m, 1H), 4.58–4.55 (m, 0.49H), 4.52–4.49 (m, 0.50H), 3.92–3.84 (m, 1H), 3.66–3.60 (m, 1H), 2.81–2.71 (m, 1H), 2.57–2.44 (m, 1H), 2.33–2.18 (m, 3H), 2.09–2.02 (m, 0.55H), 1.88–1.79 (m, 0.5H), 1.68–1.63 (m, 2H);
^{13}C NMR (100 MHz, $CDCl_3$, *anti*): δ ppm 179.68, 128.72, 126.64, 78.88, 64.43, 39.72, 33.96, 28.64, 17.88; ^{13}C NMR (100 MHz, $CDCl_3$, *syn*): δ ppm 178.54, 128.38, 126.88, 79.08, 63.79, 40.73, 33.17, 29.00, 17.88;FT–IR (film): ν (cm^{-1}): 3442, 2922, 1764, 1554, 1266, 1180, 1061, 910, 738,

704. ESI-MS: $[M + Na]^+$ 193.1; HRMS (ESI) *m/z* calculated for $C_9H_{14}O_3Na$ $[M + Na]^+$ 193.0835, found 193.0836.

(*E*)-Ethyl 4-methyl-5-phenylpent-4-enoate (**24c**) was prepared according to the known procedure [11].

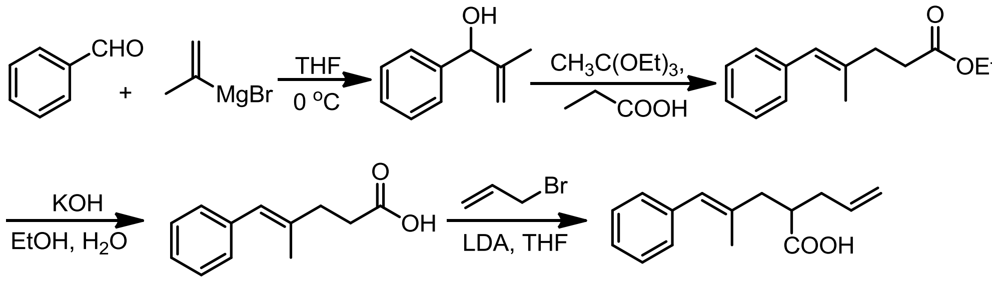

To a solution of benzaldehyde (1.06 g, 10 mmol) in THF (10 mL) was added 2-propenylmagnesium bromide (0.5 M in THF, 30 mL, 15 mmol) dropwise at 0 °C, the mixture was stirred for 24 h at 0 °C. Then the reaction was quenched with saturated NH_4Cl solution (20 mL), extracted with Et_2O (2 × 40 mL), washed with brine, dried over anhydrous Na_2SO_4, filtered and concentrated under reduced pressure. The residue was subjected to purification by silica gel chromatography to give 2-methyl-1-phenylprop-2-en-1-ol as a colorless oil (1.12g, 76%). ^1H NMR (400 MHz, $CDCl_3$): δ ppm 7.39–7.33 (m, 4H), 7.30–7.26 (m, 1H), 5.21 (s, 1H), 5.15–5.14 (d, *J* = 3.2Hz, 1H), 4.96 (s, 1H), 1.92–1.91 (m, 1H), 1.61 (s, 3H); ESI-MS: $[M - H]^-$ 147.1.

A solution of the 2-methyl-1-phenylprop-2-en-1-ol (760 mg, 5.14 mmol) and propanoic acid (0.17mL) in triethyl orthoacetate (8.6 ml) was heated to 145 °C under stirring for 24 h. The solution was cooled to room temperature, washed with 2 M aqueous HCl (35 mL), saturated aqueous $NaHCO_3$ solution (35 mL) and brine (35 mL), dried over anhydrous Na_2SO_4, filtered and concentrated under reduced pressure. The residue was subjected to purification by silica gel chromatography to give (*E*)-ethyl 4-methyl-5-phenylpent-4-enoate in 75% yield. ^1H NMR (400 MHz, $CDCl_3$): δ ppm 7.33–7.19 (m, 5H), 6.30 (s, 1H), 4.18–4.12 (q, *J* = 7.2Hz, 2H), 2.53–2.51 (m, 4H), 1.87–1.86 (d, *J* = 0.9 Hz, 3H), 1.28–1.25 (t, *J* = 7.2 Hz, 3H); ESI-MS: $[M + Na]^+$ 241.1. Acid after hydrolysis: ^1H NMR (400 MHz, $CDCl_3$): δ ppm 7.33–7.30 (m, 2H), 7.23–7.19 (m, 3H), 6.32 (s, 3H), 2.62–2.58 (m, 2H), 2.53–2.50 (m, 2H), 1.87 (s, 3H); ESI-MS: $[M + Na]^+$ 213.0.

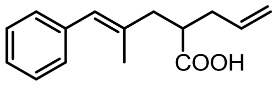

^1H NMR (400 MHz, $CDCl_3$): δ ppm 7.32–7.16 (m, 5H), 6.32 (s, 1H), 5.82–5.74 (m, 1H), 5.12–5.03 (m, 2H), 2.76–2.71 (m, 1H), 2.53–2.48 (m, 1H), 2.44–2.28 (m, 3H), 1.84 (s, 3H); ^{13}C NMR (100 MHz, $CDCl_3$): δ ppm 181.56, 138.08, 135.33, 134.97, 128.89, 128.08, 127.71, 126.23, 117.35, 43.92, 42.68, 35.76, 17.57; ESI-MS: $[M + Na]^+$ 253.1. HRMS (ESI) *m/z* calculated for $C_{15}H_{18}O_2Na$ $[M + Na]^+$ 253.1199, found 253.1201; FT–IR (film): ν (cm^{-1}): 3024, 2917, 1708, 1643, 1599, 1493, 1443, 1285, 1249, 1212, 1074, 993, 918, 743, 699, 513.

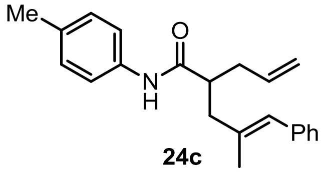

24c

^1H NMR (400 MHz, $CDCl_3$): δ ppm 7.36–7.34 (d, *J* = 8.4 Hz, 2H), 7.30–7.23 (m, 2H), 7.19–7.16 (m, 3H), 7.09–7.07 (d, *J* = 8.0 Hz, 2H), 6.37 (s, 1H), 5.88–5.81 (m, 1H), 5.16–5.06 (m, 2H), 2.62–2.47

(m, 3H), 2.42–2.32 (m, 2H), 2.29 (s, 3H), 1.88 (s, 3H); ^{13}C NMR (100 MHz, $CDCl_3$): δ ppm 172.83, 138.02, 135.91, 135.66, 135.17, 134.04, 129.47, 128.84, 128.10, 127.87, 126.23, 120.33, 117.38, 47.07, 43.30, 36.74, 20.86, 18.00; ESI-MS: $[M + Na]^+$ 342.2; HRMS (ESI) *m/z* calculated for $C_{22}H_{25}NONa$ $[M + Na]^+$ 342.1828, found 342.1843; FT–IR (film): ν (cm^{-1}):3246, 2918, 1652, 1600, 1533, 1310, 919, 813, 740, 698.

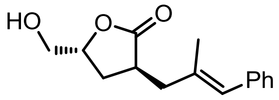

25c *anti*

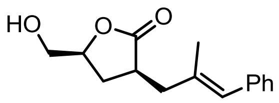

25c *syn*

^1H NMR (400 MHz, $CDCl_3$): δ ppm 7.34–7.31 (t, *J* = 7.6 Hz, 2H), 7.24–7.19 (m, 3H), 6.33 (s, 1H), 4.64–4.62 (m, 0.60H), 4.55–4.53 (m, 0.40H), 3.95–3.88 (m, 1H), 3.68–3.62 (m, 1H), 3.03–2.75 (m, 2H), 2.37–2.23 (m, 2.41H), 2.15–2.07 (m, 0.59H), 1.87 (s, 3H); ^{13}C NMR (100 MHz, $CDCl_3$, *anti*): δ ppm 179.68, 137.73, 135.14, 128.86, 127.81, 126.44, 78.71, 64.56, 42.16, 38.40, 29.04; ^{13}C NMR (100 MHz, $CDCl_3$, *syn*): δ ppm 178.64, 137.78, 135.21, 128.17, 127.50, 126.40, 78.98, 63.72, 41.50, 39.48, 29.49; $[M + Na]^+$ 269.1; HRMS (ESI) *m/z* calculated for $C_{15}H_{18}O_3Na$ $[M + Na]^+$ 269.1154, found 269.1157; FT–IR (film): ν (cm^{-1}): 3427, 3022, 2926, 2855, 1767, 1599, 1491, 1445, 1360, 1280, 1183, 1071, 1029, 966, 922, 805, 747, 700, 641, 618, 519.

(*E*)-2-Allyl-5-phenyl-*N*-(*p*-tolyl)pent-4-enamide (**24d**) was synthesized according to a procedure similar to the preparation of (*E*)-2-allyl-*N*-(*p*-tolyl)hex-4-enamide (**24b**) with 60% yield.

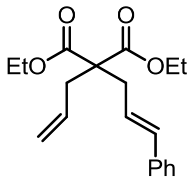

^1H NMR (400 MHz, $CDCl_3$): δ ppm 7.33–7.26 (m, 4H), 7.23–7.19 (m, 1H), 6.46–6.42 (d, *J* = 15.2 Hz, 1H), 6.08–6.01 (m, 1H), 5.74–5.67 (m, 1H), 5.16–5.12 (m, 2H), 4.22–4.17 (q, *J* = 7.2 Hz, 4H), 2.80–2.78 (dd, *J* = 1.2, 7.2 Hz, 2H), 2.70–2.68 (d, *J* = 7.2 Hz, 2H), 1.26–1.23 (t, *J* = 7.2 Hz, 6H).

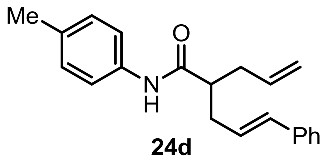

24d

^1H NMR (400 MHz, $CDCl_3$): δ ppm 7.36–7.34 (d, *J* = 8.4 Hz, 2H), 7.32–7.24 (m, 4H), 7.21–7.17 (m, 1H), 7.09–7.07 (d, *J* = 8.4 Hz, 2H), 6.48–6.44 (d, *J* = 16 Hz, 1H), 6.23–6.15 (m, 1H), 5.86–5.79 (m, 1H), 5.15–5.05 (m, 1H), 2.66–2.59 (m, 1H), 2.52–2.31 (m, 4H), 2.29 (s, 3H); ^{13}C NMR (100 MHz, $CDCl_3$): δ ppm 172.78, 137.32, 135.54, 135.18, 134.05, 132.55, 129.46, 128.56, 127.28, 127.17, 126.18, 120.41, 117.40, 48.38, 36.69, 35.82, 20.87; FT–IR (film): ν (cm^{-1}): 3284, 2922, 1653, 1601, 1514, 1310, 966, 912, 815, 740; ESI-MS: $[M + Na]^+$ 328.2; HRMS (ESI) *m/z* calculated for $C_{21}H_{23}NONa$: $[M + Na]^+$ 328.1672, found 328.1683.

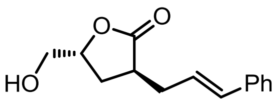

25d *anti*

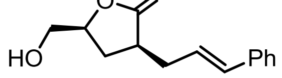

25d *syn*

^1H NMR (400 MHz, $CDCl_3$): δ ppm 7.35–7.27 (m, 4H), 7.23–7.19 (m, 1H), 6.49–6.45 (d, *J* = 15.6 Hz, 1H), 6.17–6.10 (m, 1H), 4.58–4.53 (m, 0.67H), 4.52–4.49 (m, 0.34H), 3.89–3.82 (m, 1H), 3.63–3.58 (m, 1H), 2.97–2.90 (m, 1H), 2.84–2.65 (m, 2H), 2.47–2.39 (m, 1H), 2.35–2.26 (m, 1H), 2.12–2.03 (m, 1H), 2.12–2.03 (m, 0.68H), 1.93–1.84 (m, .035 H); ^{13}C NMR (100 MHz, $CDCl_3$, *anti*): δ ppm 179.57, 136.97, 133.15, 128.63, 127.53, 126.23, 125.74, 79.00 , 64.37, 39.70, 34.41, 28.82; ^{13}C NMR (100 MHz, $CDCl_3$, *syn*): δ ppm 178.52, 137.05, 132.88, 128.63, 127.48, 126.20, 126.02, 79.26, 63.62, 40.74, 33.63, 29.05; FT–IR (film):

ν (cm^{-1}): 3431, 2927, 1766, 1449, 1363, 1179, 1071, 967, 747, 695; ESI-MS: $[M + Na]^+$ 255.1; HRMS (ESI) *m*/*z* calculated for $C_{14}H_{16}O_3Na$ 255.0992, found 255.0991.

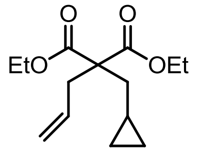

2-(Cyclopropylmethyl)-*N*-(*p*-tolyl)pent-4-enamide (**24e**) was synthesized according to a procedure similar to the preparation of (*E*)-2-allyl-*N*-(*p*-tolyl) hex-4-enamide (**24b**) with 70% yield.

^{1}H NMR (400 MHz, $CDCl_3$): δ ppm 5.69–5.58 (m, 1H), 5.10–5.04 (m, 2H), 4.21–4.08 (m, 4H), 2.75–2.73 (d, *J* = 7.2 Hz, 2H), 1.81–1.79 (d, *J* = 7.2 Hz, 2H), 1.24–1.20 (t, *J* = 7.2 Hz, 6H), 0.64–0.57 (m, 1H), 0.42–0.38 (m, 2H), 0.04–0.01 (m, 2H); ^{13}C NMR (100 MHz, $CDCl_3$): δ ppm 171.35, 132.76, 118.77, 61.04, 57.87, 37.07, 36.90, 14.06, 5.90, 4.22; FT–IR (film): ν(cm^{-1}): 3080, 2982, 1733, 1445, 1367, 1222, 1153, 1019, 920,859. ESI-MS: $[M + Na]^+$ 277.2; HRMS (ESI) *m*/*z* calculated for $C_{14}H_{22}O_4$ Na: $[M + Na]^+$ 277.1410, found 277.1417.

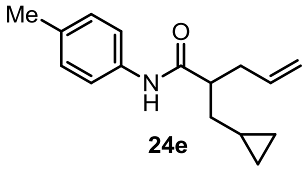

24e

^{1}H NMR (400 MHz, $CDCl_3$): δ ppm 7.42–7.40 (d, *J* = 8.4 Hz, 2H), 7.11–7.09 (d, *J* = 8.4 Hz, 2H), 5.83–5.75 (m, 1H), 5.11–5.01 (m, 2H), 2.49–2.44 (m, 1H), 2.40–2.35 (m, 1H), 2.30 (s, 3H), 2.28–2.24 (m, 1H), 1.72–1.65 (m, 1H), 1.41–1.34 (m, 1H), 0.74–0.70 (m, 1H), 0.45–0.41 (m, 2H), 0.13–0.02 (m, 2H); ^{13}C NMR (100 MHz, $CDCl_3$): δ ppm 173.61, 135.84, 135.43, 133.84, 129.43, 120.20, 116.91, 49.02, 37.61, 37.00, 20.85, 9.23, 4.79, 4.36; FT–IR (film): ν(cm^{-1}): 3292, 3077, 2921, 1655, 1608, 1514, 1310, 1255, 1017, 816; ESI-MS: $[M + Na]^+$ 266.1, $[2M + Na]^+$ 509.3; HRMS (ESI) *m*/*z* calculated for $C_{16}H_{21}NONa$ $[M + Na]^+$ 266.1515, found 266.1521.

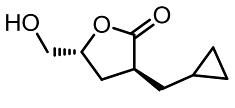

25e *anti*

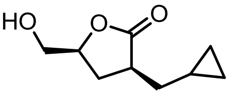

25e *syn*

^{1}H NMR (400 MHz, $CDCl_3$): δ ppm 4.64–4.59 (m, 0.65H), 4.53–4.48 (m, 0.33H), 3.93–3.85 (m, 1H), 3.67–3.64 (m, 1H), 2.87–2.72 (m, 1H), 2.55 (br, 0.63H), 2.45–2.33 (m, 1.31H), 2.18–2.10 (m, 0.68H), 1.97–1.88 (m, 0.34H), 1.74–1.63 (m, 1H), 1.50–1.42 (m, 1H), 0.79–0.70 (m, 1H), 0.53–0.43 (m, 2H), 0.16–0.05 (m, 2H); ^{13}C NMR (100 MHz, $CDCl_3$, *anti*): δ ppm 180.16, 79.02, 64.37, 40.17, 35.95, 29.42, 8.76, 4.74, 4.27; ^{13}C NMR (100 MHz, $CDCl_3$, *syn*): δ ppm 179.12, 79.22, 63.65, 41.31, 35.06, 29.65, 8.93, 4.77, 4.17. FT–IR (film): ν (cm^{-1}): 3443, 3001, 2927, 1770, 1357, 1188, 1055, 1020, 950, 619; EI-MS: m/z (%) 31.1 (18), 41.1 (47), 55.1 (90), 67.1 (70), 73.1 (51), 95.1 (55), 116.1 (100), 129.1 (20), 139.1 (20), 170.1 (2); HRMS (ESI) *m*/*z* calculated for $C_9H_{14}O_3$ (M^+) 170.0943, found: 170.0945.

2-Allyl-4-methyl-*N*-(*p*-tolyl)pent-4-enamide (**24f**) was synthesized according to a procedure similar to the preparation of (*E*)-2-allyl-*N*-(*p*-tolyl)hex-4-enamide (**24b**), with 65% yield.

^{1}H NMR (400 MHz, $CDCl_3$): δ ppm 5.74–5.64 (m, 1H), 5.11–5.07 (m, 2H), 4.87–4.86 (m, 1H), 4.76–4.76 (m, 1H), 4.21–4.14 (m, 4H), 2.70–2.66 (m, 4H), 1.67 (s, 3H), 1.27–1.23 (t, *J* = 6.8 Hz, 6H); ESI-MS: $[M + Na]^+$ 277.1.

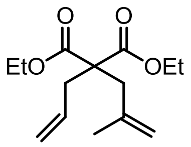

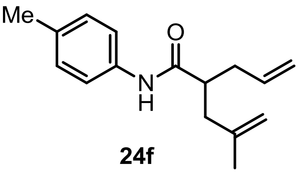

24f

^{1}H NMR (400 MHz, $CDCl_3$): δ ppm 7.37–7.35 (d, *J* = 8.0 Hz, 2H), 7.10–7.09 (d, *J* = 6.8 Hz, 2H), 5.82–5.79 (m, 1H), 5.13–5.04 (m, 2H), 4.82–4.78 (d, *J* = 13.2Hz, 2H), 2.45–2.43 (m, 3H), 2.30 (s, 3H), 2.25–2.23 (m, 2H), 1.75 (s, 3H); ^{13}C NMR (100 MHz, $CDCl_3$): δ ppm 173.00, 143.10, 135.65, 135.28, 134.01, 129.43, 120.26, 117.27, 112.72, 46.50, 40.31, 36.81, 22.58, 20.84; FT–IR (film): ν (cm^{-1}): 3293, 3079, 2919, 1655, 1608, 1514, 1249, 919, 818,513; ESI-MS: $[M + Na]^+$ 266.2; $[M + H]^+$ 244.2; HRMS (ESI) *m*/*z* calculated for $C_{16}H_{21}NONa$ $[M + Na]^+$ 266. 1515, found 266.1515.

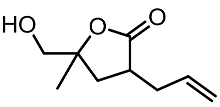

25f

^{1}H NMR (400 MHz, $CDCl_3$): δ ppm 5.74–5.65 (m, 1H), 5.08–5.01(m, 2H), 3.66–3.63 (d, J = 12.4 Hz, 0.6H), 3.62–3.59 (d, J = 12.4 Hz, 0.40H), 3.47–3.44 (d, J = 12.0 Hz, 0.40H), 3.41–3.38 (d, J = 12.4 Hz, 0.60H), 2.99–2.95 (m, 0.40H), 2.85–2.78 (m, 0.62H), 2.59–2.48 (m, 1.61H), 2.43–2.37 (m, 0.43 H), 2.25–2.18 (m, 1.32H), 2.09–2.03 (m, 0.61H), 1.97–1.92 (m, 0.63H), 1.68–1.62 (m, 0.40H), 1.30 (s, 1.19H), 1.29 (s, 1.83H); ^{13}C NMR (100 MHz, $CDCl_3$): δ ppm 179.23, 134.58, 117.64, 84.70, 68.86, 40.99, 36.35, 34.26, 23.97; ^{13}C NMR (100 MHz, $CDCl_3$): δ ppm 178.14, 134.66, 117.53, 84.61, 67.75, 39.91, 35.47, 34.58, 22.58; ESI-MS: $[M + Na]^+$ 328.2; HRMS (ESI) *m*/*z* calculated for $C_{21}H_{23}NONa$: $[M + Na]^+$ 328.1672, found 328.1683. FT–IR (film): ν (cm^{-1}): 3424, 2926, 1766, 1642, 1441, 1359, 1260, 1226, 1184, 1101, 1054, 1022, 923, 801,659.

(*S*)-(1-(4-Methoxyphenyl)pyrrolidin-2-yl)methanol (**3a**) was prepared according to a known procedure, with 66% yield [12].

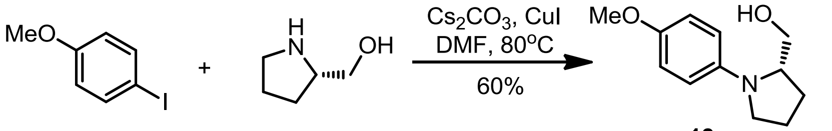

13a

Oven-dried Cs_2CO_3 (0.330 g, 1.0 mmol) and CuI (19mg, 0.1mmol) were added to the solution of 1-iodo-4-methoxybenzene (0.117 g, 0.5 mmol) and (S)-pyrrolidin-2- ylmethanol (0.152, 1.5 mmol) in DMF (3.2 mL) in a N_2 atmosphere. The mixture stirred at 80 °C for 8 h, then cooled to room temperature, the solid was filtered, the filtrate was concentrated under reduced pressure, and flash chromatography of the residue over silica gel by using EtOAc–hexane mixtures (4:1) gave **13a** in 60% yield.^{1}H NMR (400 MHz, $CDCl_3$): δ ppm 6.85 (d, *J* = 9.2 Hz, 2H), 6.67 (d, *J* = 9.2 Hz, 2H), 3.76 (s, 4H), 3.60–3.67 (m, 2H), 3.48–3.51 (m, 1H), 3.08–3.12 (m, 2H), 1.95–2.08 (m, 5H); ^{13}C NMR(100 MHz, CDCl3): δ ppm 151.36, 143.11, 115.10, 113.38, 63.91, 60.94, 56.05, 50.33, 28.93, 24.02. ESI-MS: $[M + H]^+$ 208.1. HRMS (ESI): *m*/*z* calculated for $C_{12}H_{18}NO_2$: $[M + H]^+$ 208.1332; found: 208.1334.

1-(4-methoxyphenyl)piperidin-3-ol (**13b**) was prepared in a similar manner with physical and spectra data in agreement with those reported [10].

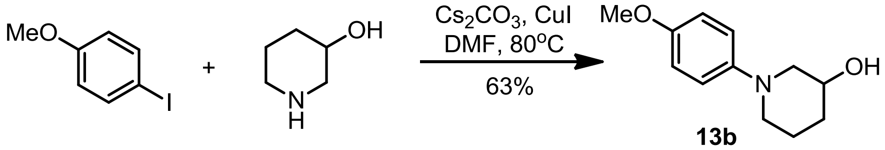

13b

^{1}H NMR (400 MHz，CDCl$_3$): δ ppm 6.92 (d, *J* = 9.2 Hz, 2H), 6.83 (d, *J* = 9.2 Hz, 2H), 3.94–3.95 (m, 1H), 3.77 (s, 3H), 3.13–3.16 (m, 1H), 2.94–3.02 (m, 3H), 1.92–1.95 (m, 1H), 1.61–1.78 (m, 4H); ^{13}C NMR (100 MHz, CDCl$_3$): δ ppm 154.28, 146.46, 119.68, 114.51, 66.56, 59.13, 55.67, 52.02, 31.88, 22.23. ESI-MS: $[M + H]^+$ 208.1. HRMS (ESI): *m*/*z* calculated for $C_{12}H_{18}NO_2$: $[M + H]^+$ 208.1332; found: 208.1335. FT–IR (film): ν (cm^{-1}) 3383, 2937, 2832, 1511, 1464, 1245, 1182, 1066, 1037, 824.

III. X-ray data for the revised compound **13**

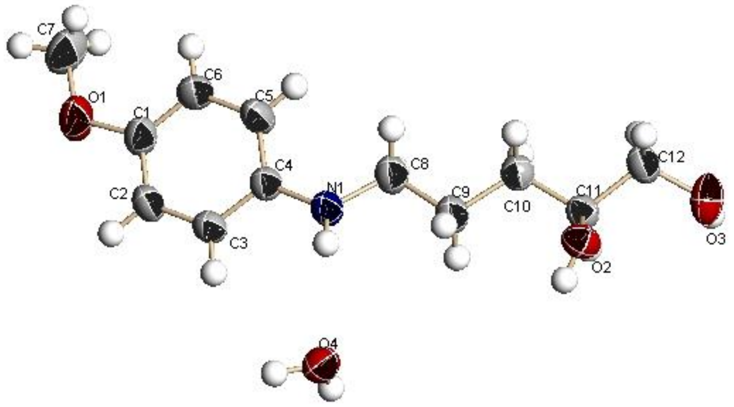

Bond precision: C-C = 0.0032 A Wavelength=0.71073

Cell:	a=14.488(2)	b=6.9113(9)	c=26.720(4)
	alpha=90	beta=100.845(2)	gamma=90

Temperature: 296 K

	Calculated	Reported
Volume	2627.7(6)	2627.7(6)
Space group	C 2/c	C2/c
Hall group	-C 2yc	
Moiety formula	2(C12 H19 N O3), H2 O	
Sum formula	C24 H40 N2 O7	C12 H20 N O3.50
Mr	468.58	234.29
Dx,g cm-3	1.184	1.184
Z	4	8
Mu (mm-1)	0.086	0.086
F000	1016.0	1016.0
F000'	1016.52	
h,k,lmax	17,8,32	17,8,32
Nref	2461	2453
Tmin,Tmax	0.978,0.985	0.660,0.746
Tmin'	0.970	

Correction method= MULTI-SCAN

Data completeness= 0.997 Theta(max)= 25.510

R(reflections)= 0.0558(1929) wR2(reflections)= 0.1684(2453)

S = 1.035 Npar= 157

IV.. X-ray data for compound **22a**

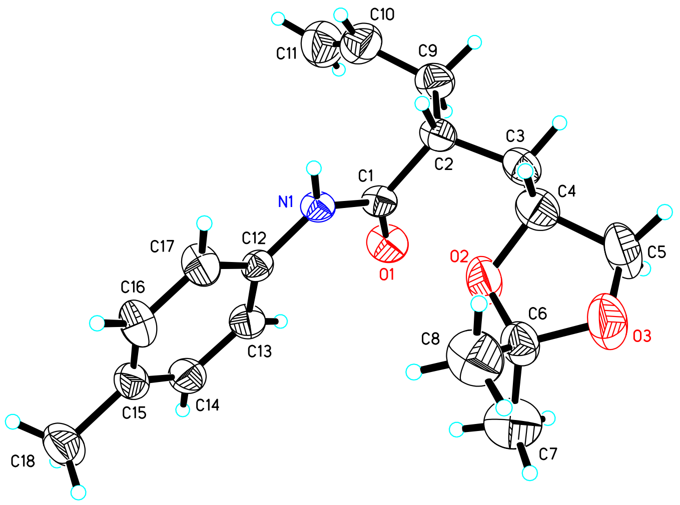

Bond precision:	C-C = 0.0051 A		Wavelength=0.71073
Cell:	a=10.109(4)	b=20.538(5)	c=9.718(2)
	alpha=90	beta=118.555(4)	gamma=90

Temperature: 296 K

	Calculated	Reported
Volume	1772.2(9)	1772.2(9)
Space group	P 21/c	P2(1)/c
Hall group	-P 2ybc	
Moiety formula	C18 H25 N O3	
Sum formula	C18 H25 N O3	C18 H25 N O3
Mr	303.39	303.39
Dx,g cm-3	1.137	1.137
Z	4	4
Mu (mm-1)	0.077	0.077
F000	656.0	656.0
F000'	656.30	
h,k,lmax	12,24,11	12,24,11
Nref	3211	3200
Tmin,Tmax	0.977,0.985	0.977,0.985
Tmin'	0.977	

Correction method= MULTI-SCAN

Data completeness= 0.997 Theta(max)= 25.250

R(reflections)= 0.0685(2505) wR2(reflections)= 0.2441(3200)

S = 0.976 Npar= 202

V. ^{1}H NMR and ^{13}C NMR spectra

(*S*)-(1-(4-methoxyphenyl)pyrrolidin-2-yl)methanol (**13a**)

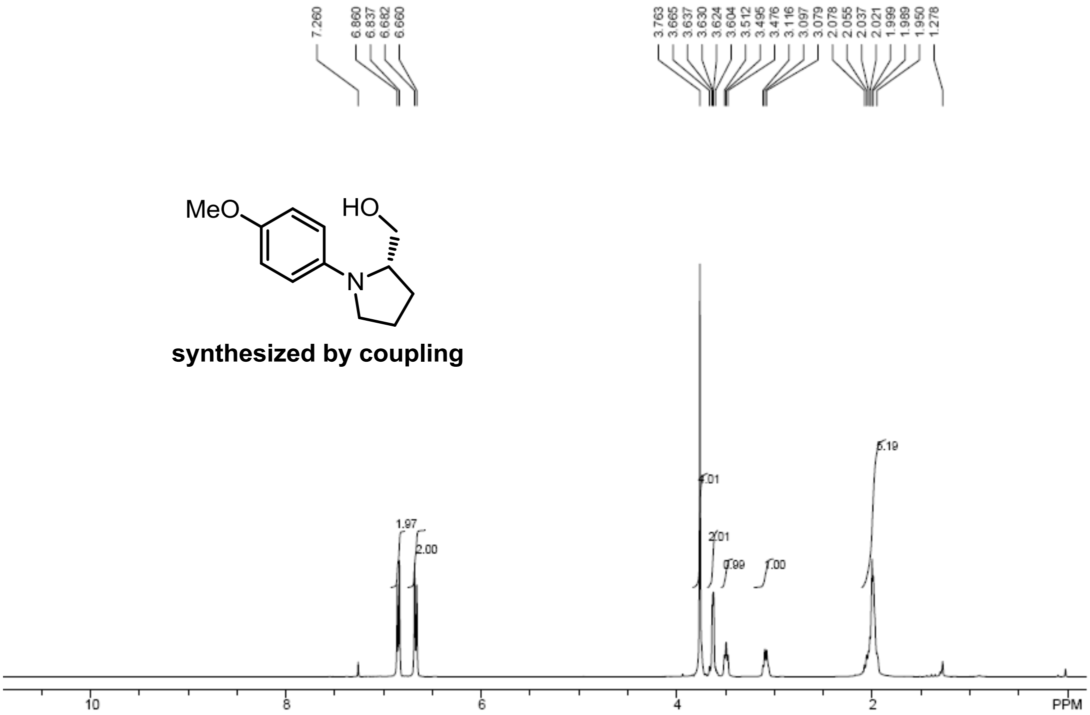

Pyrrolidine reported by Tellitu [13]

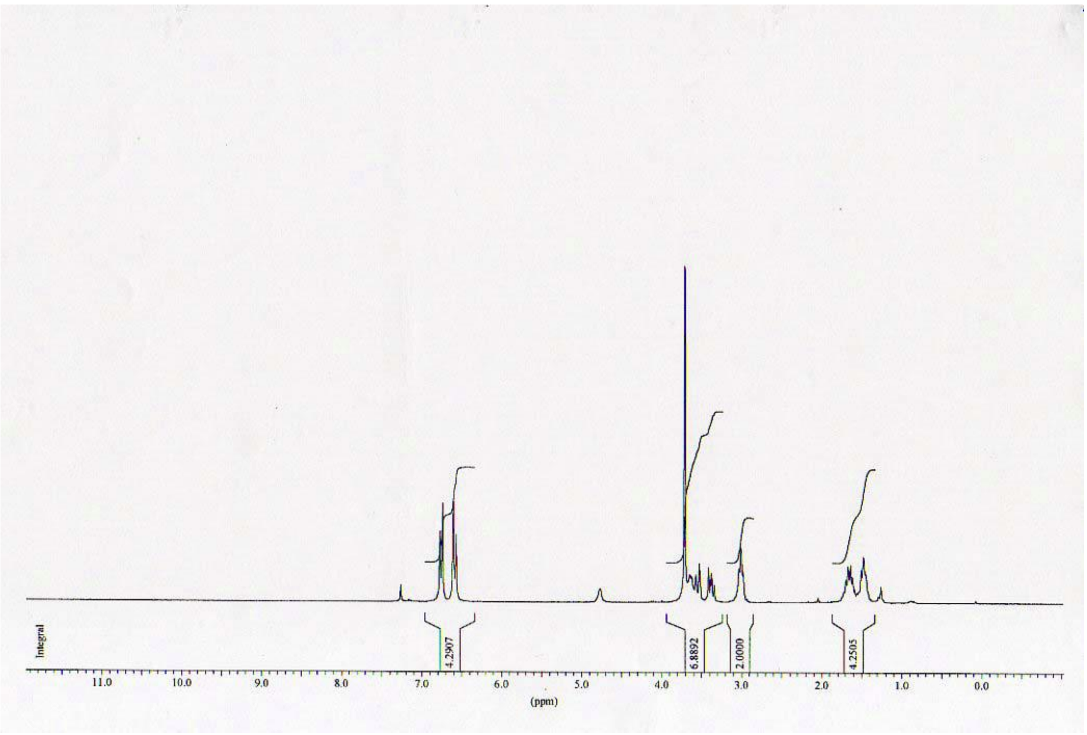

1-(4-Methoxyphenyl)piperidin-3-ol (**13b**)

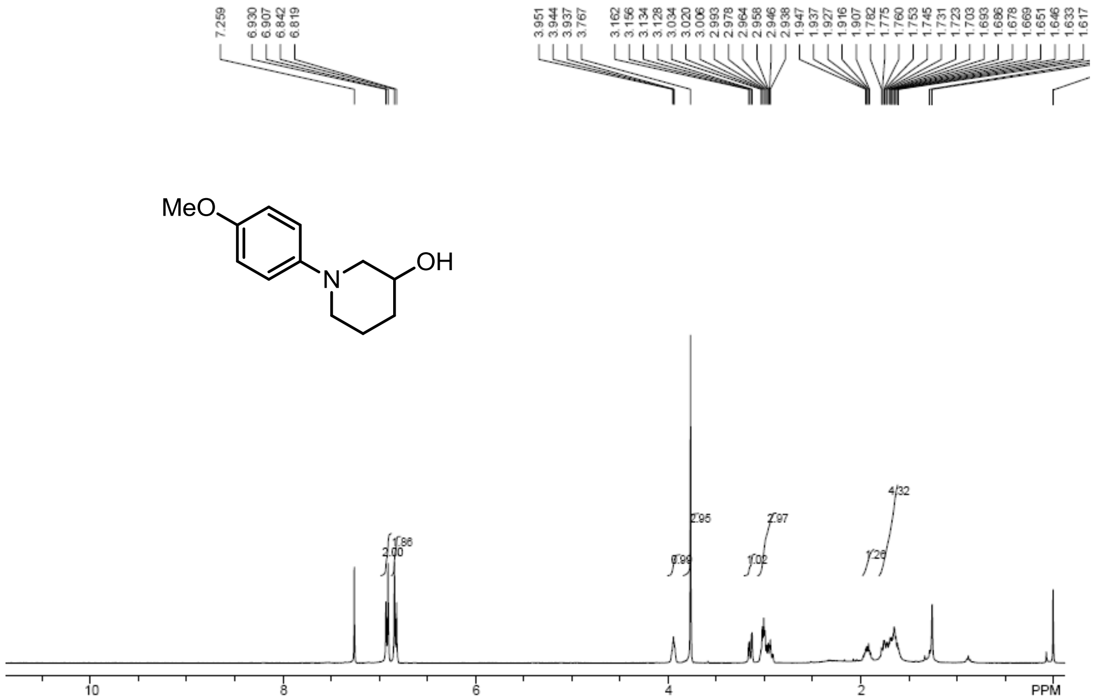

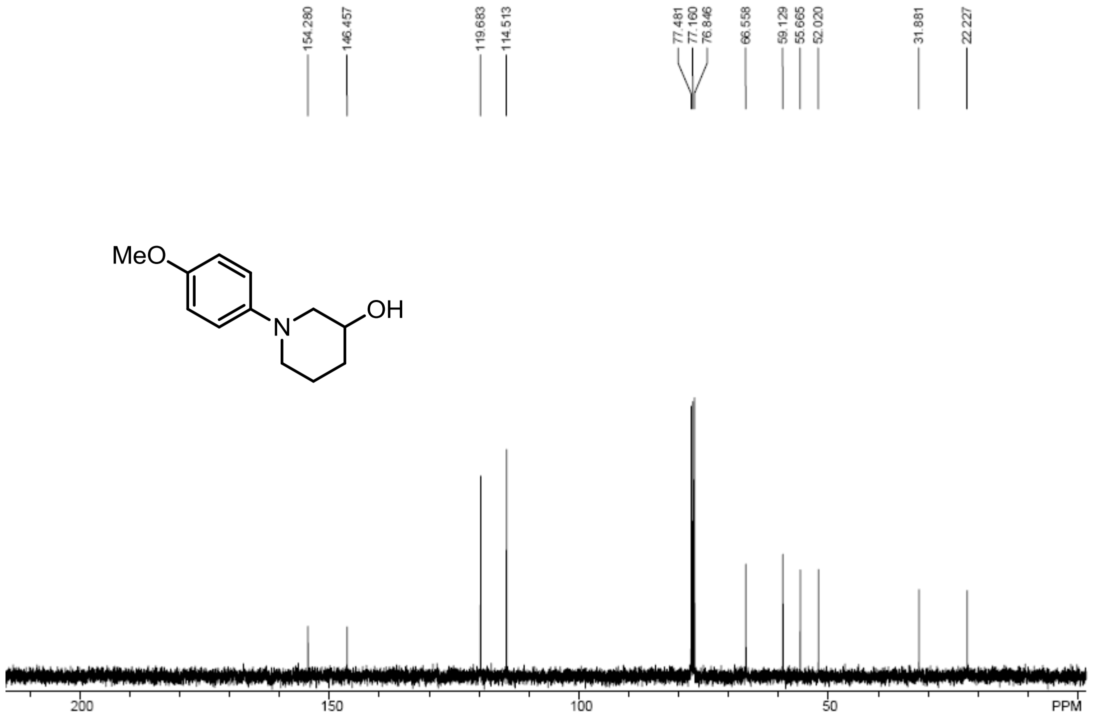

N- (4-Methoxyphenyl)pent-4-enamide (**11**)

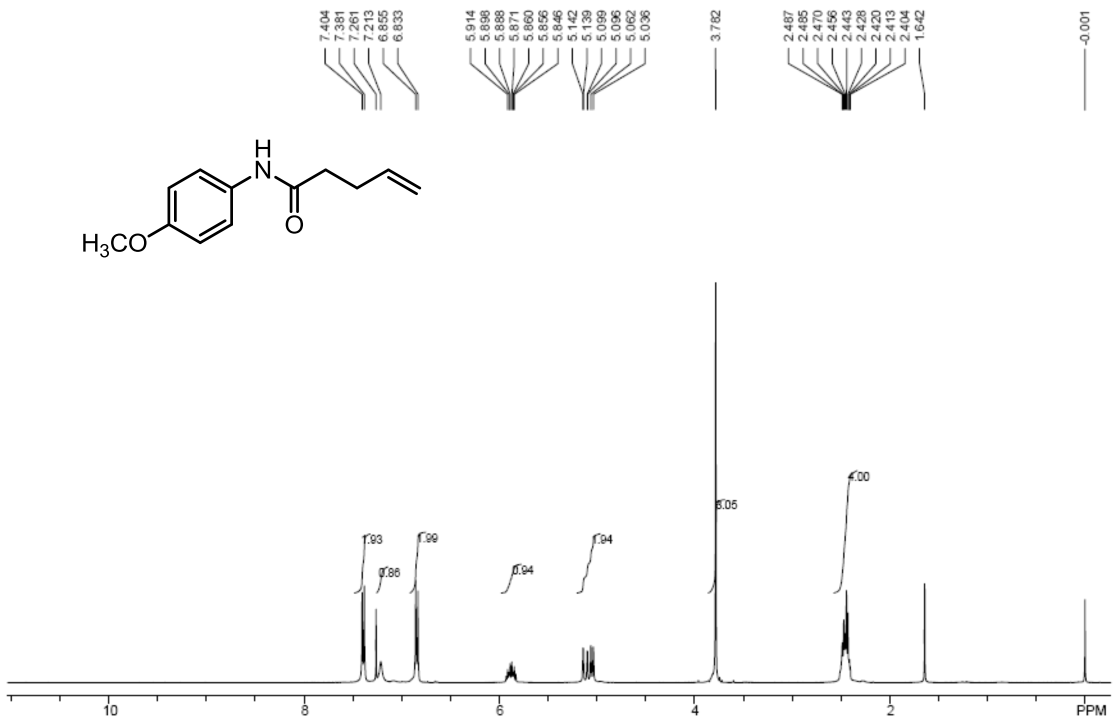

N-(*p*-Tolyl)pent-4-enamide (**18a**)

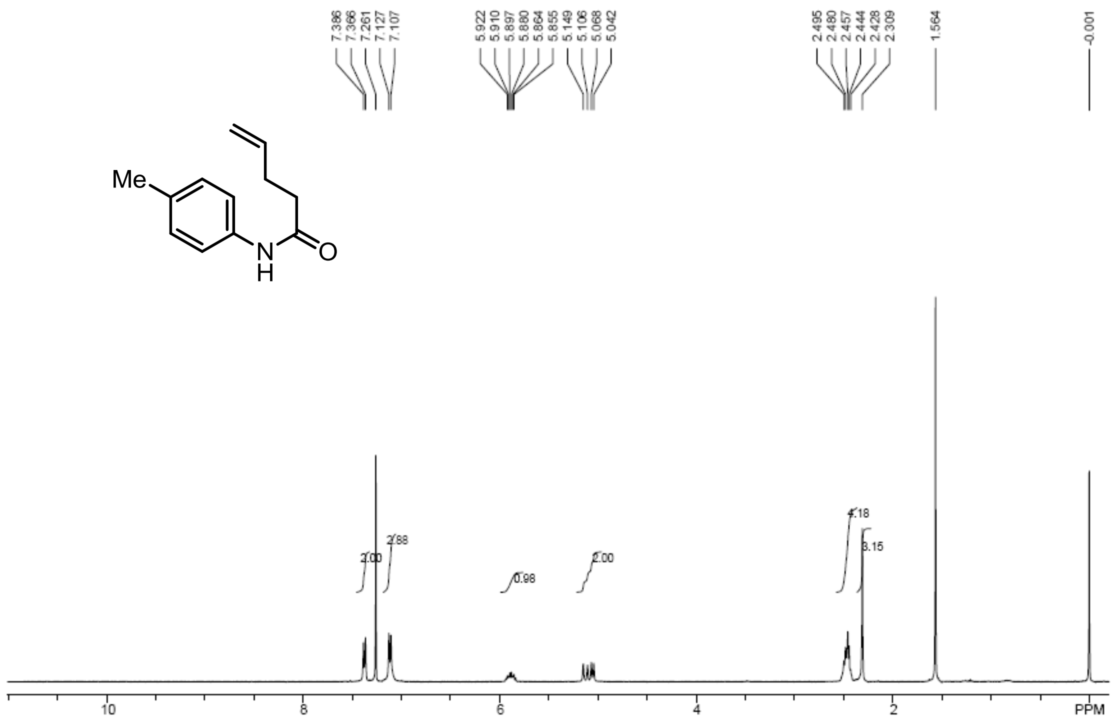

N-Phenylpent-4-enamide (**18b**)

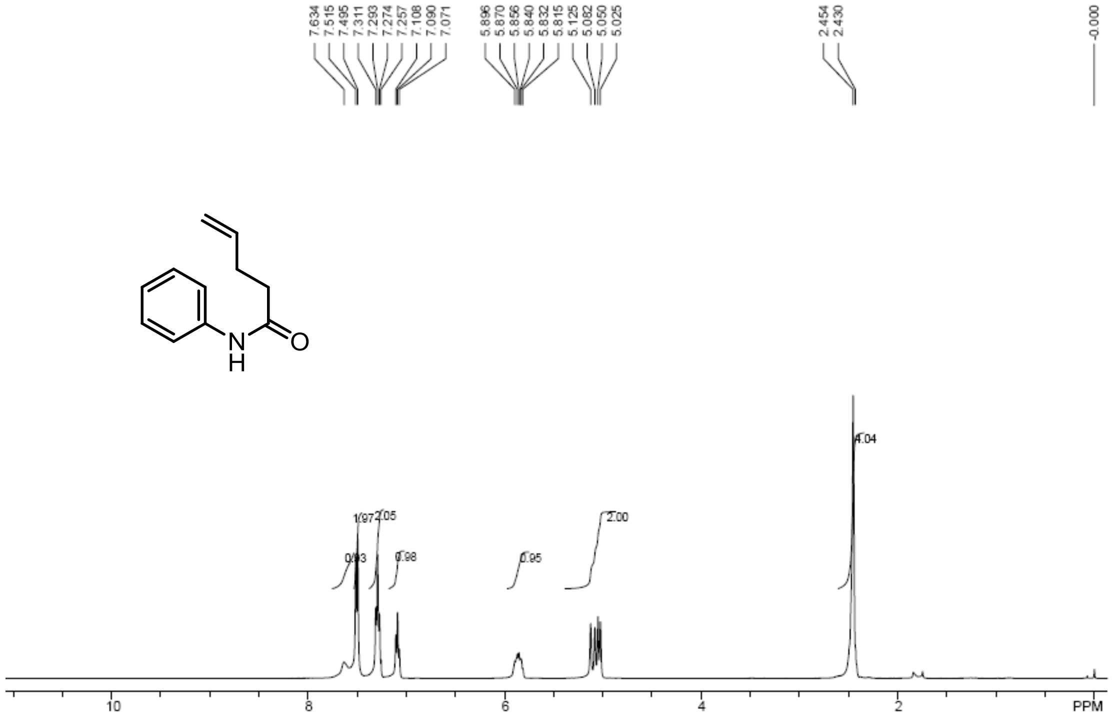

N-(4-Chlorophenyl)pent-4-enamide (**18c**)

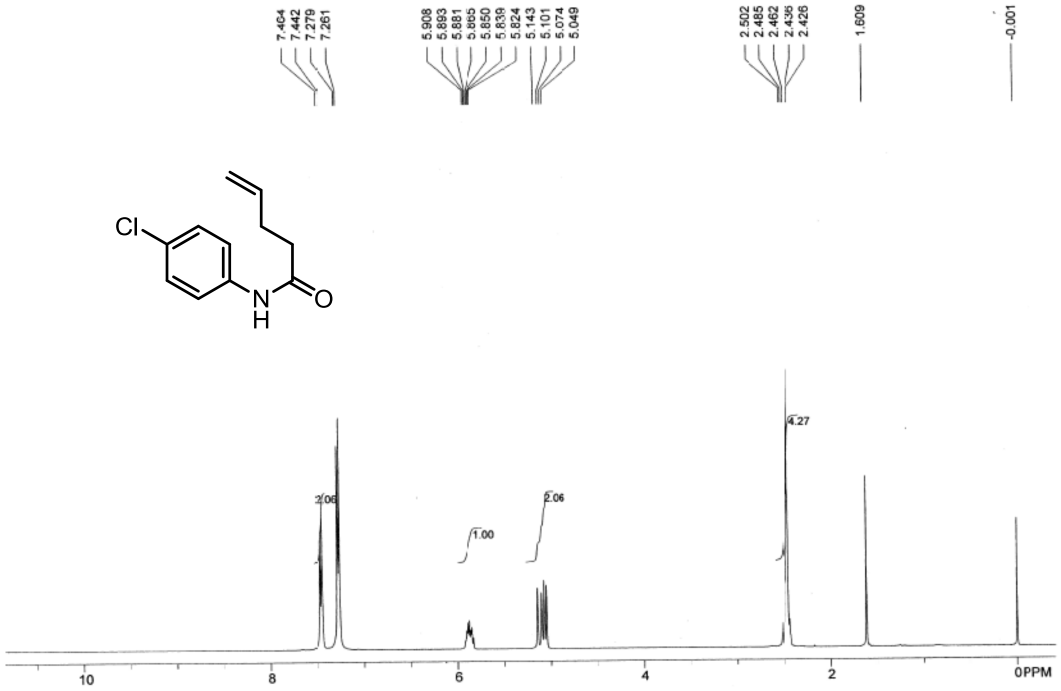

4-Methyl-*N*-(*p*-tolyl)pent-4-enamide (**18d**)

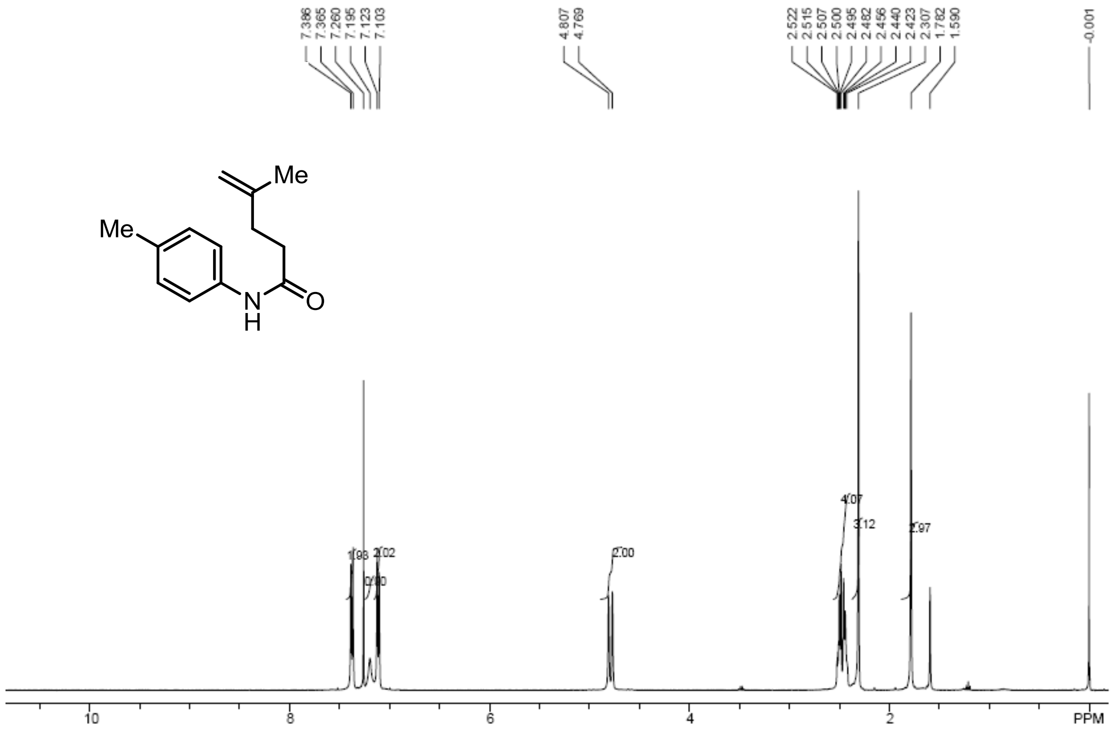

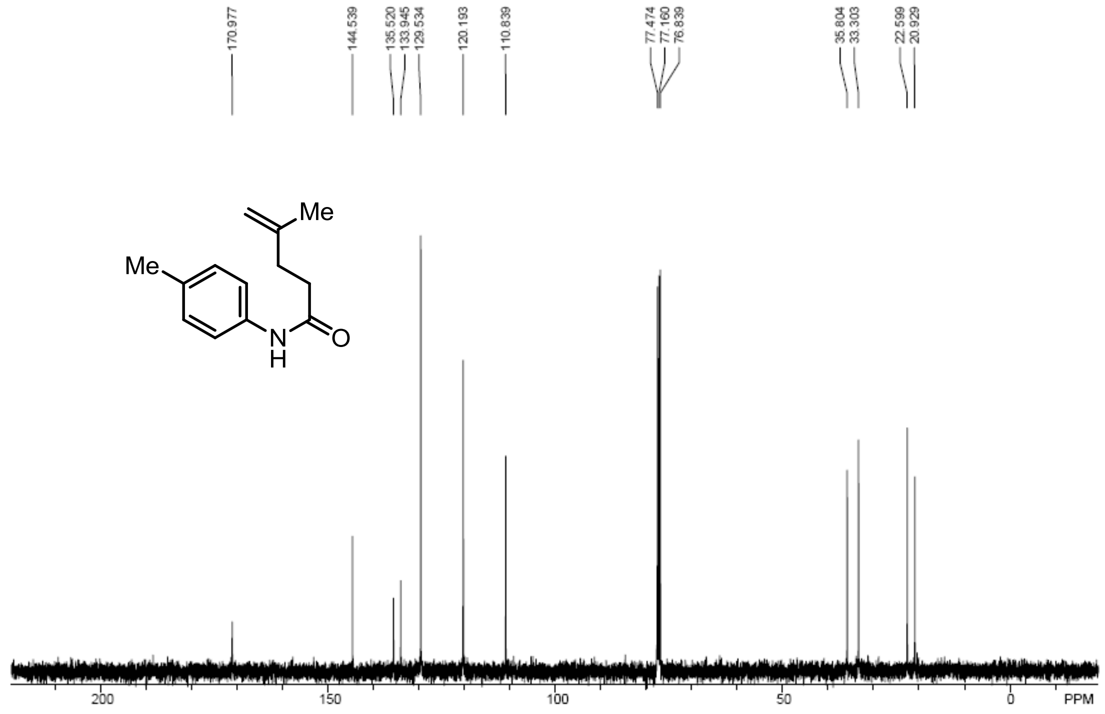

2-Methyl-*N*-(*p*-tolyl)pent-4-enamide (**18e**)

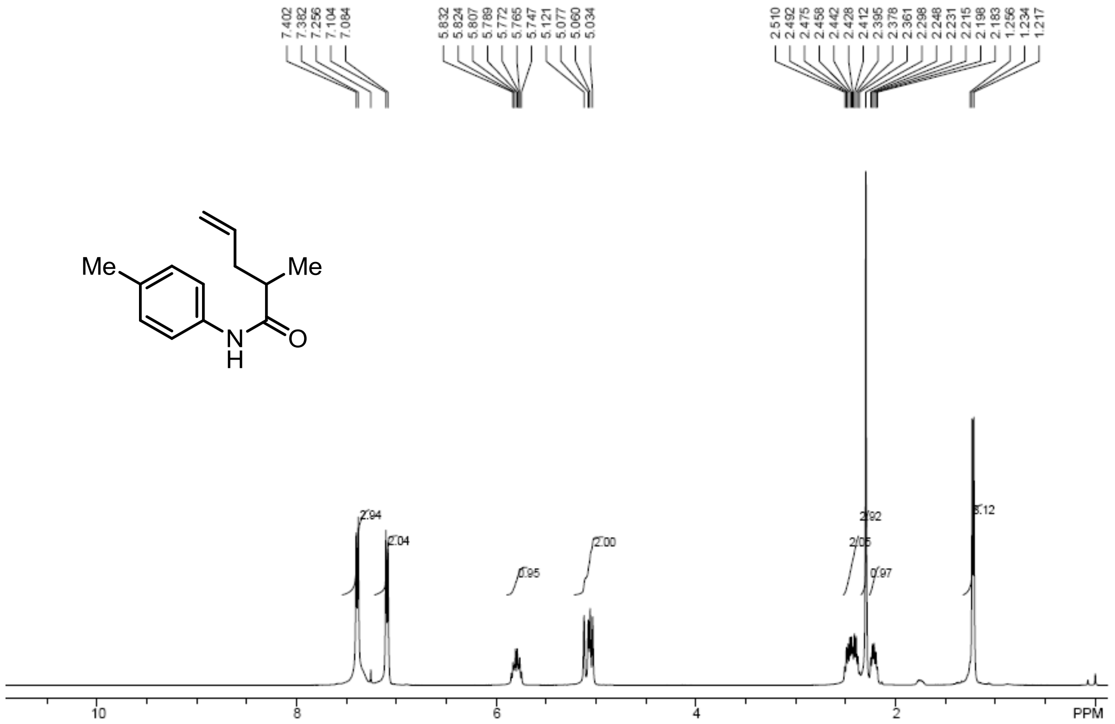

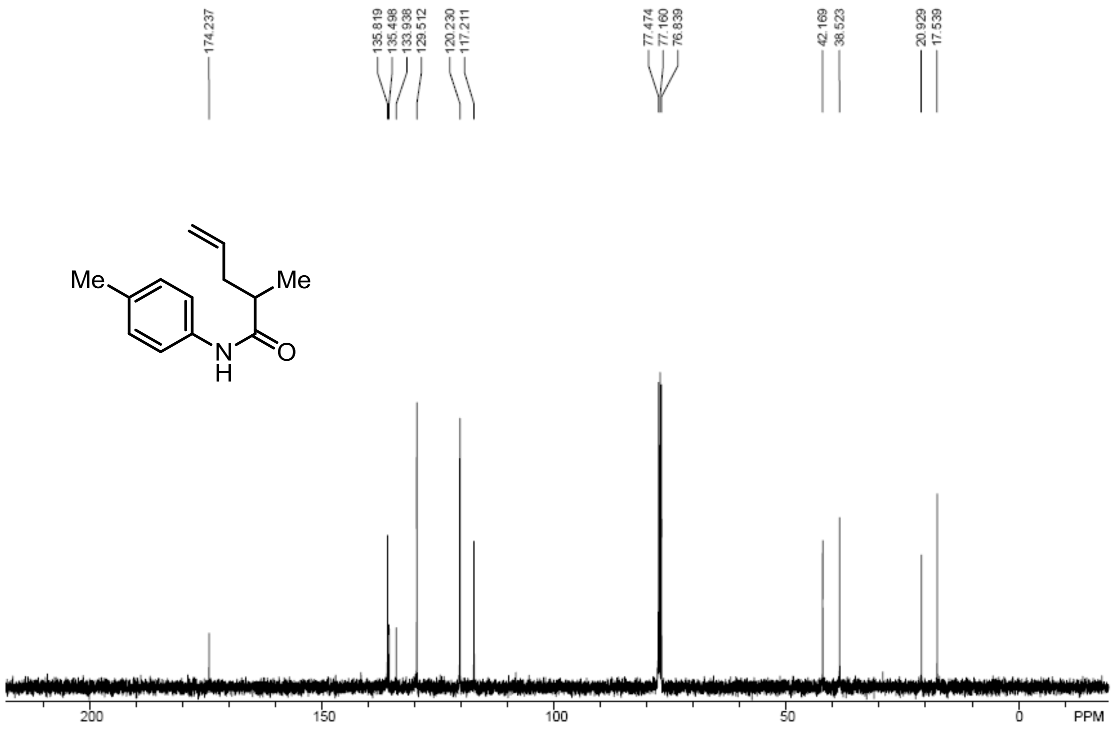

5-((4-Methoxyphenyl)amino)pentane-1,2-diol (**revised 13**)

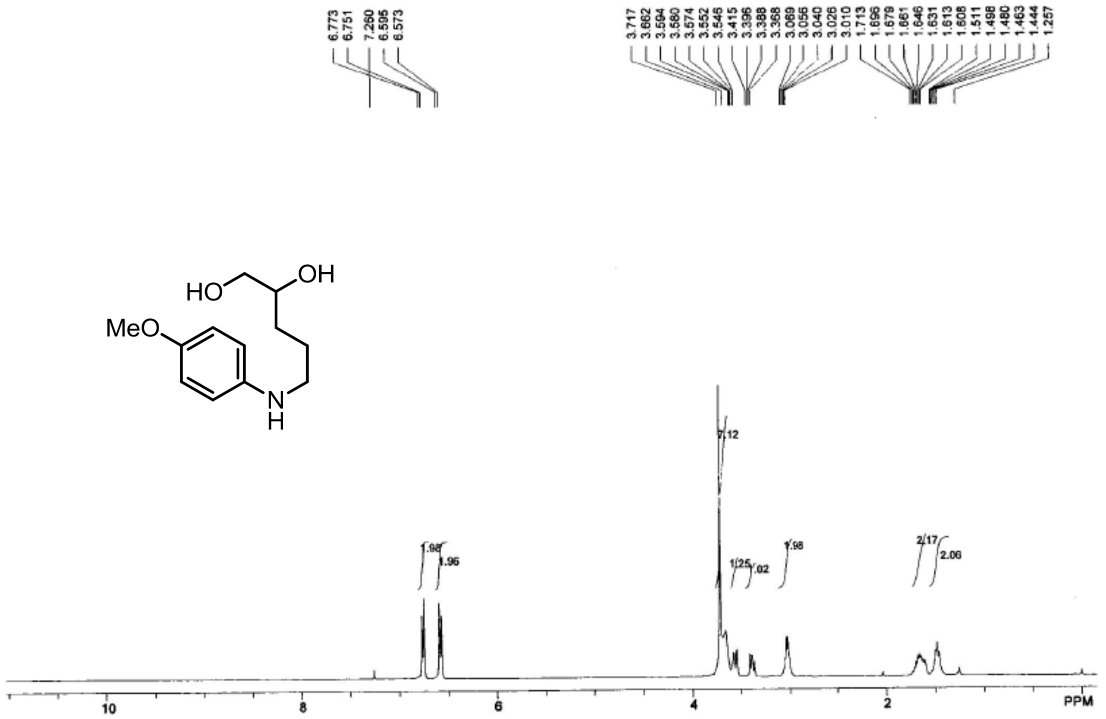

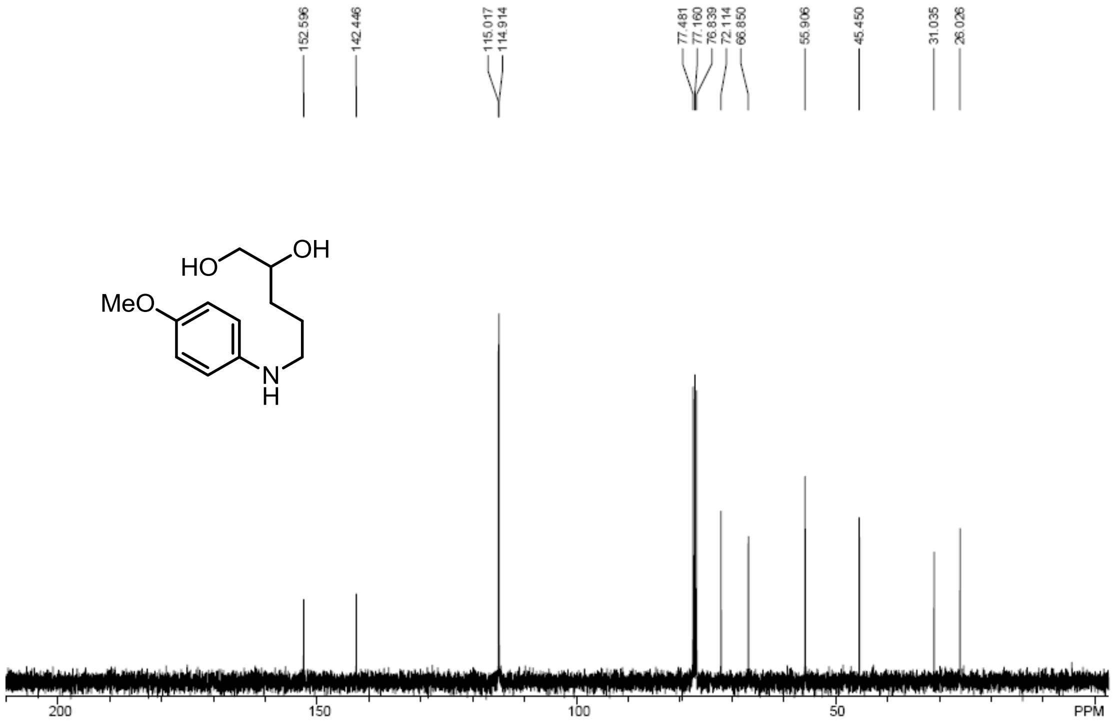

4,5-Dihydroxy-*N*-(4-methoxyphenyl)pentanamide (**revised 12**)

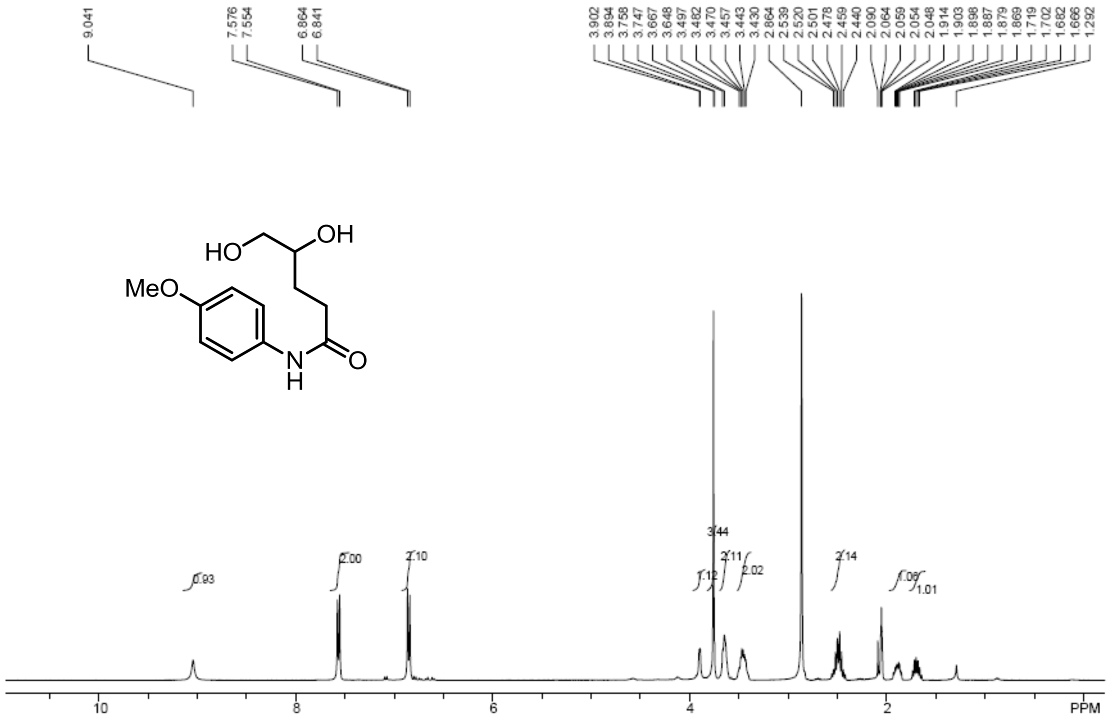

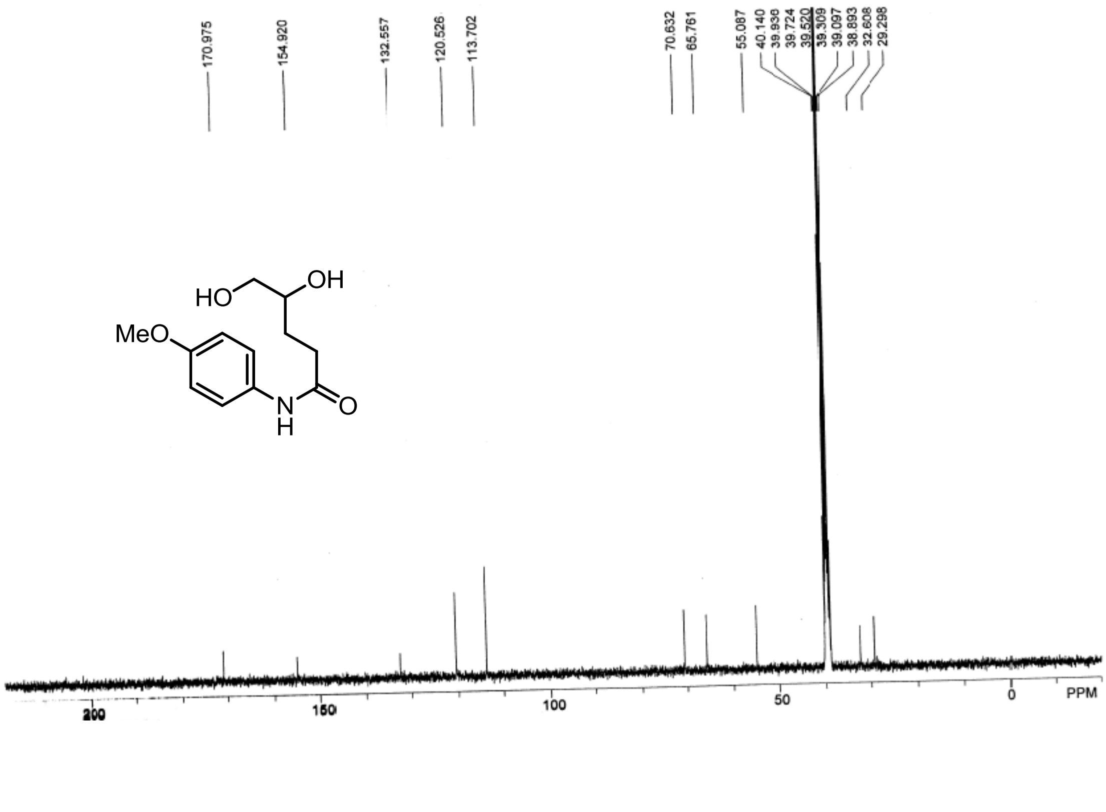

N-([1,1'-Biphenyl]-2-yl)-2-allylpent-4-enamide (**14**)

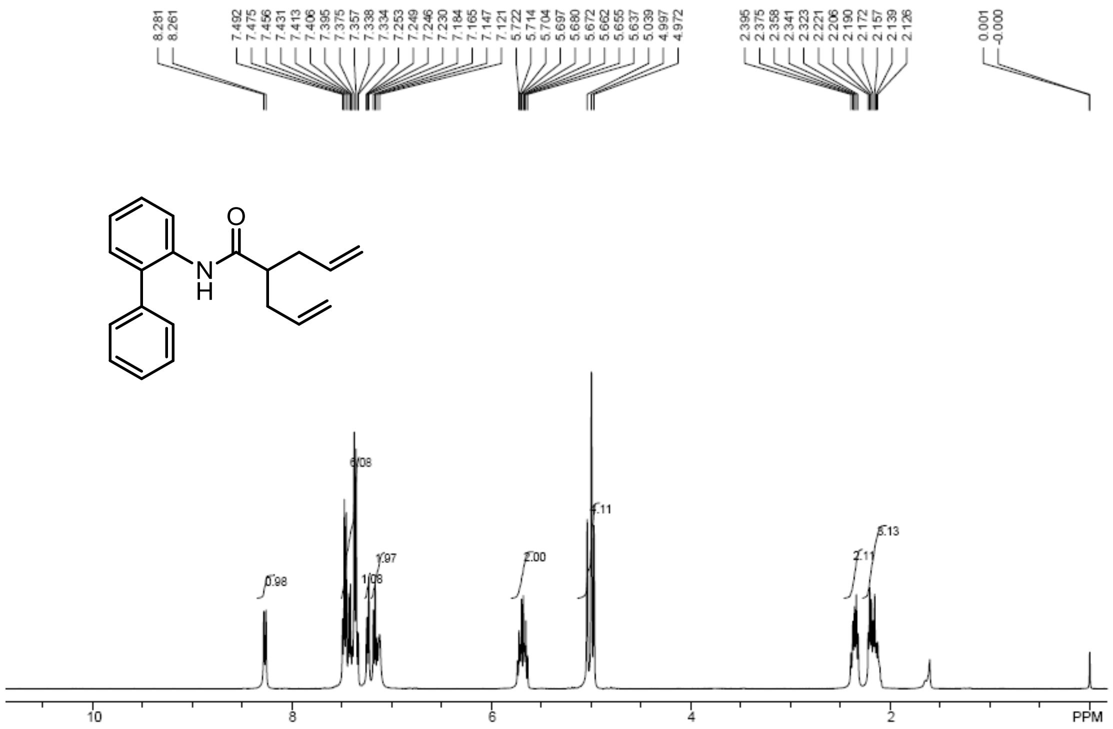

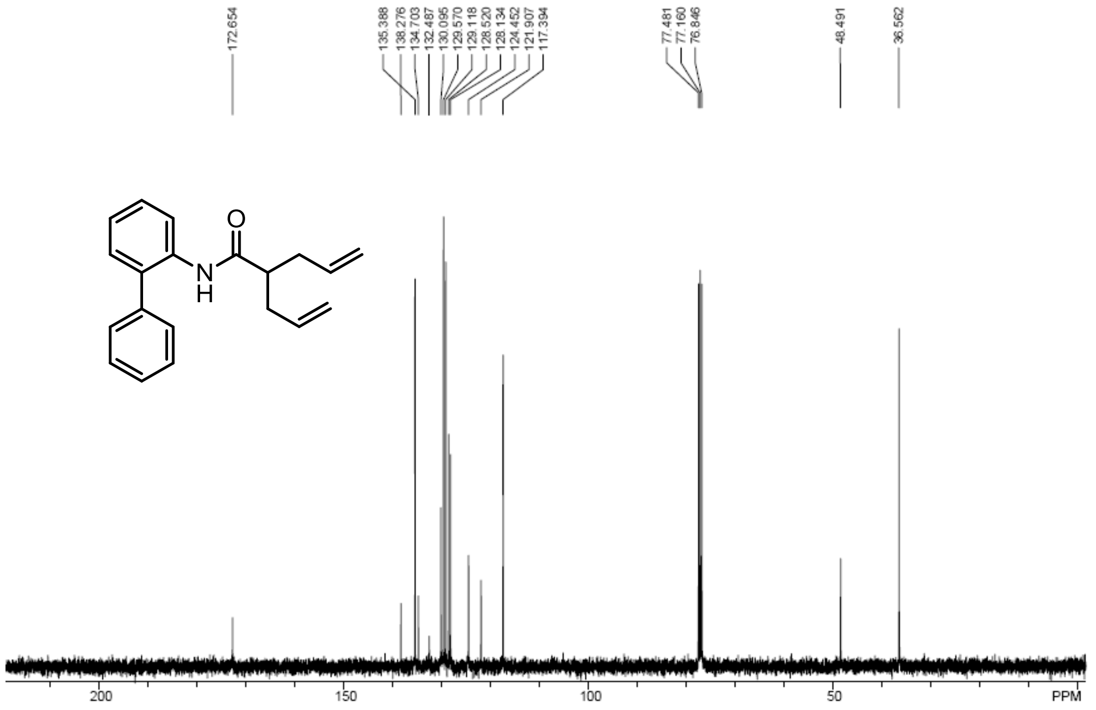

(*cis*-5-([1,1'-Biphenyl]-2-ylimino)-4-allyltetrahydrofuran-2-yl)methanol (***cis*-iminolactone C**)

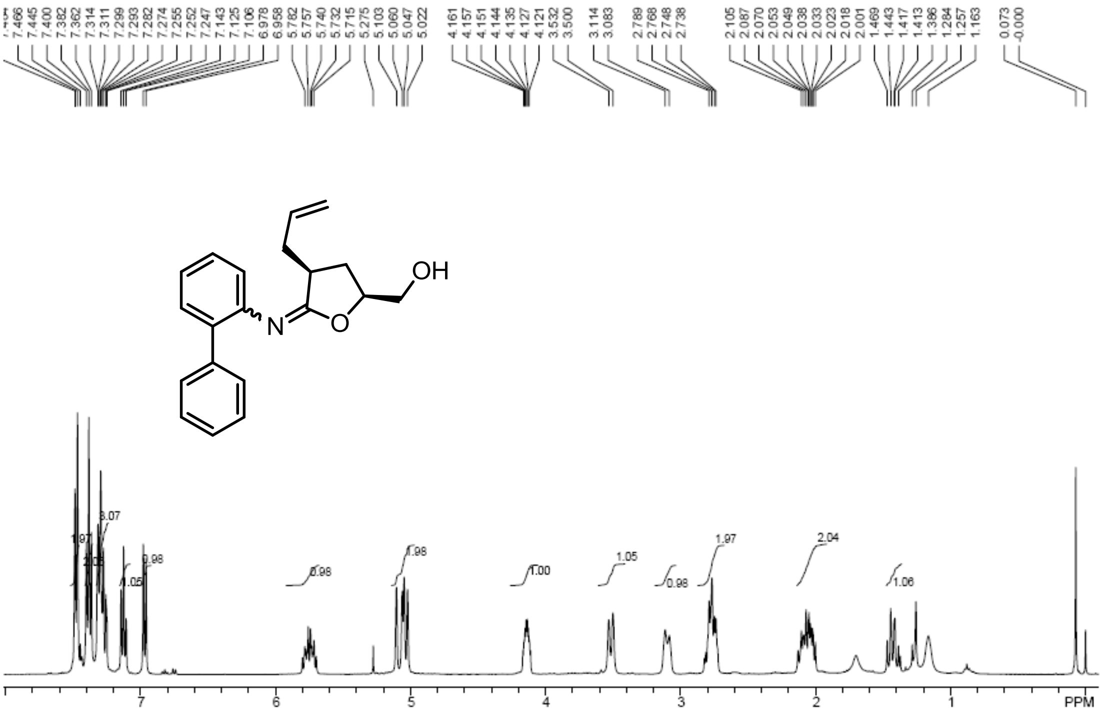

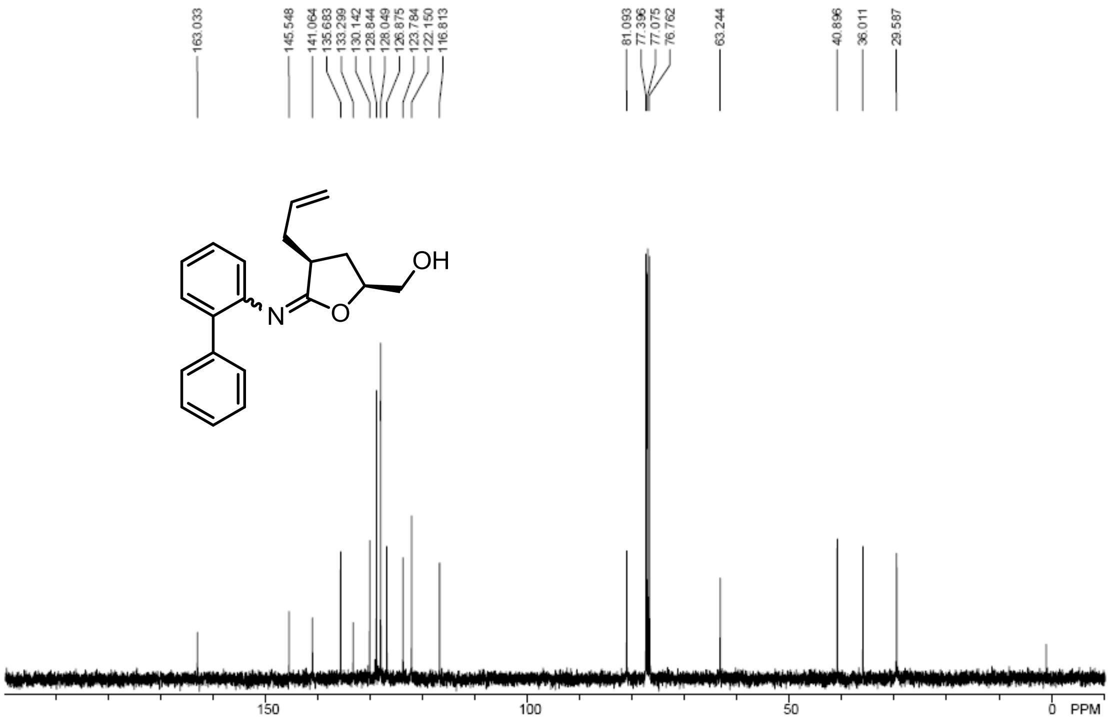

(*trans*-5-([1,1'-Biphenyl]-2-ylimino)-4-allyltetrahydrofuran-2-yl)methanol (***trans*-iminolacton C**)

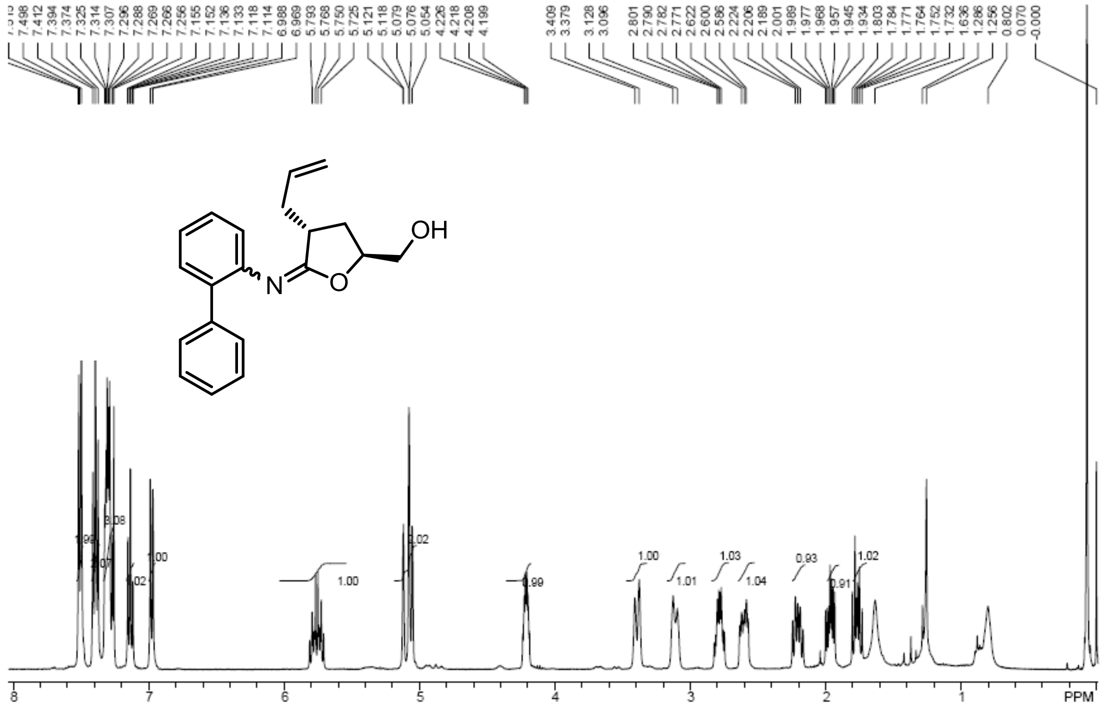

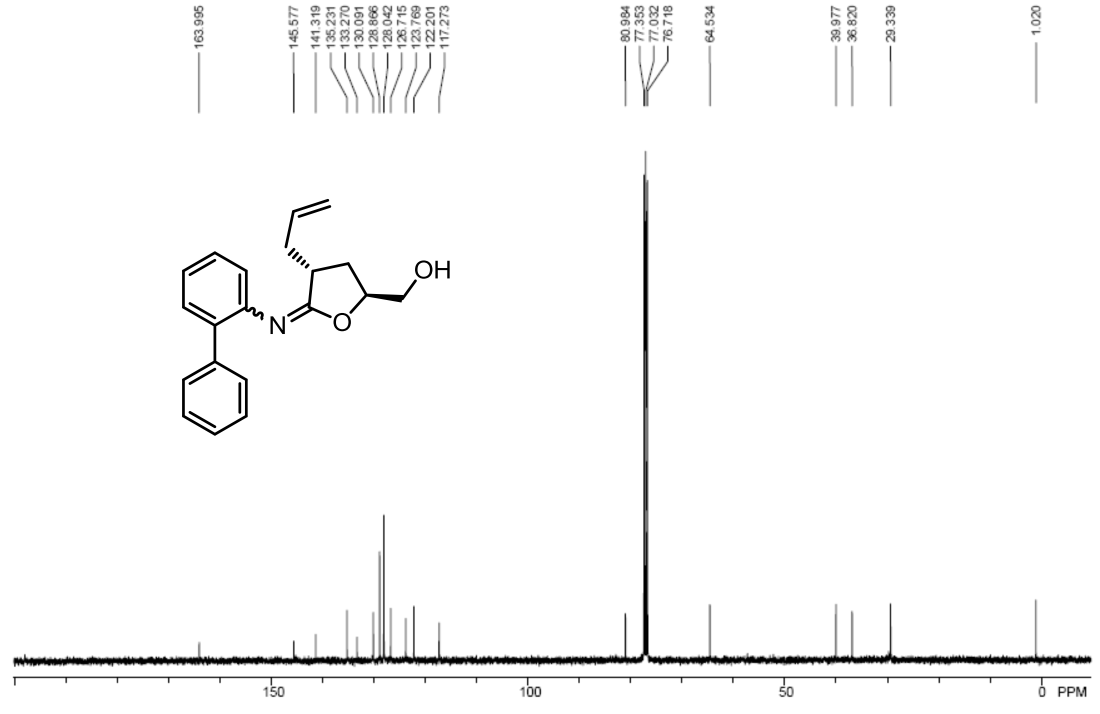

N-(3-Allyl-5-((benzyloxy)methyl)dihydrofuran-2(3*H*)-ylidene)-[1,1'-biphenyl]-2-amine (**C**)

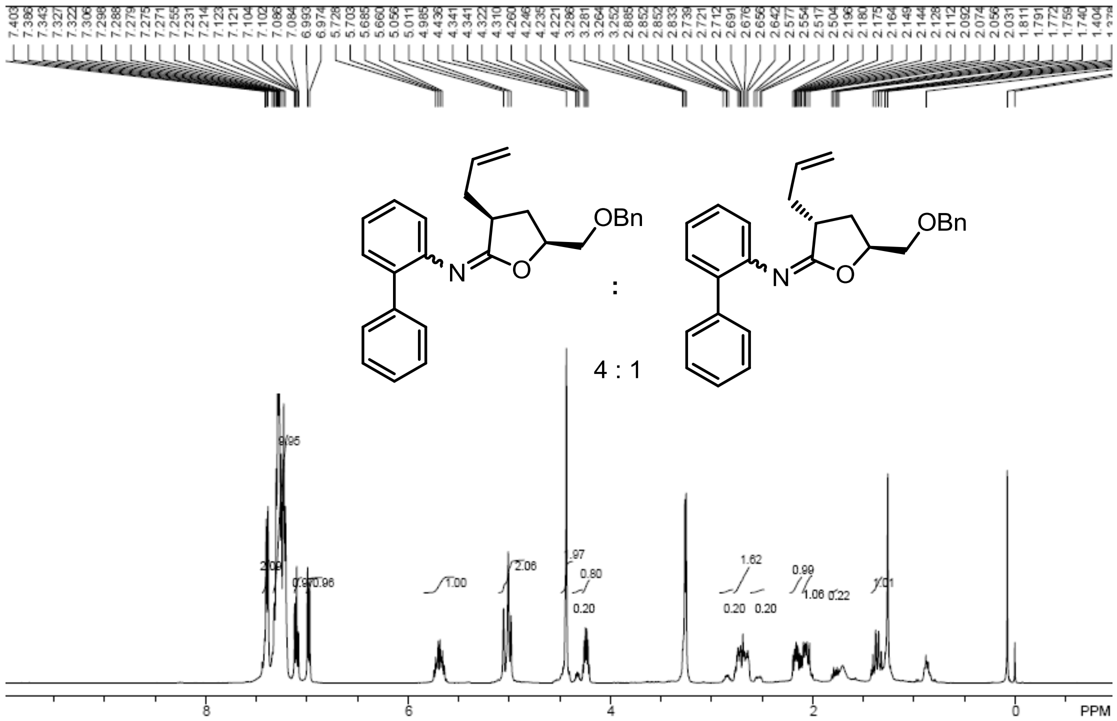

5-(Hydroxymethyl)dihydrofuran-2(3*H*)-one (**15**)

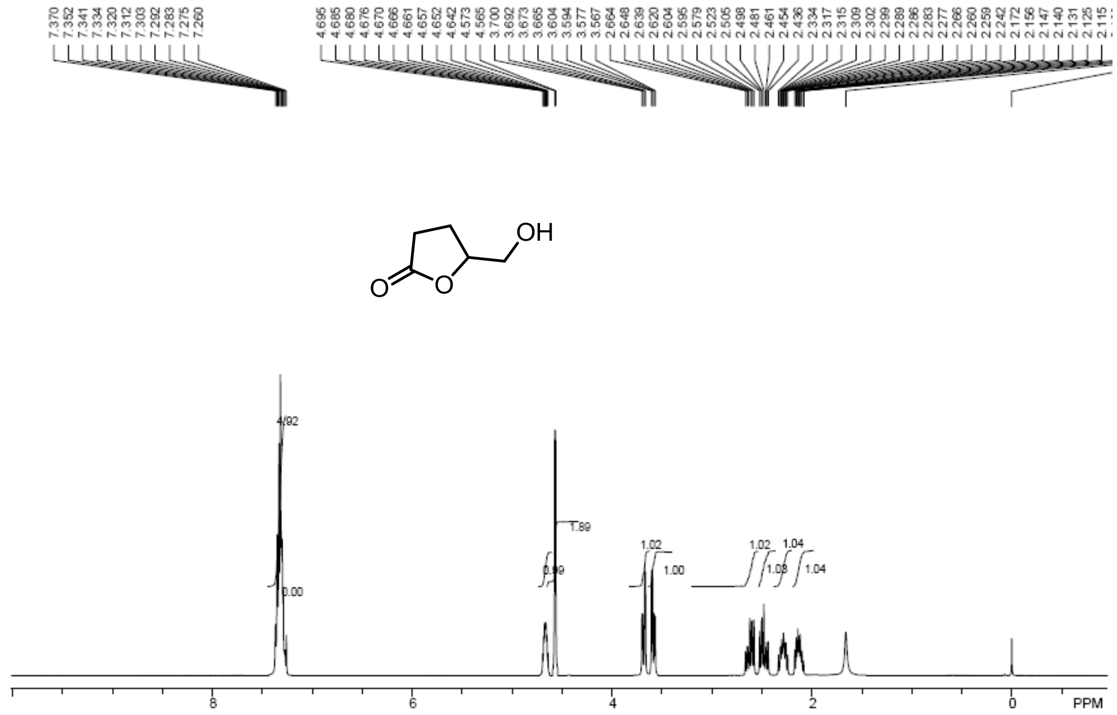

cis-3-Allyl-5-((benzyloxy)methyl)dihydrofuran-2(3*H*)-one (*cis*-**17**)

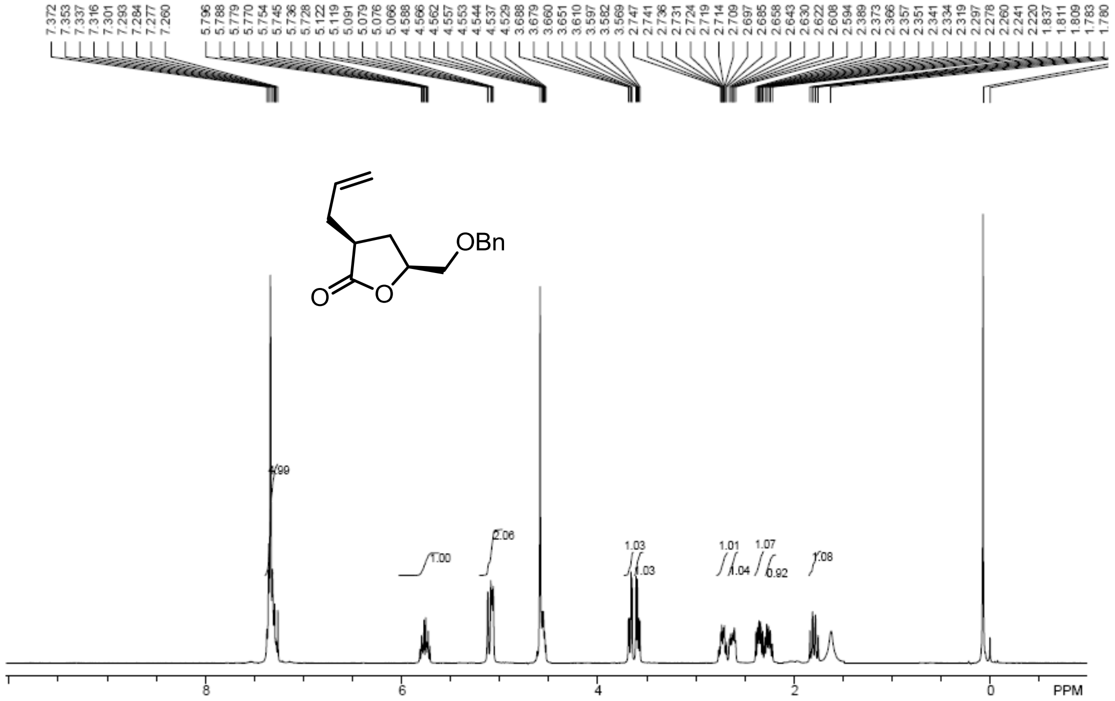

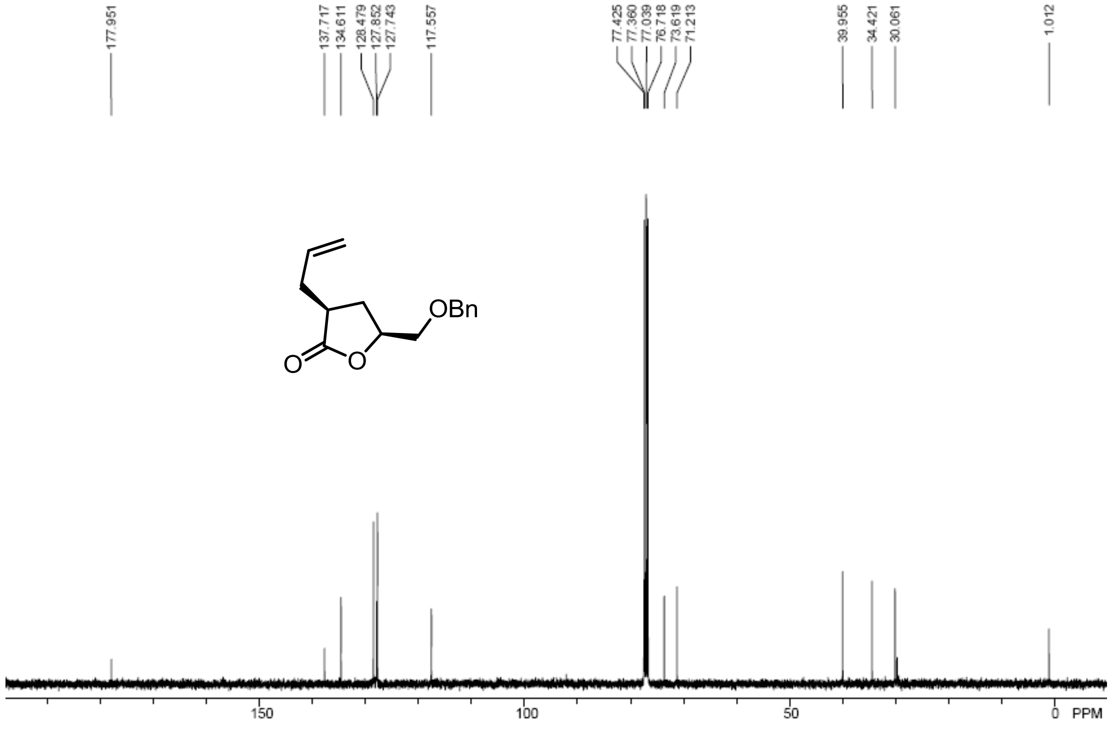

trans-3-Allyl-5-((benzyloxy)methyl)dihydrofuran-2(3H)-one (*trans*-**17**)

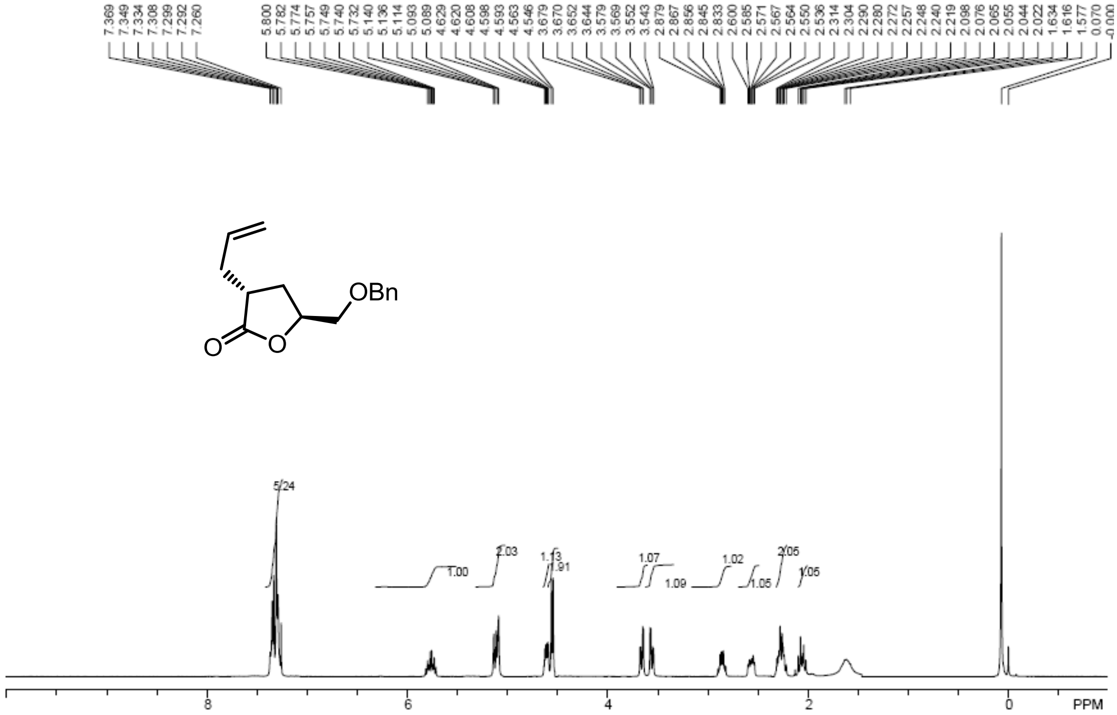

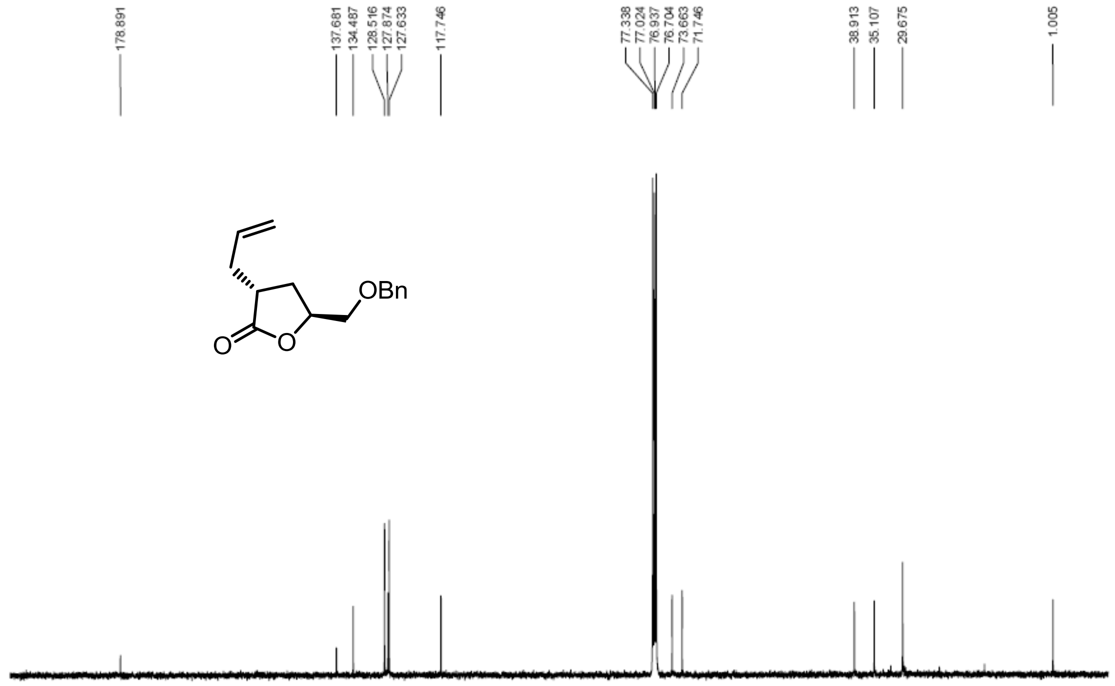

4,5-Dihydroxy-*N*-(*p*-tolyl)pentanamide (**19a**)

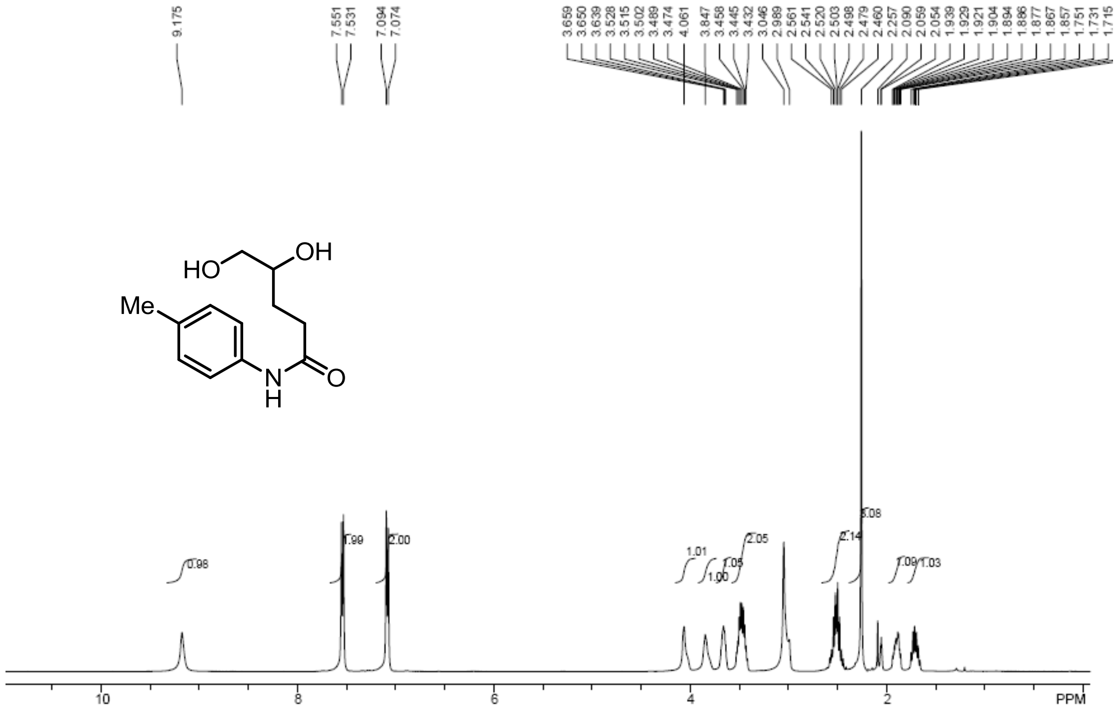

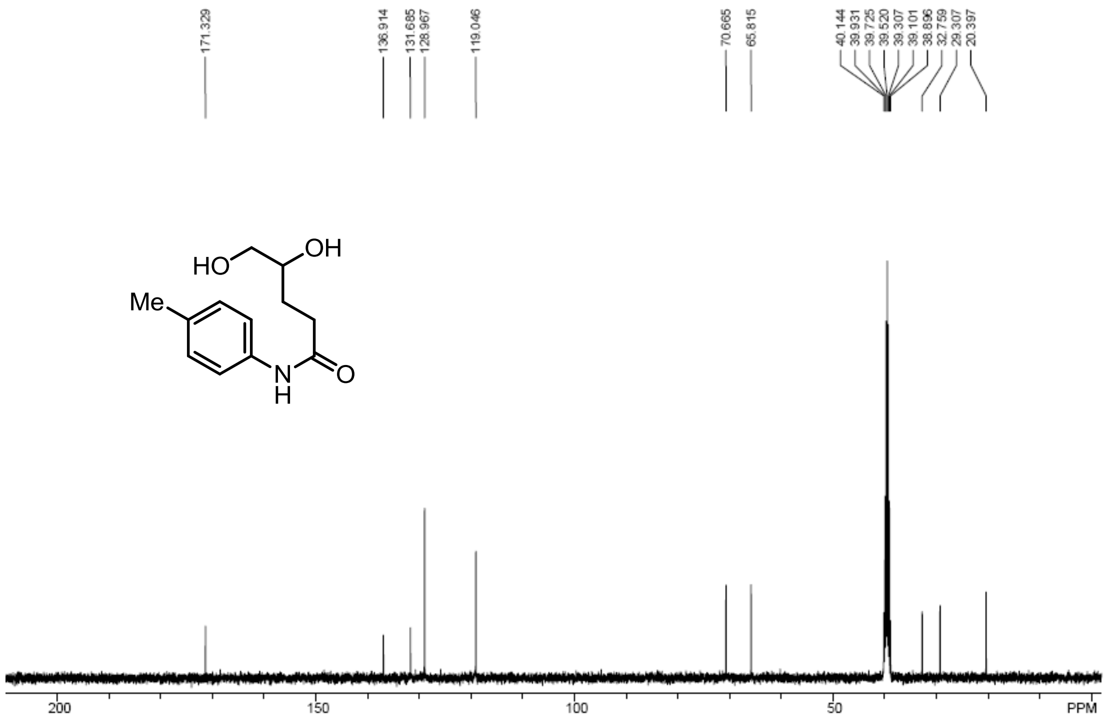

4,5-Dihydroxy-*N*-phenylpentanamide (**19b**)

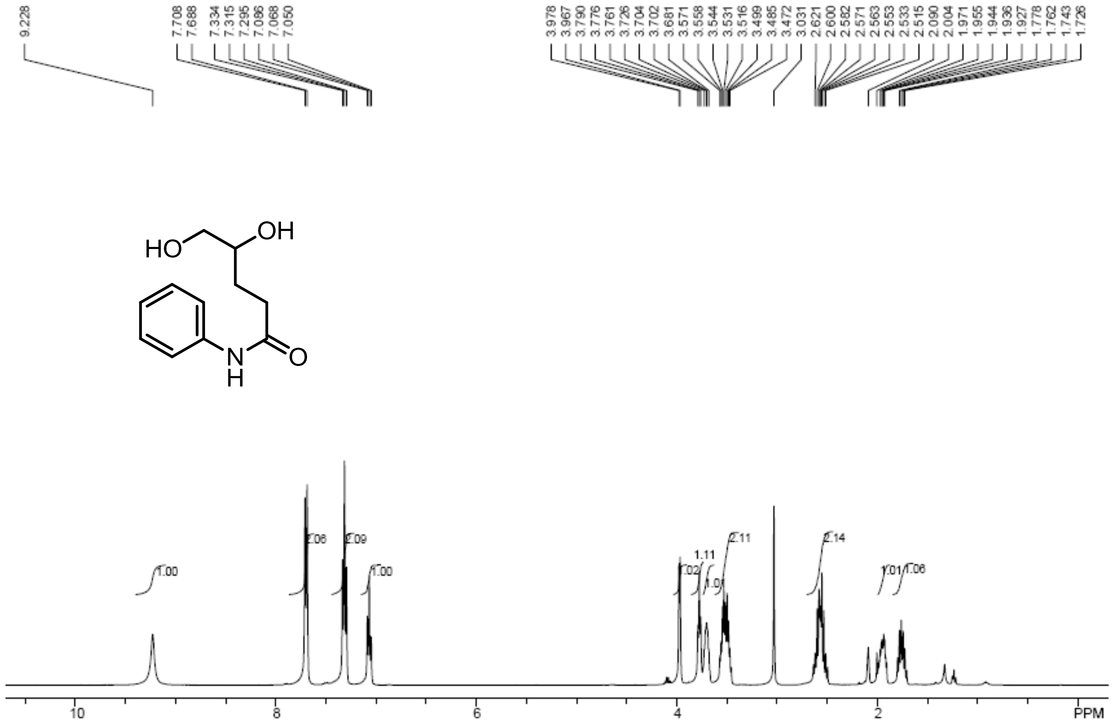

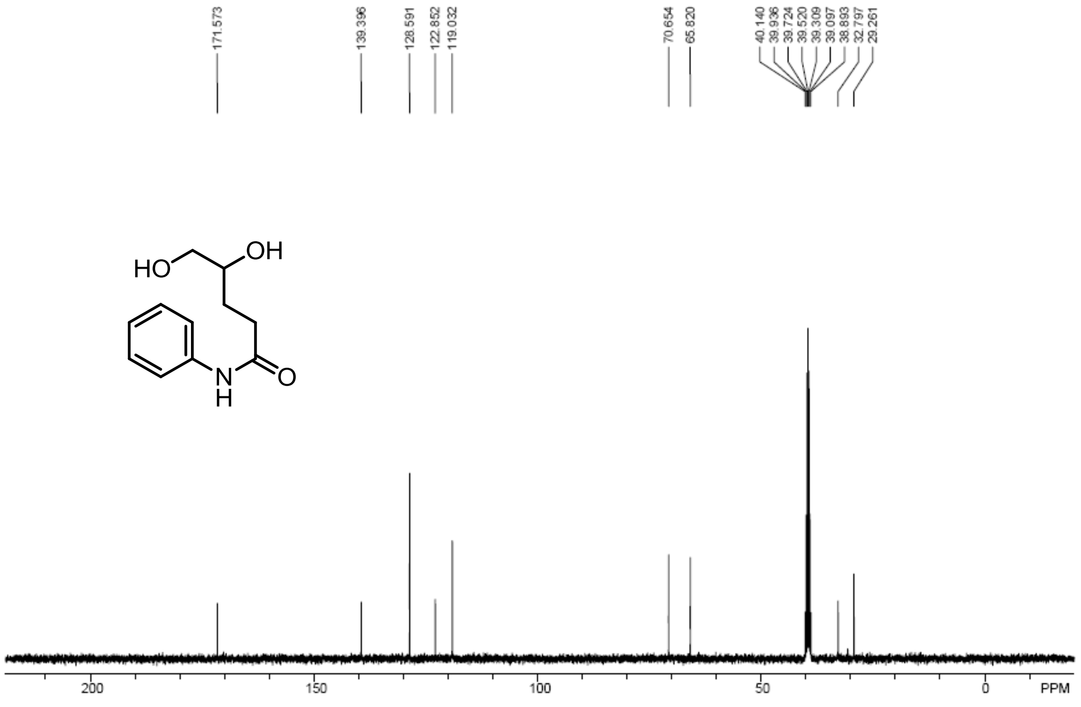

N-(4-Chlorophenyl)pent-4-enamide (**19c**)

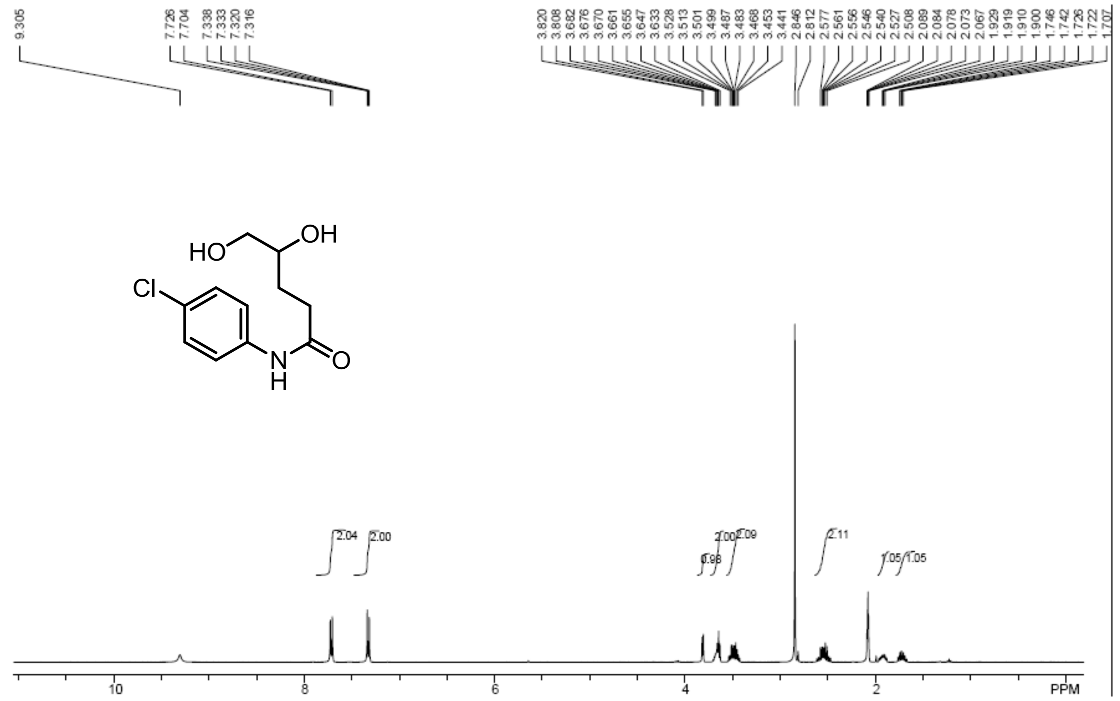

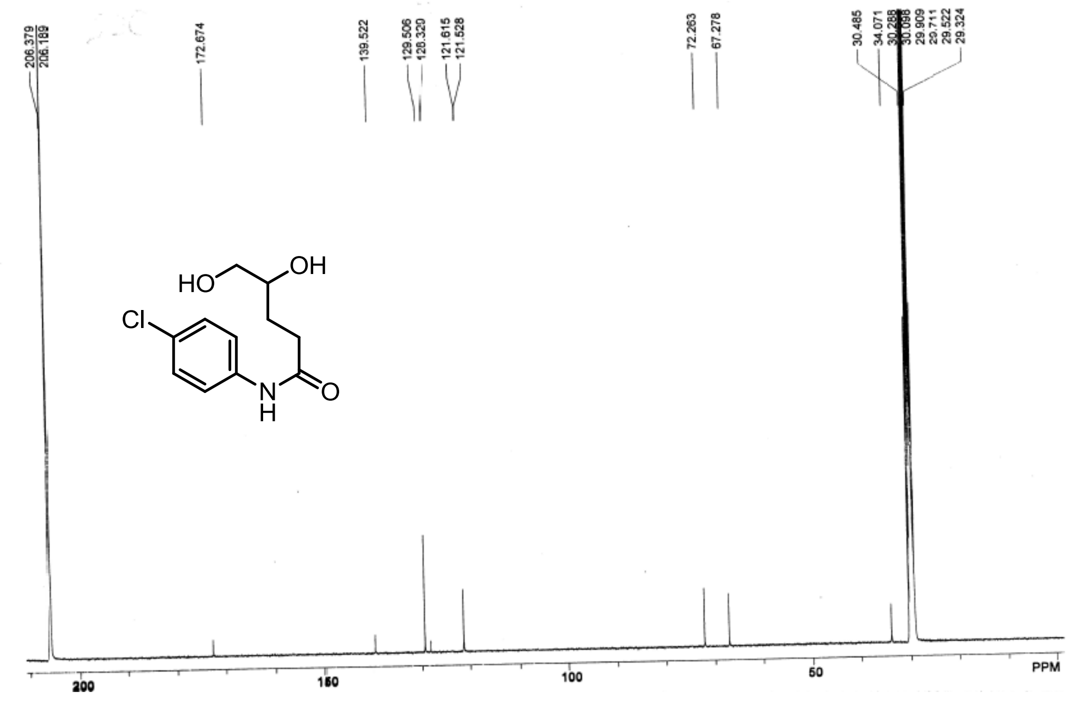

4,5-Dihydroxy-4-methyl-*N*-(*p*-tolyl)pentanamide (**19d**)

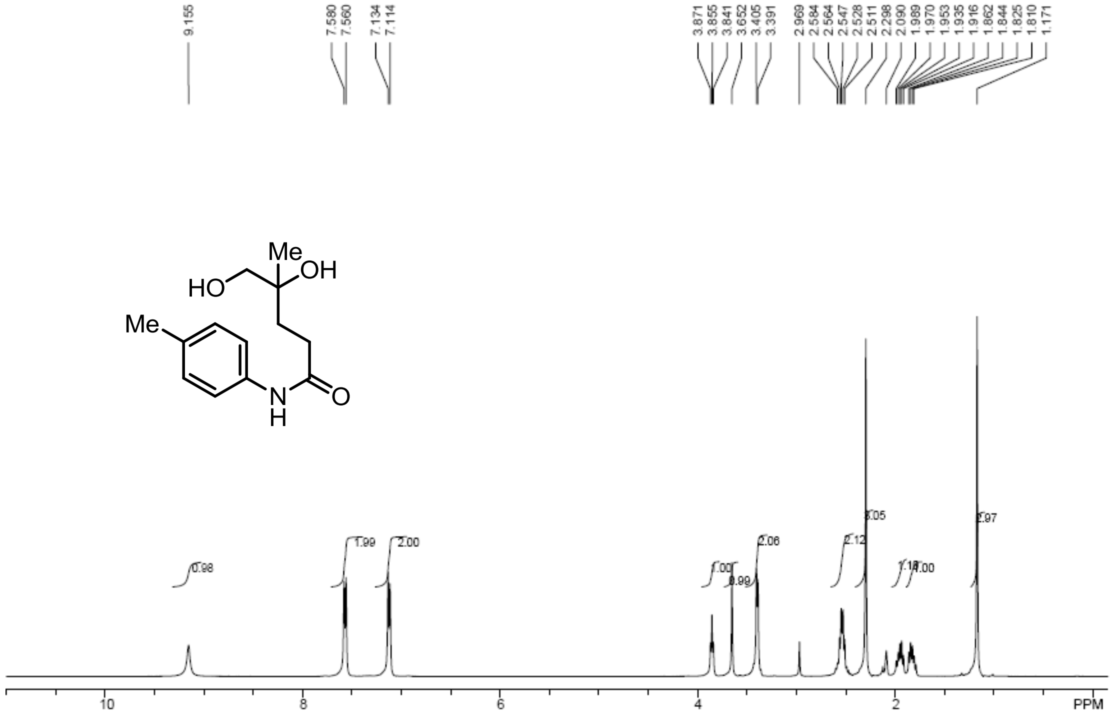

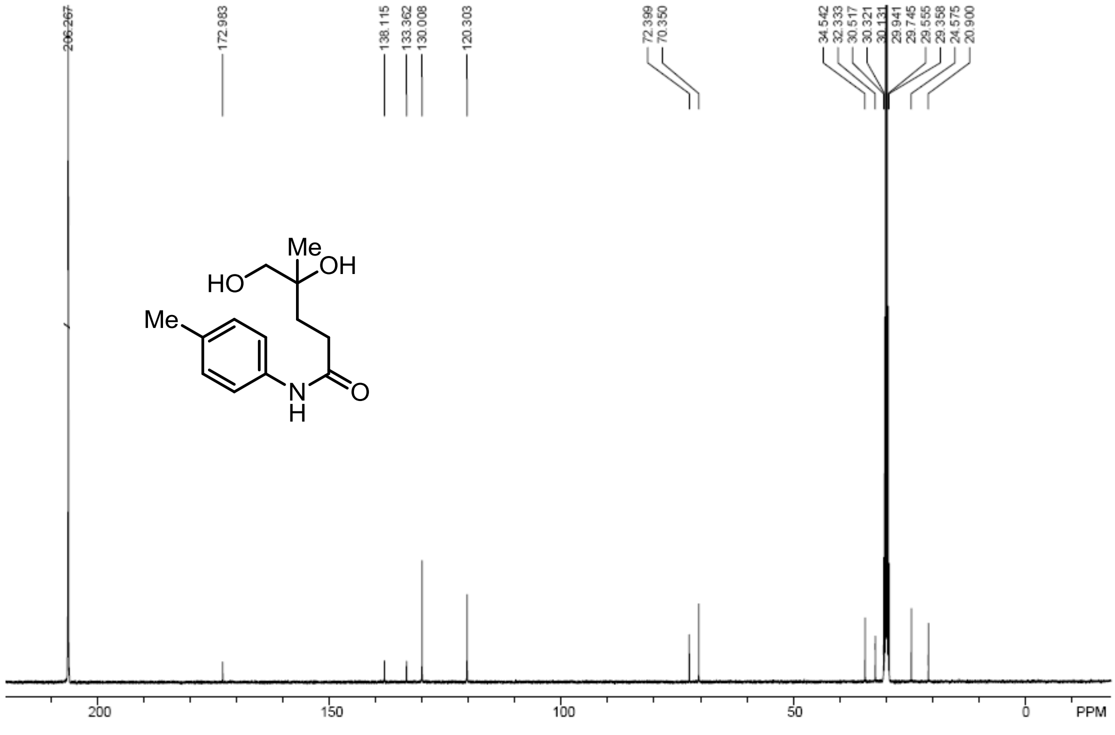

4,5-Dihydroxy-2-methyl-*N*-(*p*-tolyl)pentanamide (**19e**)

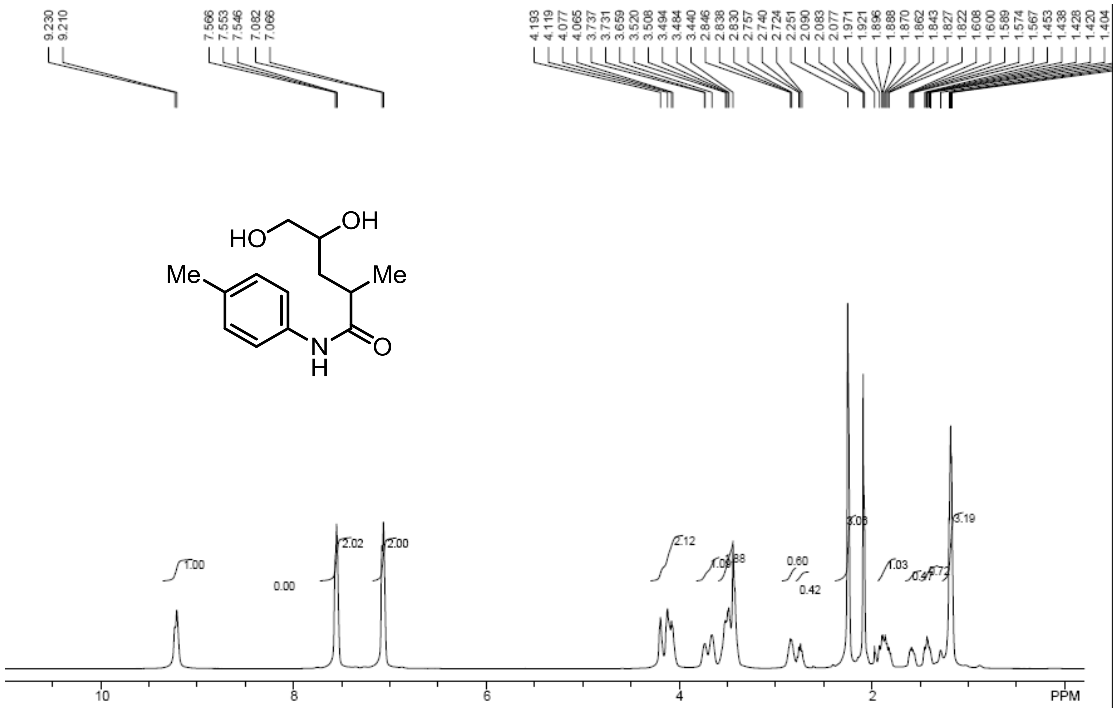

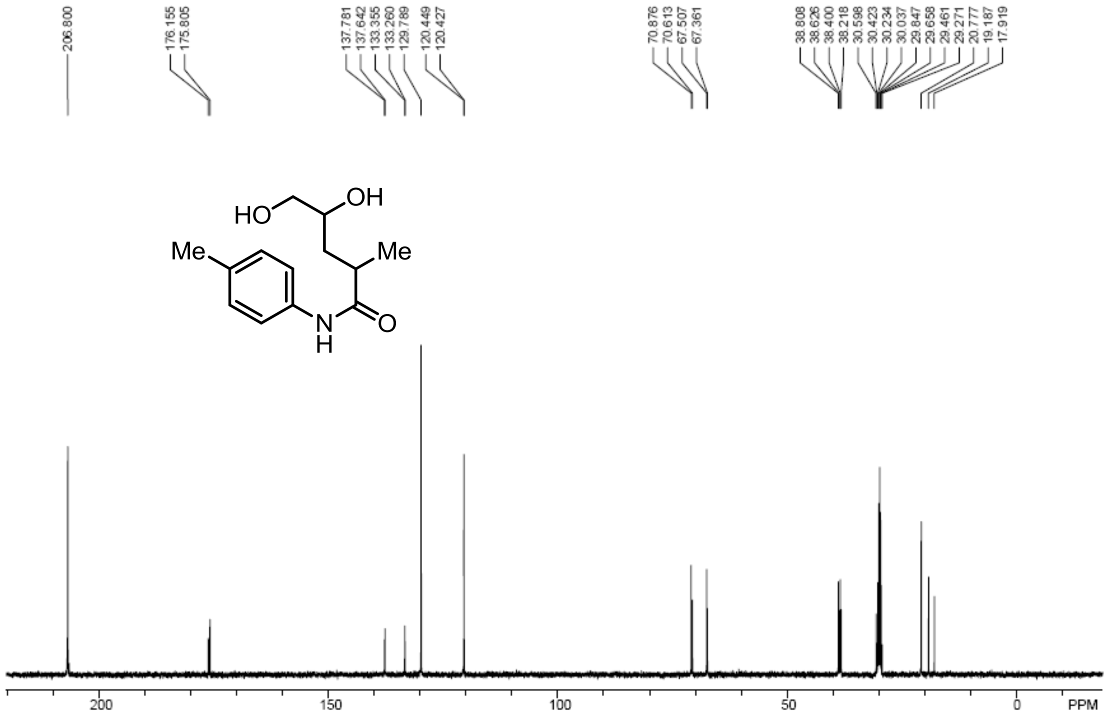

2-Allyl-*N*-(*p*-tolyl)pent-4-enamide (**20**)

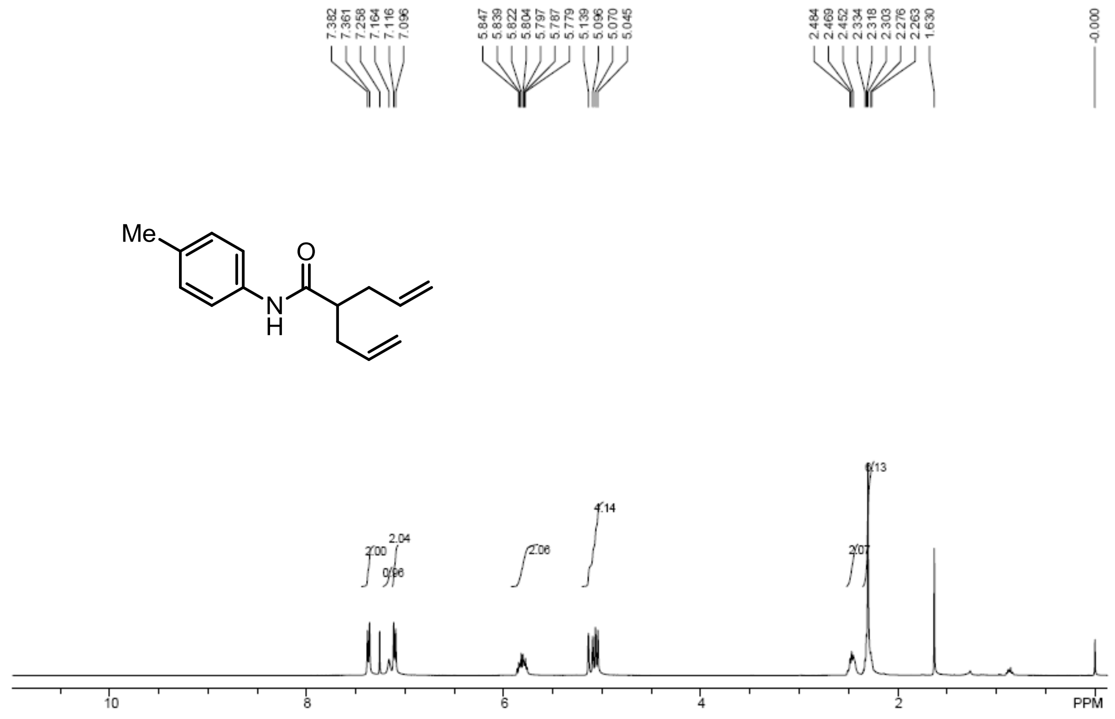

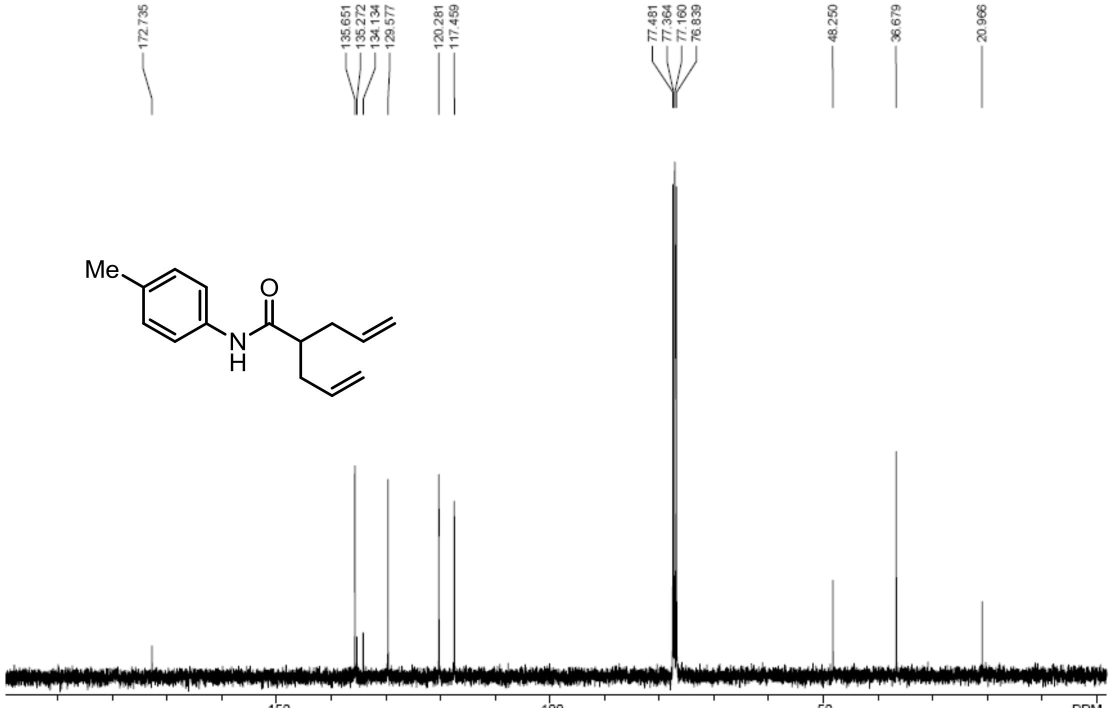

(*R*)-2-(((*R*)-2,2-Dimethyl-1,3-dioxolan-4-yl)methyl)-*N*-(*p*-tolyl)pent-4-enamide (**22a**)

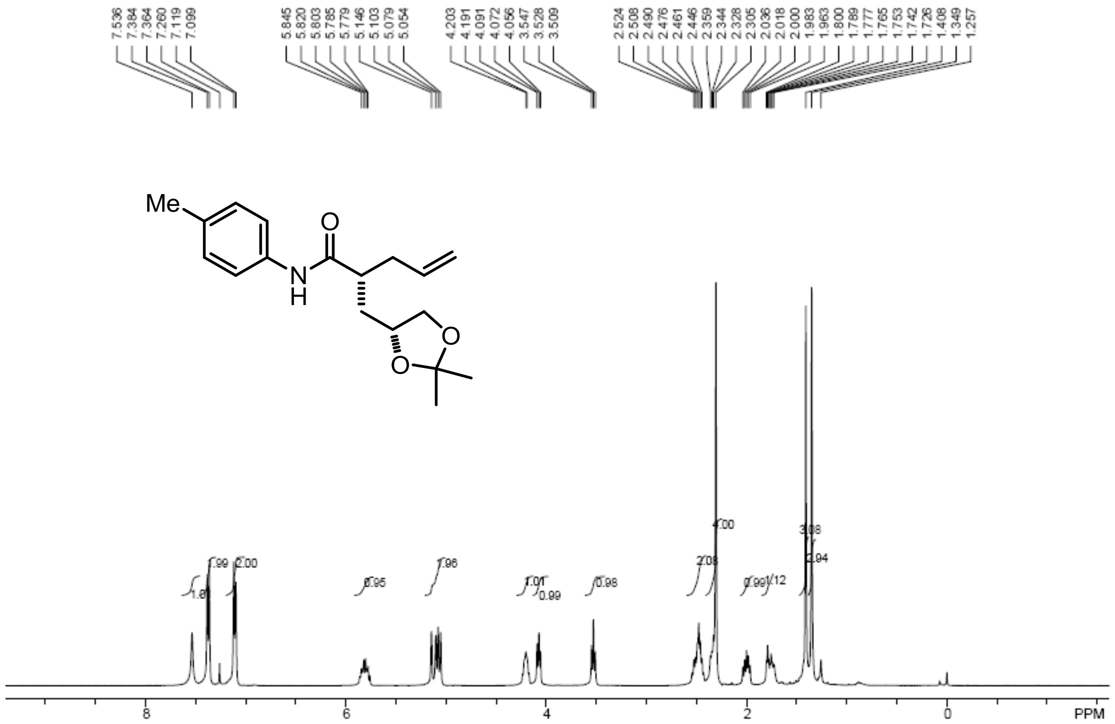

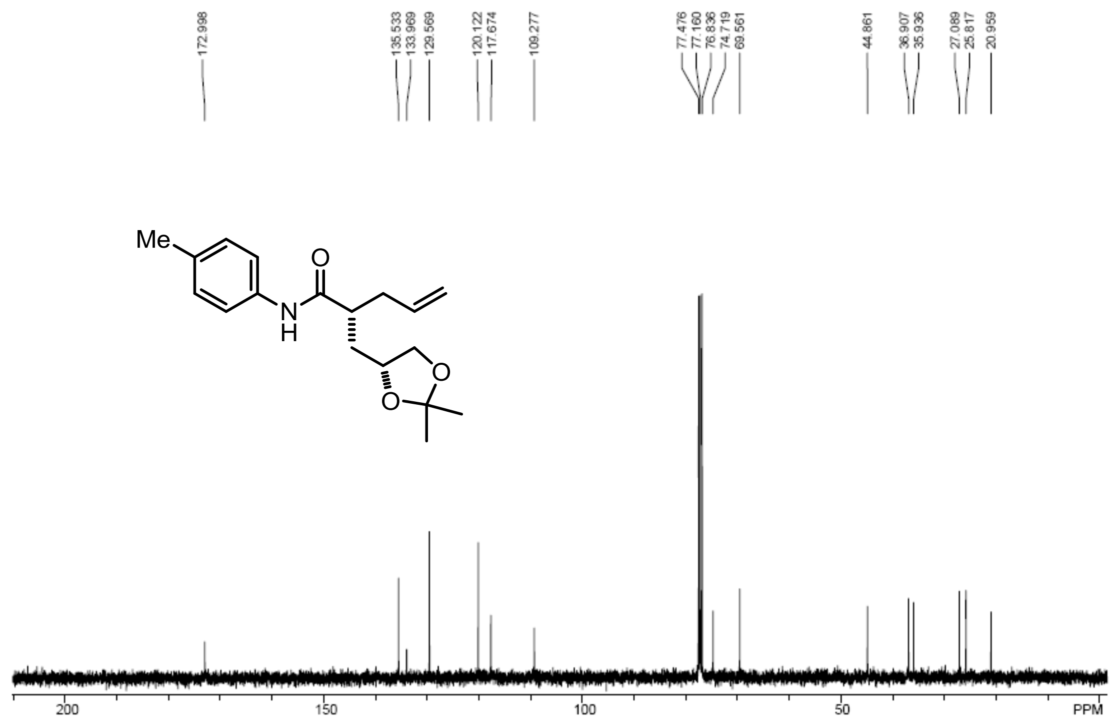

(*S*)-2-(((*R*)-2,2-Dimethyl-1,3-dioxolan-4-yl)methyl)-*N*-(*p*-tolyl)pent-4-enamide (**22b**)

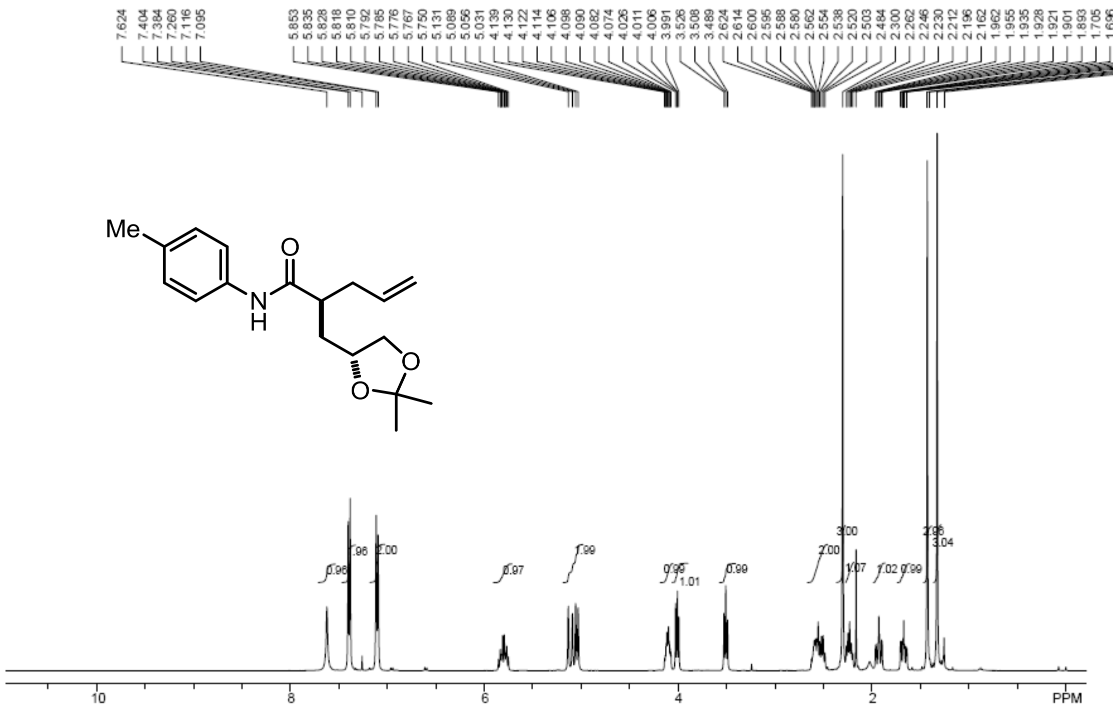

(2*R*)-2-(((*R*)-2,2-Dimethyl-1,3-dioxolan-4-yl)methyl)-4,5-dihydroxy-*N*-(*p*-tolyl)pentanamide (**23**)

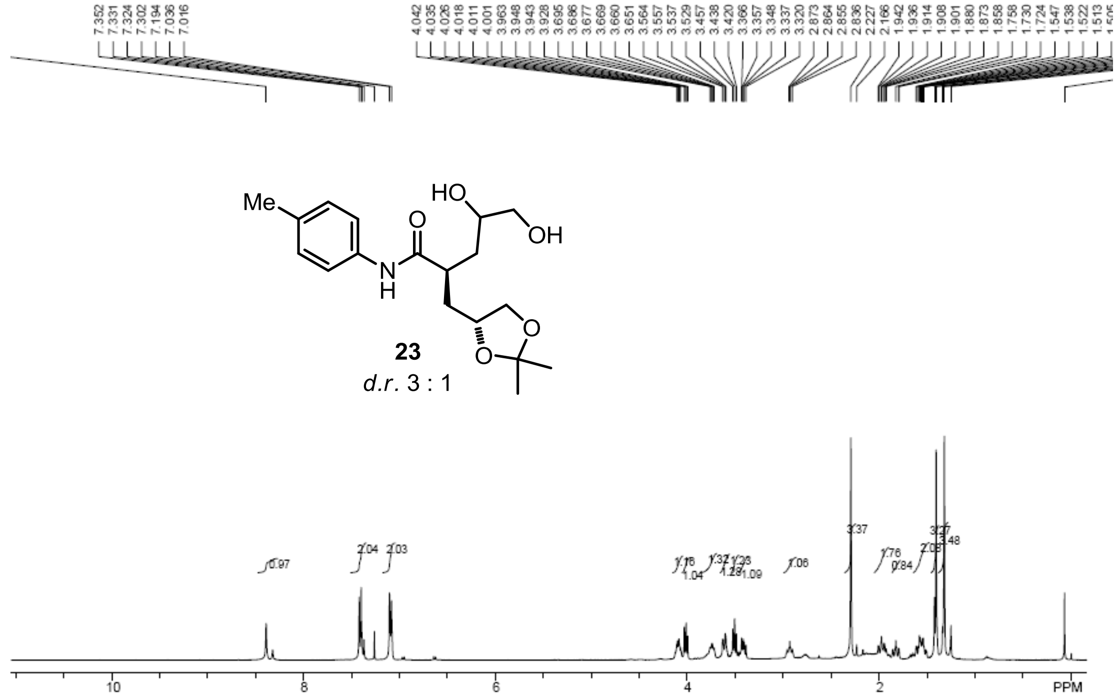

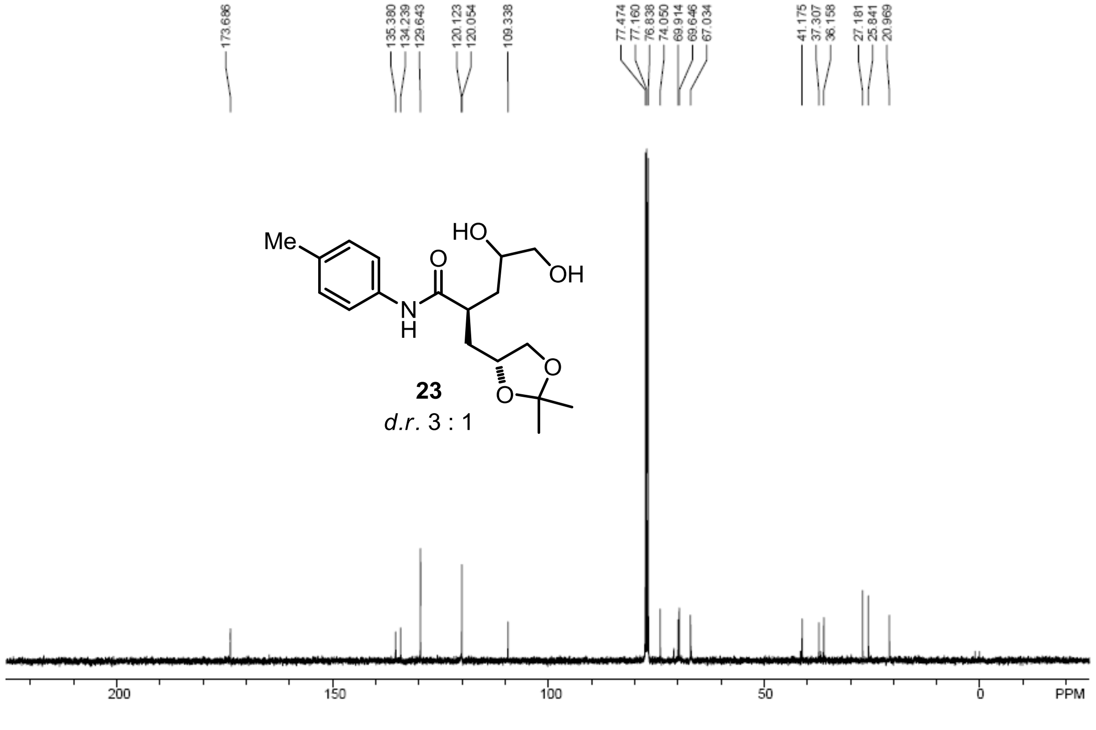

2-Benzyl-*N*-(*p*-tolyl)pent-4-enamide (**24a**)

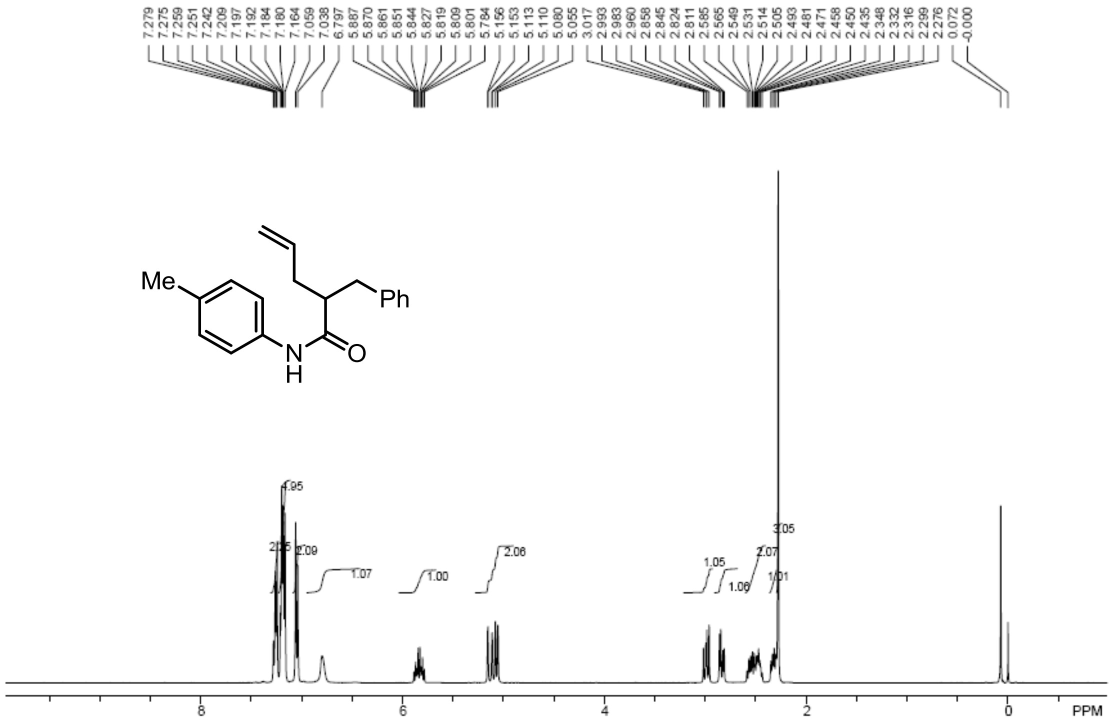

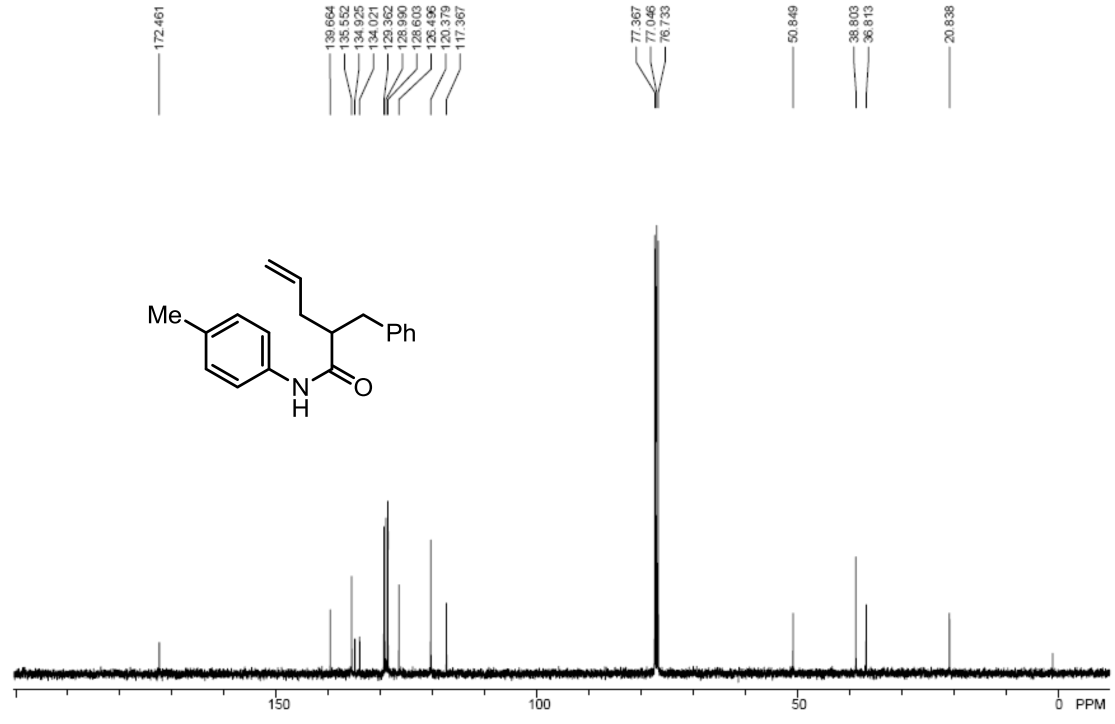

3-Benzyl-5-(hydroxymethyl)dihydrofuran-2(3*H*)-one (**25a**)

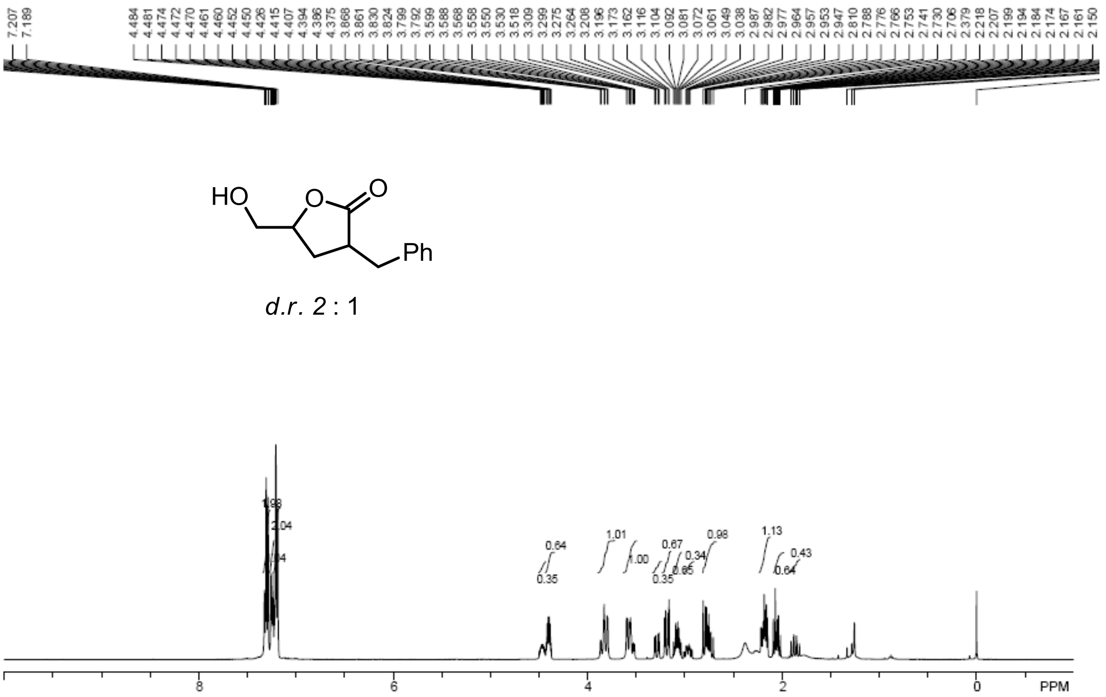

(*E*)-2-Allyl-*N*-(*p*-tolyl)hex-4-enamide (**24b**)

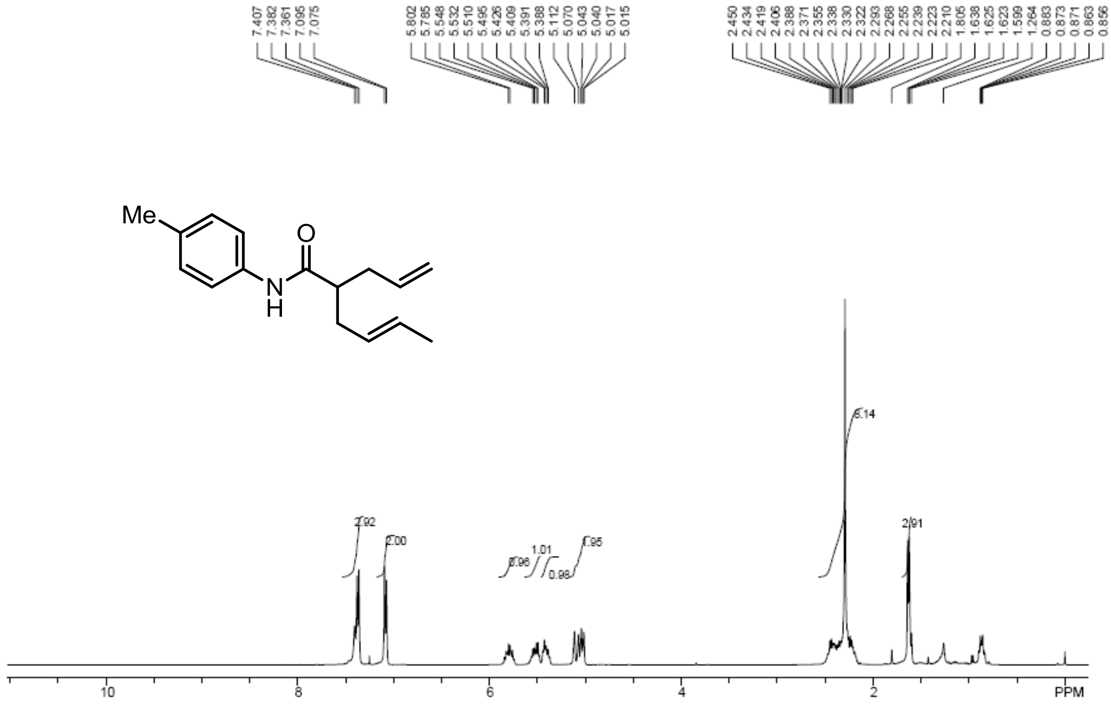

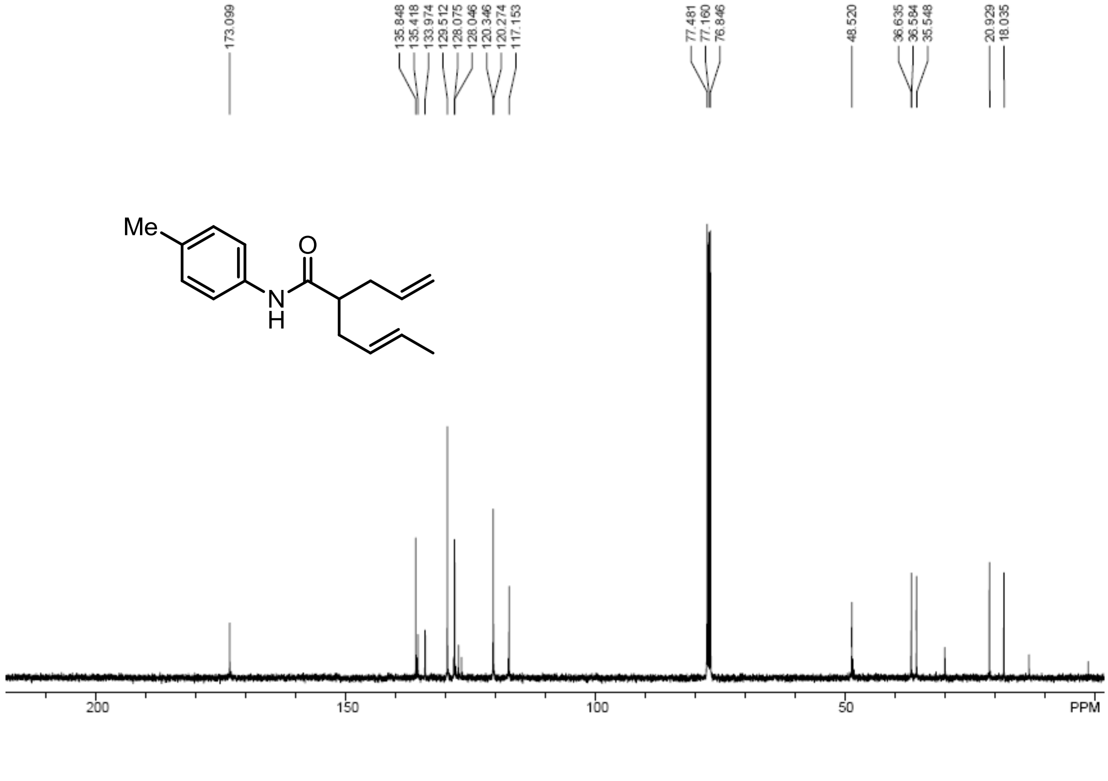

(*E*)-3-(But-2-en-1-yl)-5-(hydroxymethyl)dihydrofuran-2(3*H*)-one (**25b**)

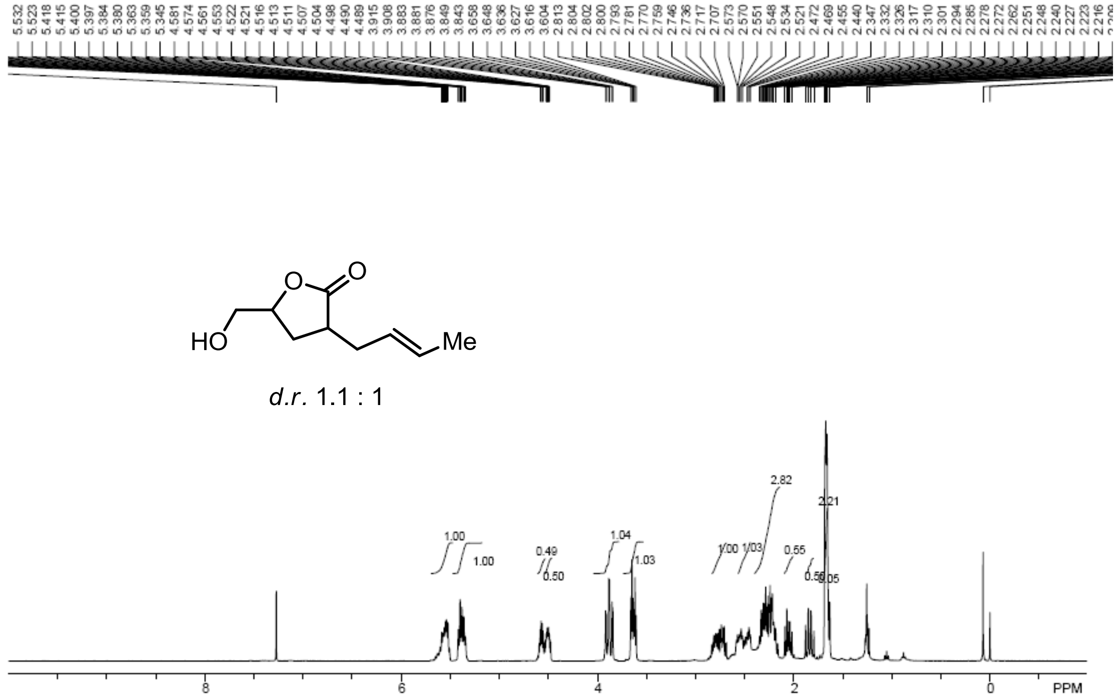

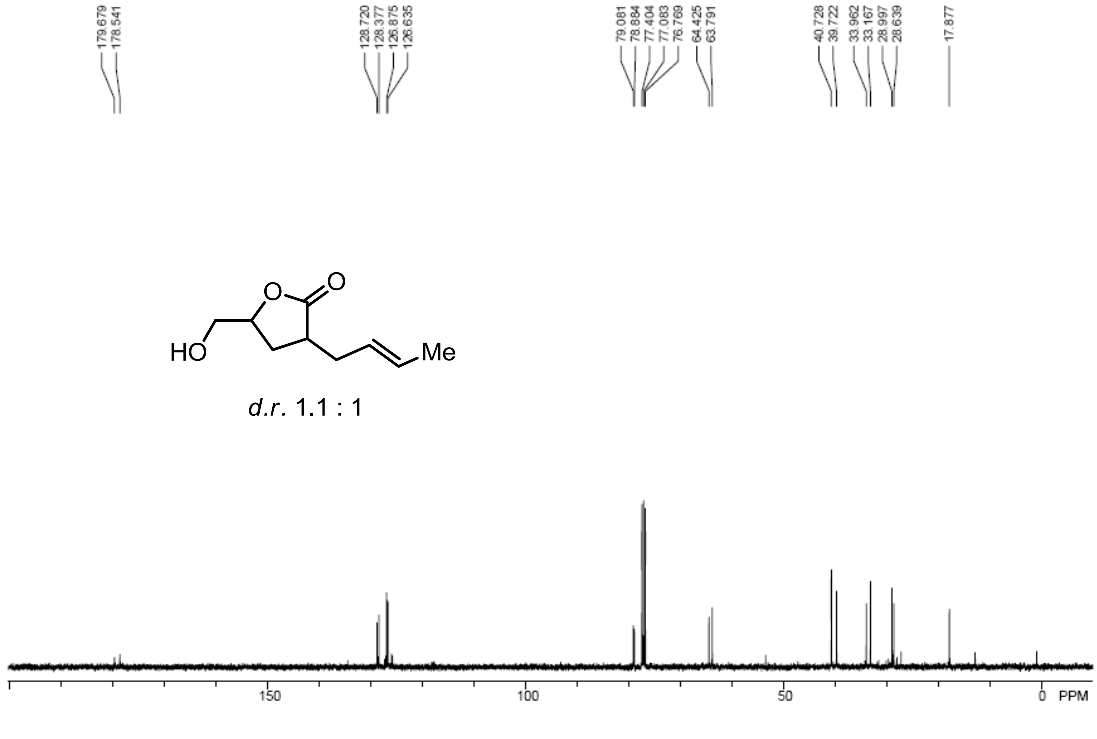

2-(Cyclopropylmethyl)-*N*-(*p*-tolyl)pent-4-enamide (**24e**)

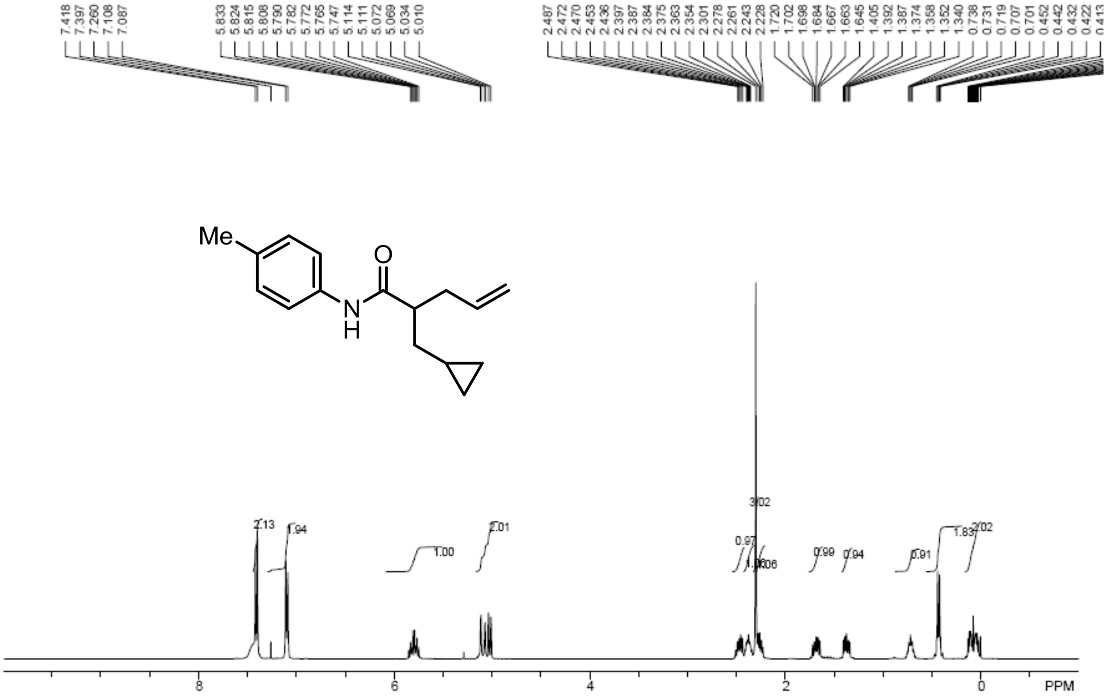

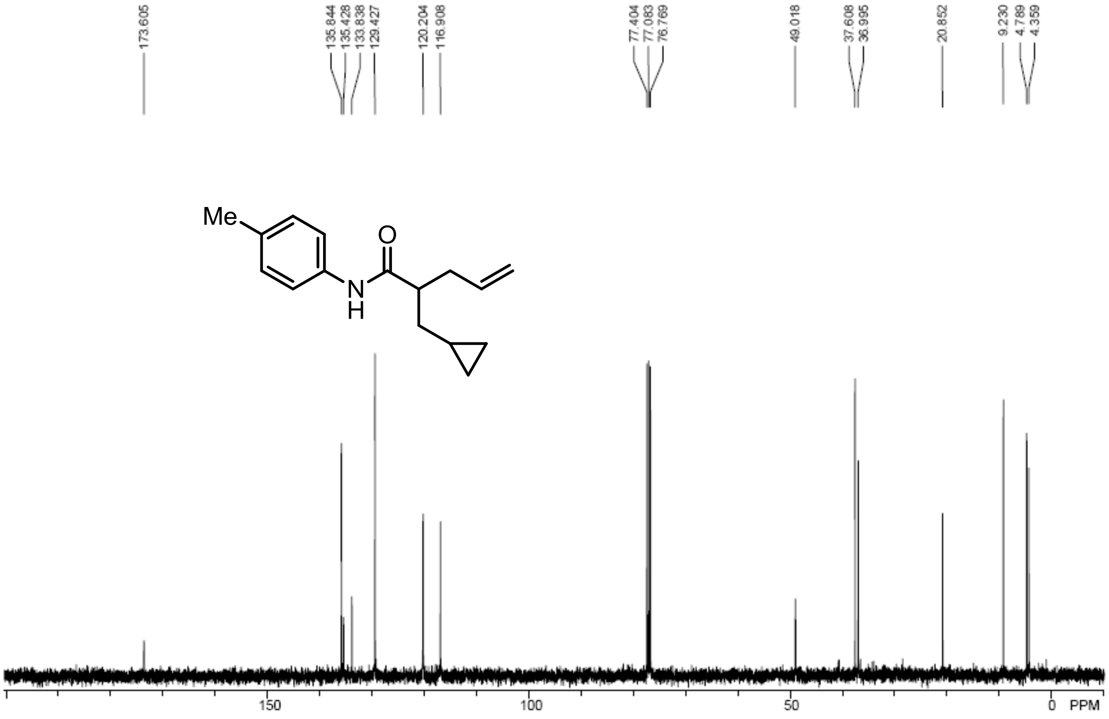

3-(Cyclopropylmethyl)-5-(hydroxymethyl)dihydrofuran-2(3*H*)-one (**25e**)

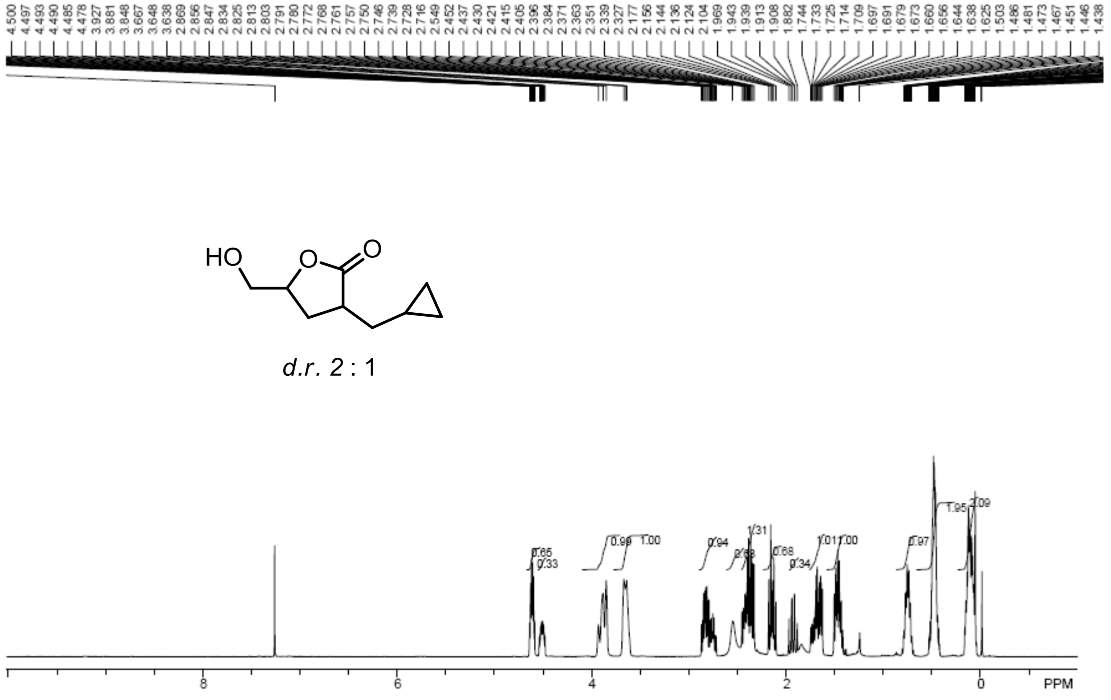

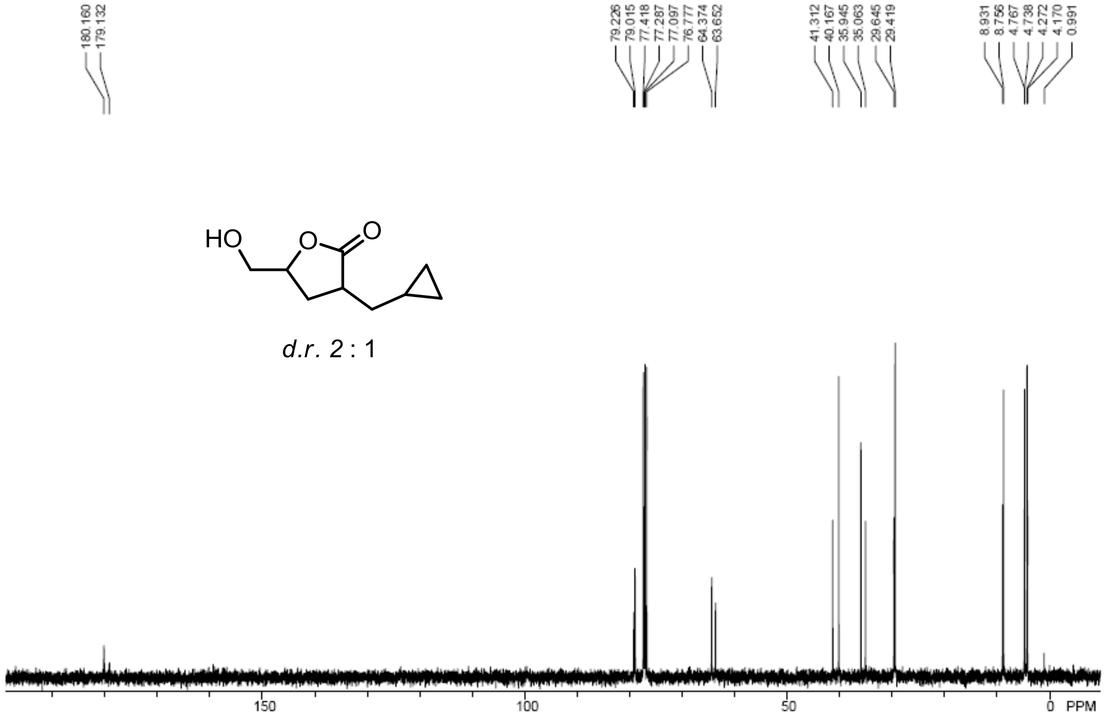

(*E*)-2-Allyl-5-phenyl-*N*-(*p*-tolyl)pent-4-enamide (**24d**)

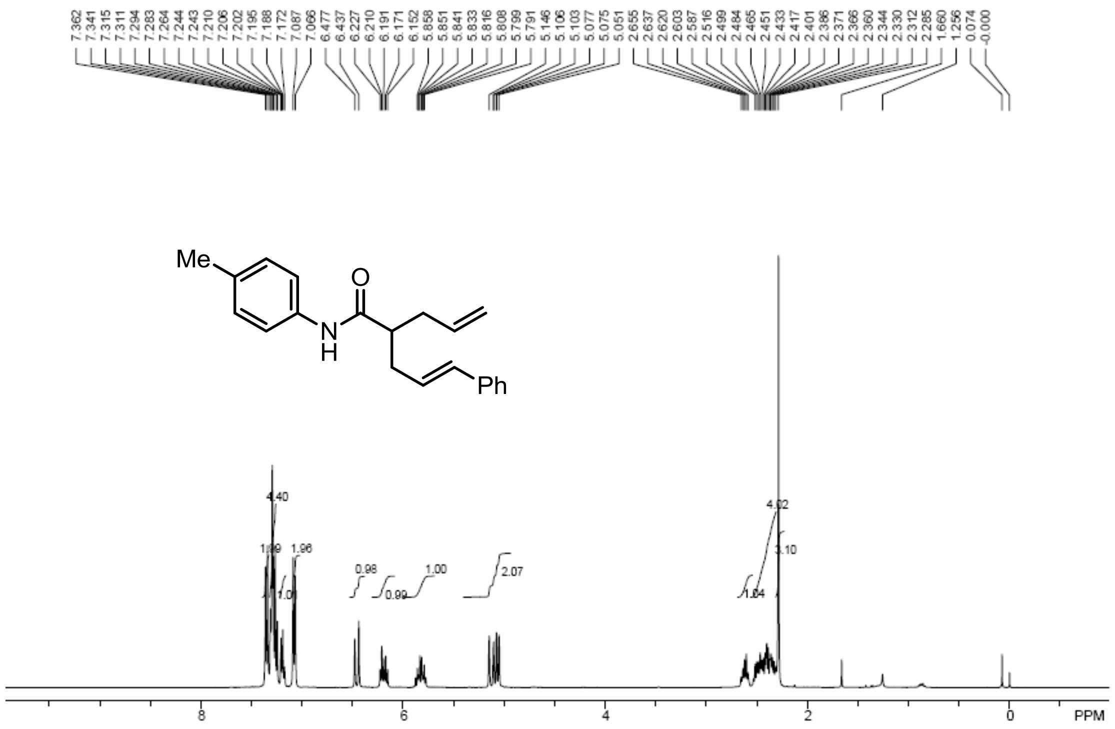

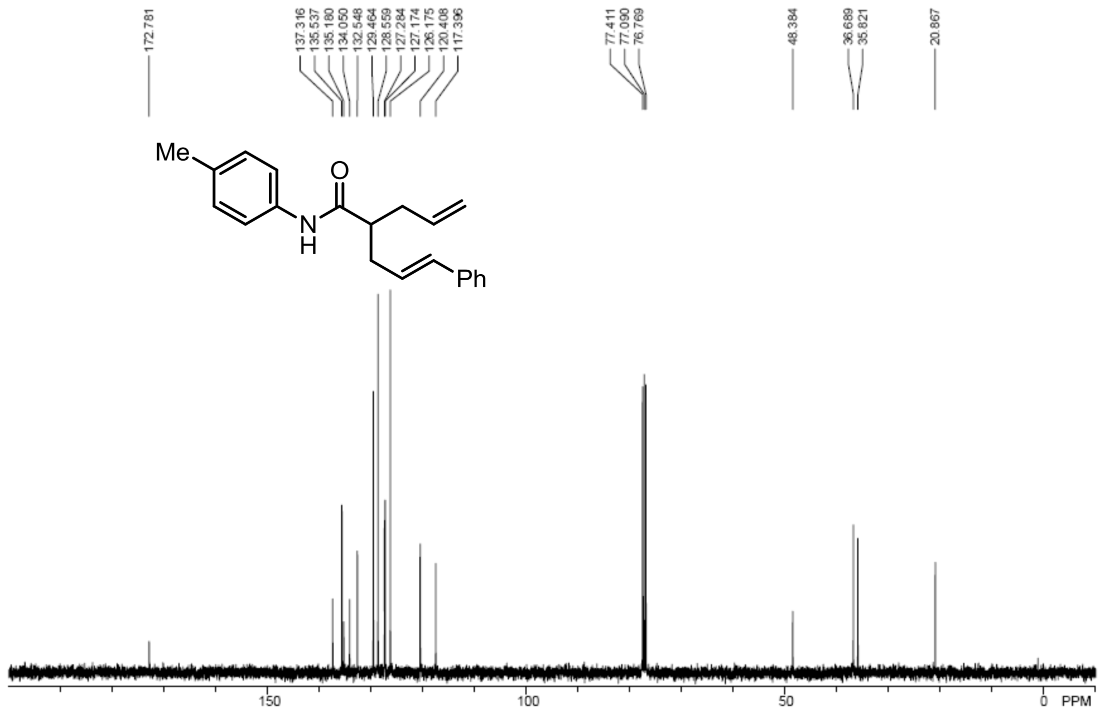

3-Cinnamyl-5-(hydroxymethyl)dihydrofuran-2(3*H*)-one (**25d**)

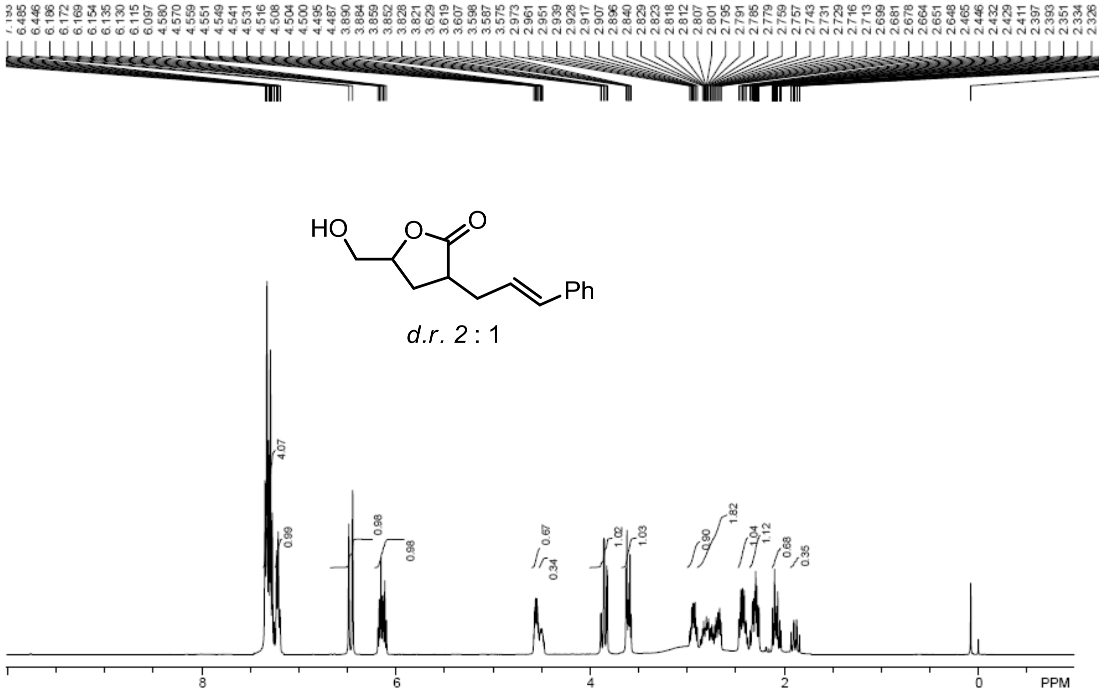

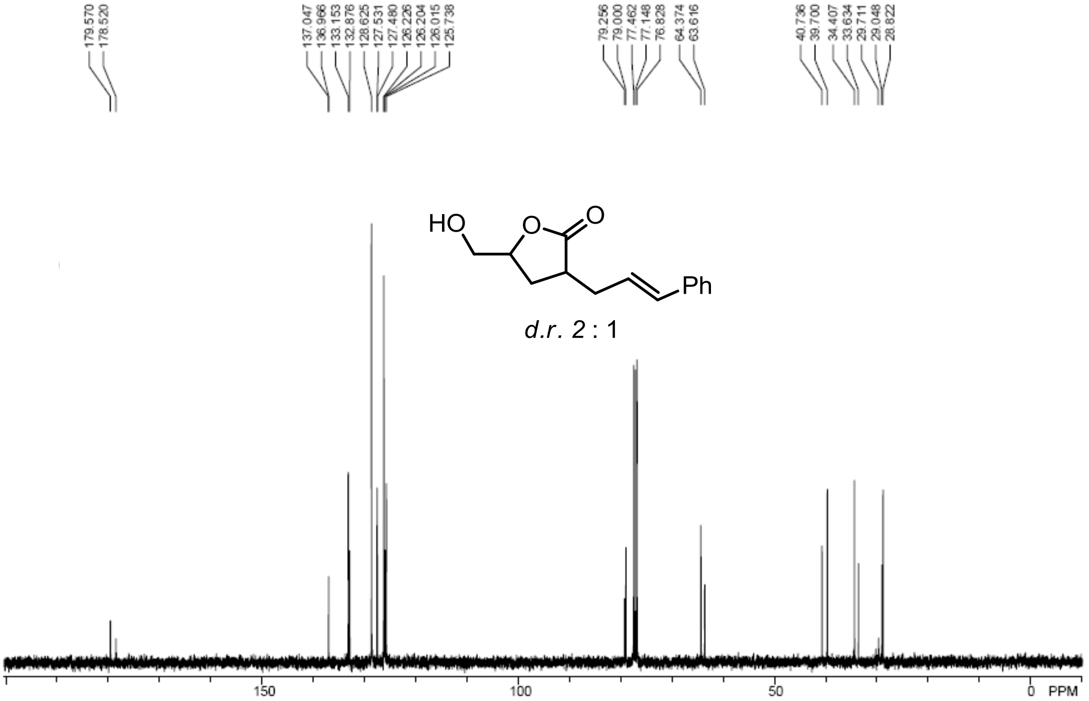

2-Allyl-4-methyl-*N*-(*p*-tolyl)pent-4-enamide (**24f**)

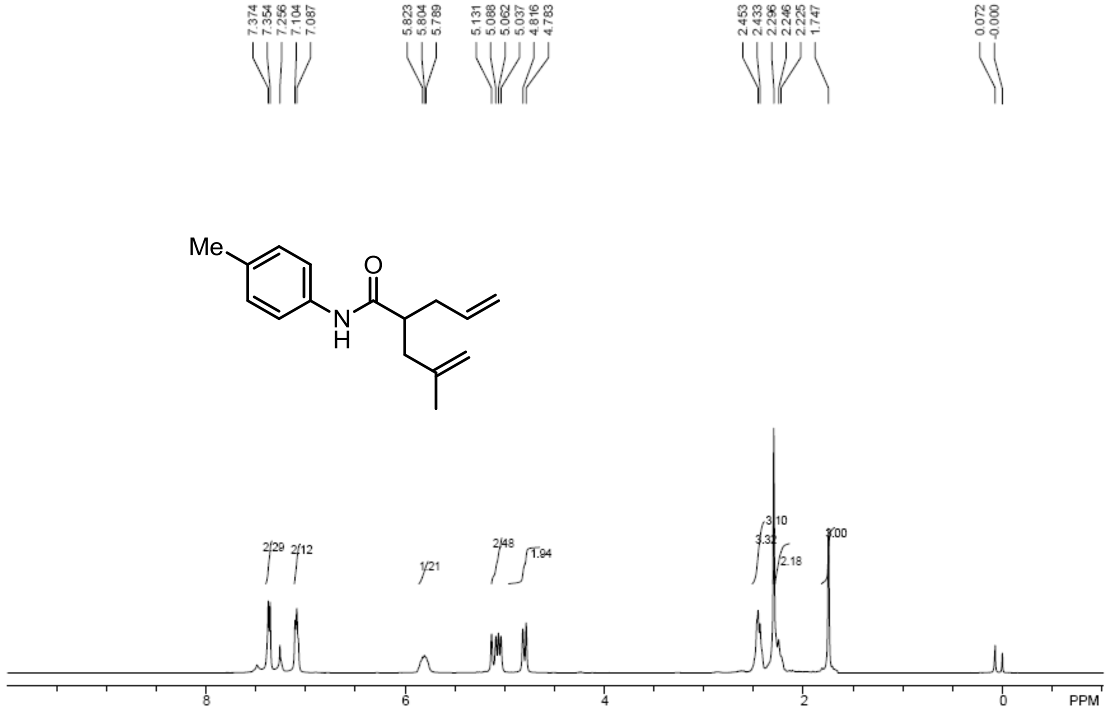

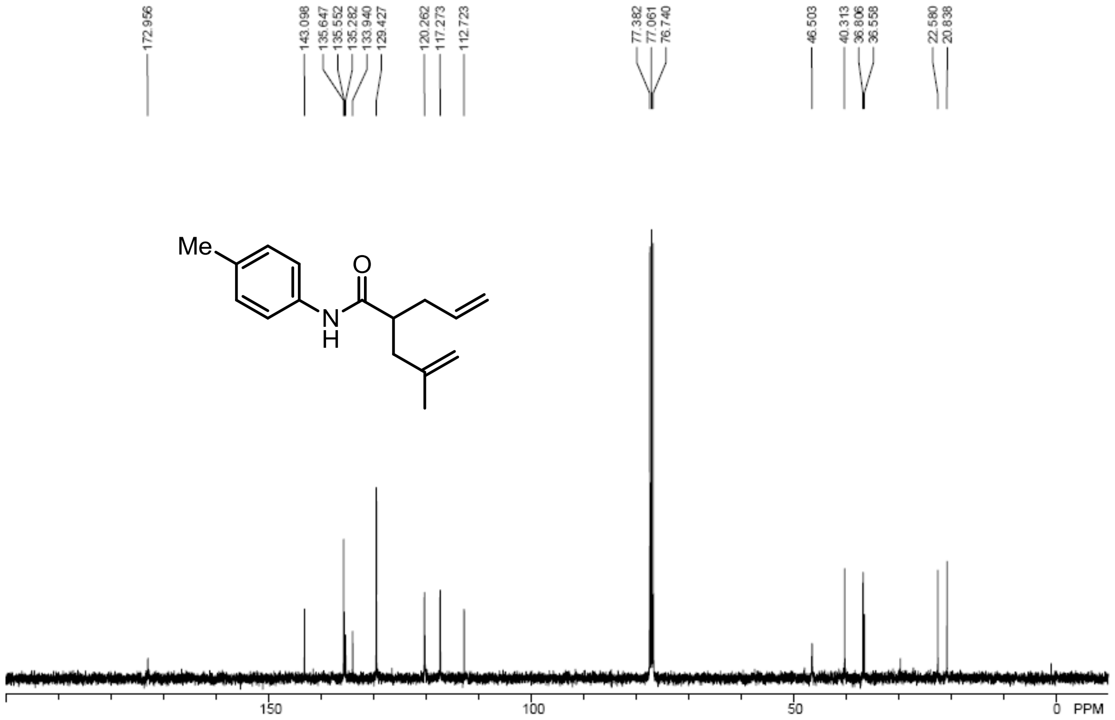

3-Allyl-5-(hydroxymethyl)-5-methyldihydrofuran-2(3*H*)-one (**25f**)

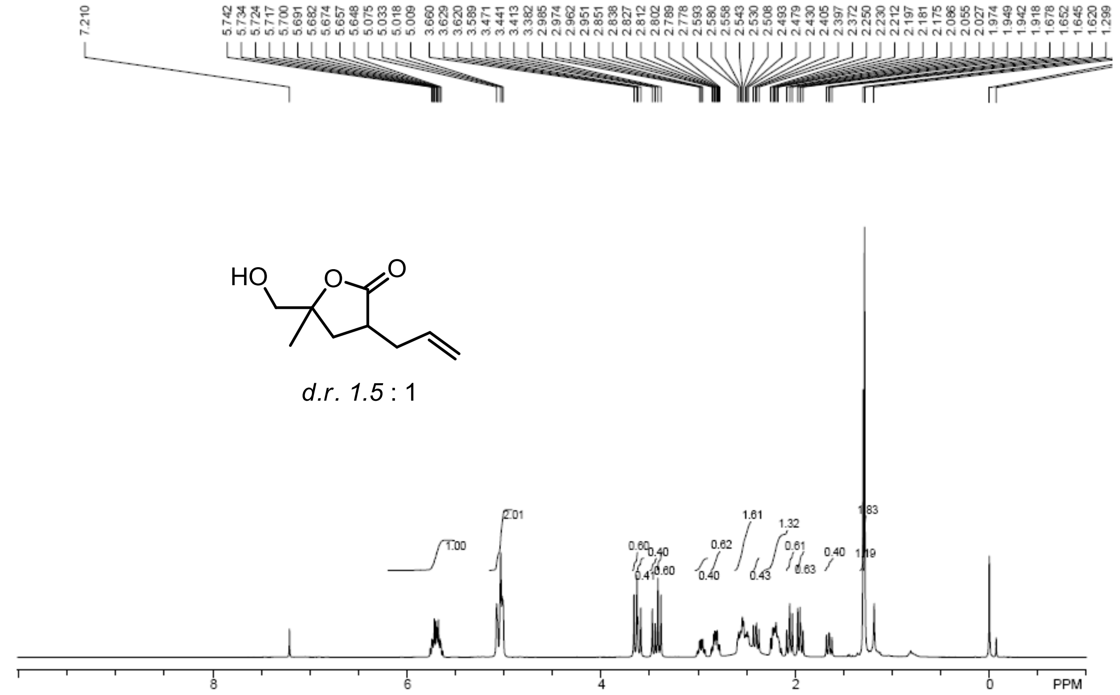

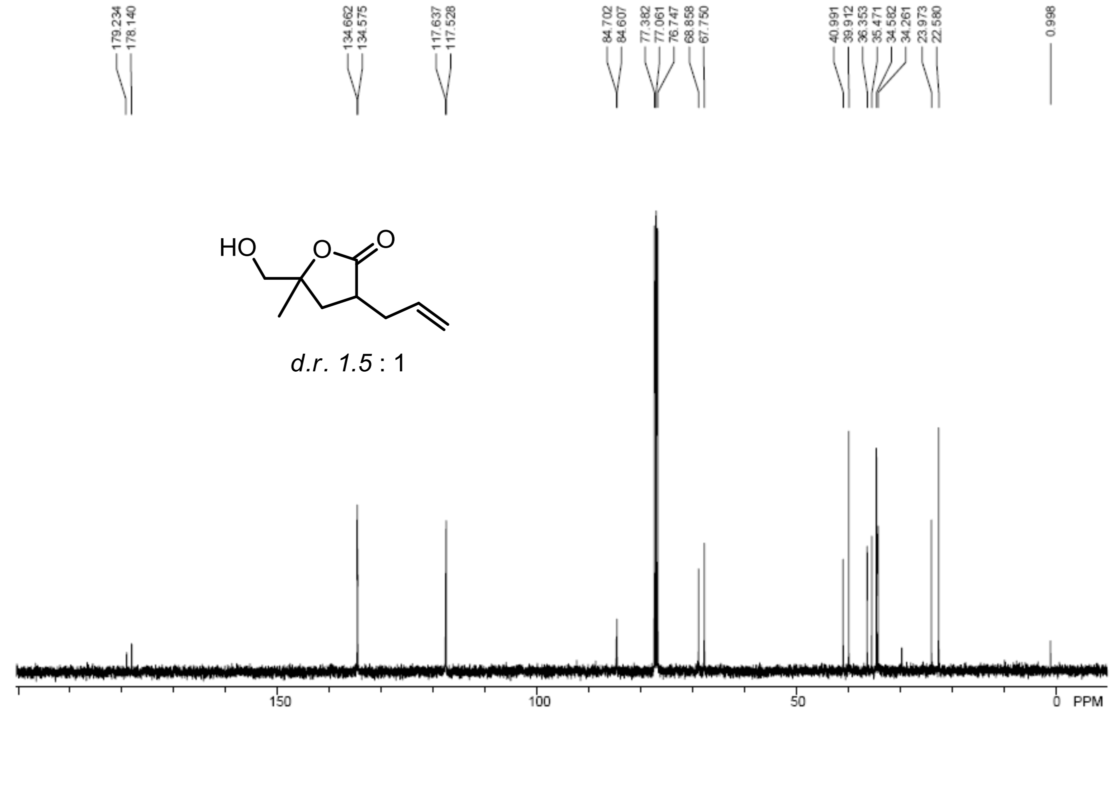

(*E*)-2-Allyl-4-methyl-5-phenyl-*N*-(*p*-tolyl)pent-4-enamide (**24c**)

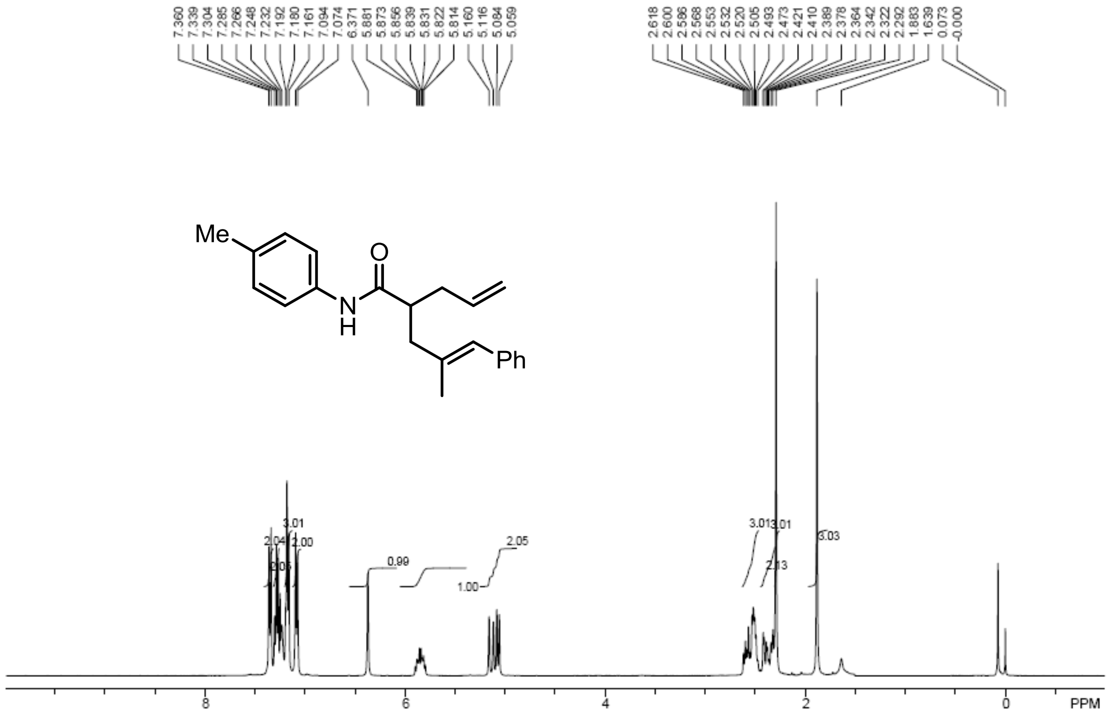

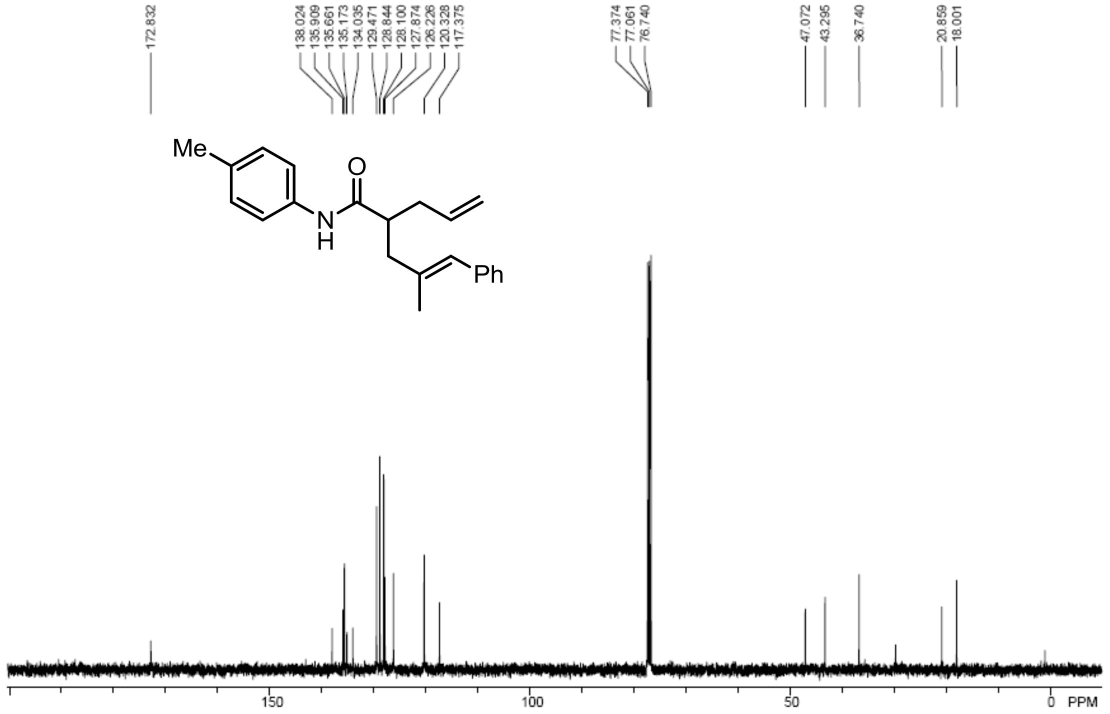

(*E*)-5-(Hydroxymethyl)-3-(2-methyl-3-phenylallyl)dihydrofuran-2(3*H*)-one (**25c**)

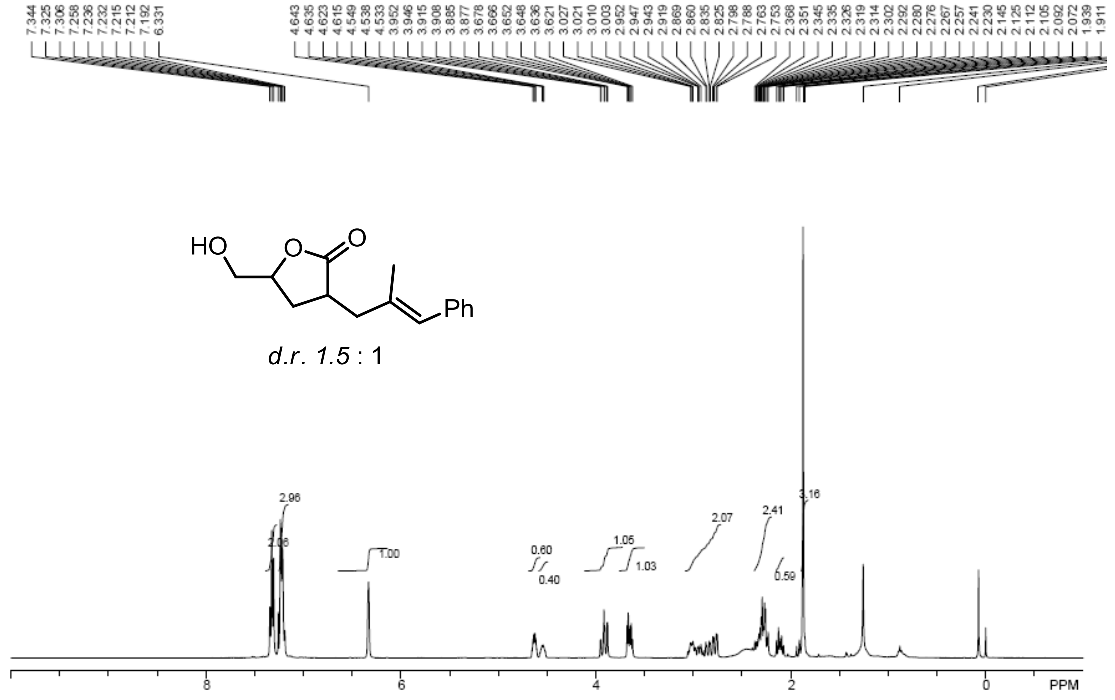

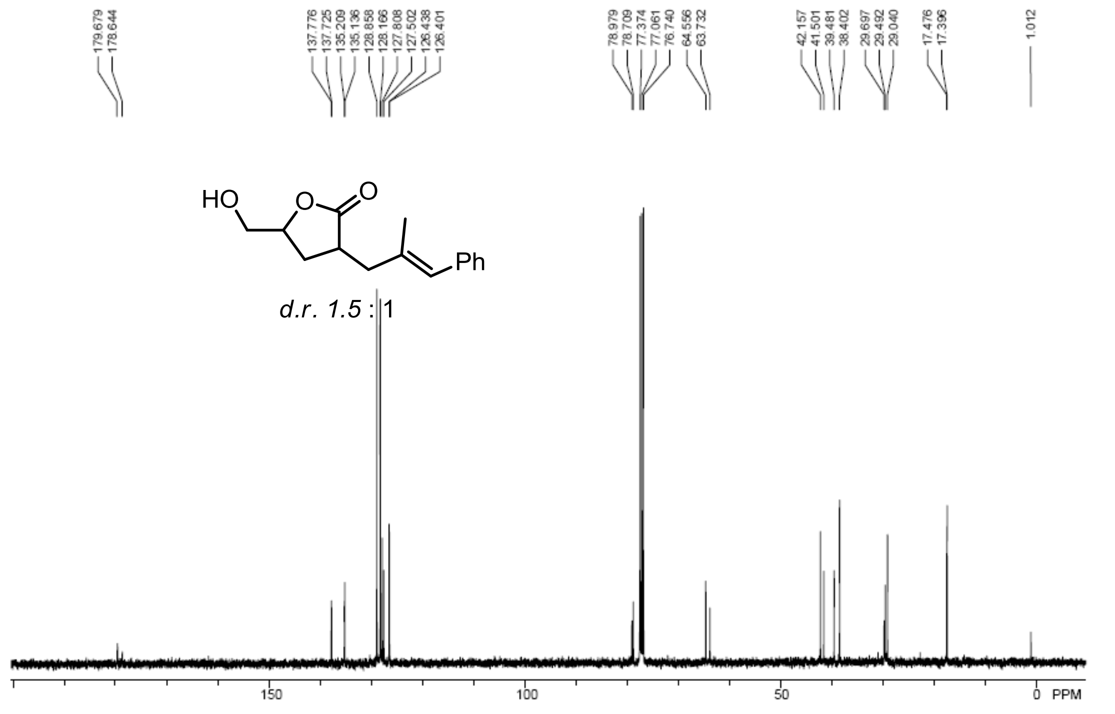

VI. References

1. Schlummer, B.; Hartwig, F. *Org. Lett*. **2002,** *4,* 1471.
2. Yong, L.; Wan, L; Oh, Y. *J. Org. Chem***.** **1999**, *64*, 7017.
3. Ooi, T.; Tayama, E.; Yamada, M.; Maruoka, K. *Synlett* **1999**, *6*, 729.
4. Kraft, P.; Bruneau, A. *Eur. J. Org. Chem.* **2007**, 2257.
5. Tellitu, I.; Urrejola, A.; Serna, S.; Moreno, I.; Herrero, M. T.; Dominguez, E.; Martin, S. M.; Correa, A. *Eur. J. Org. Chem.* **2007,** 437.
6. Sun, Y.; Fan, R. *Chem, Commun*. **2010,** *46,* 6834.
7. John, E. M.; Thomas, **L.** *J. Am. Chem. Soc.***1993,** *115,* 10167.
8. Kobayashi, K.; Fujita, S.; Nakai, D.; Fukumoto, S.; Fukamachi, S.; Konishi, H. *Helv. Chim. Acta*. **2010,** *93,* 1274.
9. Wrona, I. E.; Gozman, A.; Taldone, T.; Chiosis, G.; Panek, J. S. *J. Org. Chem***.** **2010**, *75*, 2820.
10. Schmidt, V. A.; Alexanian, E. J. *Angew. Chem. Int. Ed.* **2010,** *49,* 4491.
11. Zhao, J. F.; Loh, T. P. *Angew. Chem. Int. Ed.* **2009**, *48,* 7232 – 7235.
12. Clive, D. L. J.; Peng, J.; Fletcher, S. P.; Ziffle, V. E.; Wingert, D. *J. Org. Chem.* **2008**, *73*, 2330-2344.
13. Tellitu, I.; Urrejola, A.; Serna, S.; Moreno, I.; Herrero, M. T.; Dominguez, E.; SanMartin, R.; Correa, A. *Eur. J. Org. Chem.* **2007**, 437-444.
